# The loss of the urea cycle and ornithine metabolism in different insect orders: An omics approach

**DOI:** 10.1111/imb.12989

**Published:** 2025-03-13

**Authors:** Jessica Cristina Silva Martins, Héctor Antônio Assunção Romão, Carolina Kurotusch Canettieri, Amanda Caetano Cercilian, Patrícia Rasteiro Ordiale Oliveira, Clelia Ferreira, Walter R. Terra, Renata de Oliveira Dias

**Affiliations:** ^1^ Laboratório de Genética & Biodiversidade Instituto de Ciências Biológicas, Universidade Federal de Goiás Goiânia Goiás Brazil; ^2^ Laboratory of Gene Expression and Evolution in Arthropods Institute of Biosciences, University of Sao Paulo São Paulo São Paulo Brazil; ^3^ Laboratório de Bioquímica de Insetos Instituto de Química, Universidade de São Paulo São Paulo São Paulo Brazil

**Keywords:** arginine biosynthesis, arginine metabolism, comparative genomics, nitrogen metabolism, polyamine

## Abstract

Previous studies suggest that some insects require dietary arginine because they cannot synthesize this amino acid through the urea cycle. To determine whether this finding applies to all insects and what its metabolic implications are, we analysed the conservation of 20 genes involved in arginine biosynthesis and metabolism in the genomes of 150 species from 11 taxonomic orders. Our results showed that no insect can synthesize arginine via the urea cycle, as ornithine carbamoyltransferase is absent from all genomes analysed. While we found losses in other genes encoding urea cycle enzymes, nitric oxide synthase (*NOS*) was conserved across orders. However, the citrulline produced by NOS cannot be converted back to arginine in several insects due to the loss of argininosuccinate synthase and argininosuccinate lyase genes. Despite the inability to synthesize arginine, all insects (except some Hemiptera) can degrade it to ornithine and urea, as the arginase (*ARG*) gene is conserved across the orders analysed. For some Hemiptera that have lost *ARG*, we investigated how these insects produce or metabolize ornithine. Our results show that the genes for converting ornithine to glutamate, proline and putrescine are conserved across orders. However, while all insects have enzymes to synthesize putrescine and spermidine, some lack the ability to produce spermine due to the absence of the spermine synthase gene. Taken together, our results show that the loss of the urea cycle has led to significant changes in the pathways by which insects metabolize and recover arginine, which is particularly important for the diversification of hemipterans.

## Graphical abstract

- Among urea cycle enzymes, only the nitric oxide synthase gene is universally present across insect genomes.
- All Hemiptera species lack the enzymes needed to convert citrulline to arginine, and some also lack the pathway from arginine to ornithine.
- Putrescine and spermidine synthesis is conserved in all insects, but aphids lack the capability to produce spermine.

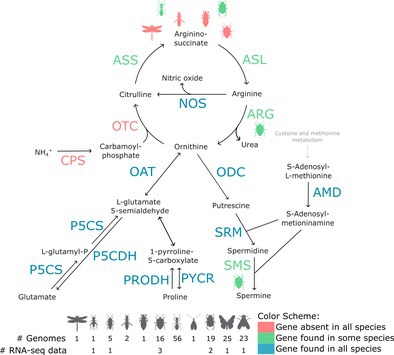

## INTRODUCTION

The nitrogen present in the amino acids is excreted mainly as ammonia, urea or uric acid. In aquatic species, ammonia can be directly diluted in the circulating water. In contrast, terrestrial animals require a pathway to convert ammonia to other less toxic and less water‐demanding nitrogen compounds, such as urea, excreted by mammals and some amphibians, and uric acid, excreted by birds, arthropods and some reptiles. In mammals, the urea cycle plays a crucial role in converting toxic ammonia to urea that is subsequently excreted by the kidneys.

**FIGURE 1:**
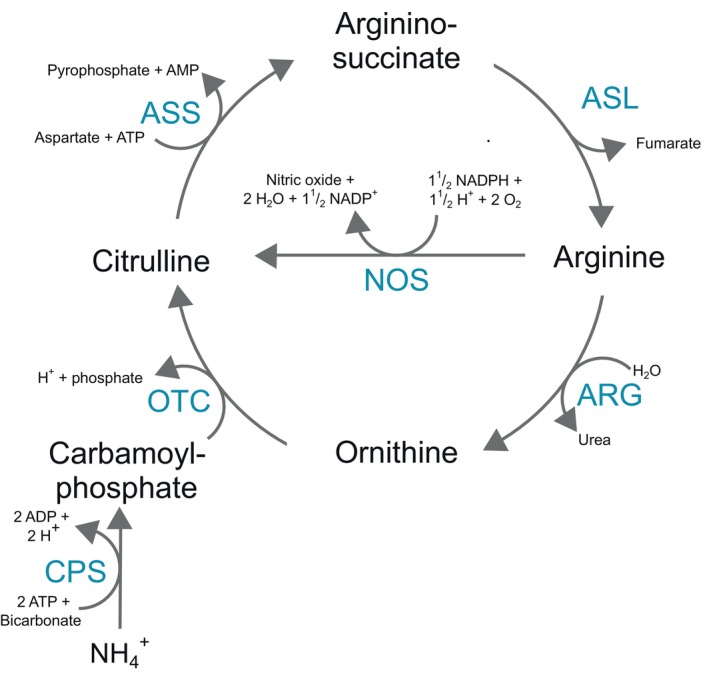
Enzymes involved in the urea cycle. The enzymes and genes represented include ARG, arginase; ASL, argininosuccinate lyase; ASS, argininosuccinate synthase; CPS, carbamoyl phosphate synthetase; NOS, nitric oxide synthase; OTC, ornithine carbamoyltransferase.

Arginine (Arg) is one of the most versatile amino acids in animal cells, playing a key role in synthesizing several vital compounds (Wu & Morris, 1998). In the urea cycle, Arg is directly converted into nitric oxide (NO) and citrulline (Cit) through a reaction catalysed by nitric oxide synthase (NOS) and into ornithine (Orn) and urea by the action of arginase (ARG). Additionally, Arg can be regenerated from Orn and Cit in most animals via the enzymes ornithine carbamoyltransferase (OTC), argininosuccinate synthetase (ASS) and argininosuccinate lyase (ASL) (Figure 1). However, the complete urea cycle has been lost in birds (Fernandes & Murakami, 2010) and insects (Reddy & Campbell, 1969), likely due to their evolutionary shift towards uricotelism. As a result, Arg has become an essential amino acid for these animals, as they can utilize it but cannot synthesize it.

The loss of the urea cycle in insects was accompanied by the loss of various genes throughout the evolutionary history of different lineages. In a comparative genomic analysis of 27 insect species, Panfilio et al. (2019) found that genes encoding all urea cycle enzymes were present only in four sampled Lepidoptera species because they did not find the *OTC* gene in the remaining species. This result agrees with different studies that showed that in insects, Arg can be replaced by Cit but not by Orn (Davis, 1962; Hinton, 1956; Reddy & Campbell, 1969). Panfilio et al. (2019) also proposed that the inability to synthesize Arg likely originated early in the Hemiptera lineage, followed by a convergent loss of the ability to degrade this amino acid in both aphid and *Rhodnius* lineages, potentially due to the low prevalence of Arg in their diets. Moreover, according to the authors, some lineages also lost *ASS* or *ASL* genes, meaning that these insects can still produce NO and Cit from Arg but lose the capability of recovering Arg from Cit.

In the absence of a complete urea cycle and the inability to regenerate Arg from Cit after NO production, it is plausible to hypothesize that the loss of *ARG* may have been advantageous for certain Hemiptera species, preventing the degradation of this amino acid. However, without *ARG*, how do these insects synthesize the Orn required for polyamine biosynthesis? While proline (Pro) and glutamate (Glu) can be synthesized through alternative pathways, putrescine in insects can only be produced from Orn via the action of ornithine decarboxylase (ODC, EC: 4.1.1.17) (Cayre et al., 1995). Consequently, in the absence of *ARG*, these insects must either synthesize Orn from Glu or Pro or acquire it from external sources to ensure the production of polyamines.

Orn is often described as being present in different organisms at lower concentrations than Glu, Pro and Arg, probably due to the rapid metabolic flux of nitrogen through this amino acid (Majumdar et al., 2015). Knock‐down of *ODC* severely impaired the survival, nitrogen metabolism, oviposition and fecundity of both sugar‐ and blood‐fed *Aedes aegypti* mosquitoes, demonstrating that polyamine homeostasis is critical for the survival of these insects (Isoe et al., 2022).

Putrescine and other polyamines are small, organic cationic molecules of two to four amine groups linked by methylene chains. In mammals, polyamines are synthesized in all tissues through the decarboxylation of Orn, which can be sourced from the urea cycle, diet or intestinal bacteria (Gerner & Meyskens, 2004). The decarboxylation of Orn by ODC produces the diamine putrescine, which forms the triamine spermidine and tetramine spermine by adding aminopropyl groups. This aminopropylation process relies on methionine metabolism, with aminopropyl groups supplied by decarboxylated *S*‐adenosylmethionine through the catalytic action of adenosylmethionine decarboxylase (AMD, EC 4.1.1.50) (Wallace et al., 2003). Polyamines are critical in several physiological processes, including cell growth, differentiation and programmed cell death. In insects, putrescine, spermidine and spermine are present throughout all developmental stages of *Musca domestica* and *Dysdercus koenigii* (Joseph & Baby, 1988). Moreover, putrescine has been shown to play an essential role in neuroblast division, acting as a transducer of juvenile hormone signals in adult crickets (Cayre et al., 1997). In flies, ionotropic chemosensory receptors mediate the attraction of Drosophila to polyamine‐rich diets, which can be beneficial and increase their reproductive success (Hussain et al., 2016). Endogenous and exogenous spermidine levels also induce autophagy in flies, increasing their longevity (Eisenberg et al., 2009). In light of this, we question whether the loss of ARG in some Hemiptera species may have also impaired their ability to synthesize or metabolize Orn, ultimately affecting their capacity to produce polyamines.

To investigate the implications of the divergent loss of urea cycle enzymes in insects, we analysed the presence of genes related to this pathway in the genomes of 150 insect species and analysed their expression profiles in the midgut and carcass tissues of nine model species. Our findings reveal that none of the insect species studied can produce all urea cycle enzymes and that several lineages have lost the ability to regenerate Arg from Cit. Furthermore, we demonstrated that despite the loss of *ARG* in some Hemiptera, all species within this order retain the ability to metabolize Orn to synthesize polyamines, Glu or Pro. This is possible because all the enzymes required for these processes are present in their genomes, except spermine synthase (SMS), which is absent in aphids. Together, our results demonstrate that while several losses of urea cycle‐related enzymes occurred across different insect groups, these losses did not compromise the ability of these insects to metabolize Orn and polyamines.

## RESULTS

### Taxonomic distribution and gene expression of the enzymes involved in the urea cycle

**FIGURE 2:**
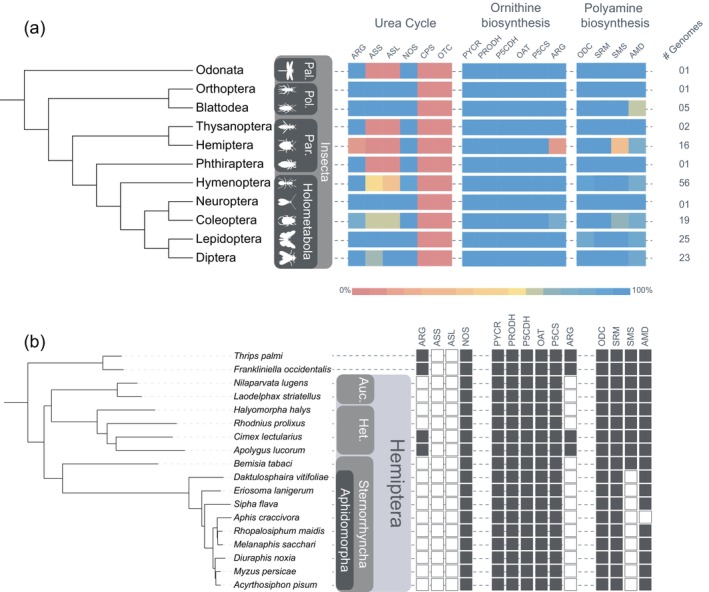
Taxonomic distribution of genes involved in the incomplete urea cycle, ornithine biosynthesis and metabolism and polyamine biosynthesis across (a) insect orders and (b) Hemiptera species. In the Hemiptera phylogeny, *Thrips palmi* and *Frankliniella occidentalis* (Thysanoptera) are used as outgroup species. The heatmap represents the percentage of genomes within each order that contain the corresponding gene, with values indicated by the colour scale below the chart. In the taxonomic tree, abbreviations include: Auc., Auchenorrhyncha; Het., Heteroptera; Pal., Palaeoptera; Par., Paraneoptera; Pol., Polyneoptera. The enzymes and genes represented are as follows: AMD, adenosylmethionine decarboxylase; ARG, arginase; ASL, argininosuccinate lyase; ASS, argininosuccinate synthase; CPS, carbamoyl phosphate synthetase; NOS, nitric oxide synthase; OAT, ornithine aminotransferase; ODC, ornithine decarboxylase; OTC, ornithine carbamoyltransferase; P5CDH, delta‐1‐pyrroline‐5‐carboxylate dehydrogenase; P5CS, delta‐1‐pyrroline‐5‐carboxylate synthetase; PRODH, proline dehydrogenase; PYCR, pyrroline‐5‐carboxylate reductase; SMS, spermine synthase; SRM, spermidine synthase.

The taxonomic distribution of the urea cycle enzymes among all Arthropoda lineages was analysed. Figure 2 shows the distribution of these enzymes among the 11 insect orders examined. Additionally, the number of genes identified in each genome is detailed in Supplementary Material 1, including genes absent from genome annotations but predicted using the BITACORA software (Vizueta et al., 2020) (Supplementary Material 2).

The formation of Cit from Orn and carbamoyl phosphate is promoted by the enzyme ornithine carbamoyltransferase (OTC, EC number: 2.1.3.3, KO number: K00611). According to the KEGG annotation, *OTC* homologous genes were found in the genomes of 6 Coleoptera, 25 Lepidoptera, 3 Hemiptera, 2 Thysanoptera, 1 Phthiraptera and 1 Odonata, as well as in 1 Collembola and 1 Thecostraca species. However, while 96.1% of the sequences identified in KEGG as OTC (KO: K00611) have both the active site residues D_263_ and C_303_ (according to the OTC protein sequence of *Bos taurus*, available in the UniProt database with the ID: Q9N1U7), all sequences from Arthropoda species included in this KO do not have these residues at these positions. In addition to D_263_ and C_303_, the positions R_141_, H_168_, Q_171_, L_304_ and R_330_—described as part of the catalytic site of the *H. sapiens* OTC gene (Shi et al., 2000)—and the positions S_55_, R_57_, T_58_, R_106_, L_128_, H_133_, Q_136_, M_236_, S_235_ and R_319_, associated with the substrate binding site of *E. coli* OTC (Langley et al., 2000), which are conserved in bacteria, fungi, plants and other Metazoan species (Supplementary Material 3), are also absent in these insect sequences. Furthermore, besides the grouping in the same KEGG orthologous group, a BLASTp search using the reference sequence from *B. taurus* as a query and the arthropods putative *OTC* genes annotated in KEGG as subjects, returned as the best hit a *Frankliniella occidentalis* sequence (KEGG identifier: foc:113203376). However, this sequence shares only 27.43% of the identity with the query. These results suggest that Arthropoda sequences annotated as OTC in KEGG are unlikely to act as such due to the absence of both active site residues, which are highly conserved in OTC sequences from most taxonomic groups. However, it is noteworthy that the tridimensional structure predicted for *Helicoverpa armigera* putative OTC using AlphaFold2 (Jumper et al., 2021) suggests a possible shared ancestry (Supplementary Material 4). Further studies are needed to understand better whether these genes play another functional role. In addition, according to the KEGG annotation, arthropods also do not have the gene encoding carbamoyl‐phosphate synthetase (CPS 1). Therefore, the complete urea cycle is missing in all insects, including Lepidoptera.

The conversion of Cit to Arg involves two steps: the conversion of Cit to argininosuccinate catalysed by ASS and the conversion of argininosuccinate to Arg plus fumarate catalysed by ASL. The number of *ASS* and *ASL* homologous genes found in each Insecta genome is shown in Supplementary Material 1. Our results show that the genomes of 92 insect species have both enzymes, 12 have only *ASS*, 3 have only *ASL* and 43 have neither. Among the insect taxonomic orders, both genes were missing in the genome annotations of all species of Odonata, Thysanoptera and Phthiraptera analysed. They were also missing from the genomes of some of the Hymenoptera, Coleoptera, Diptera and Hemiptera species. Among these orders, Hemiptera showed the most random distribution of these genes, with the Auchenorryncha *Nilaparvata lugens* having both genes, the Heteroptera *Rhodnius prolixus* having only ASS and the Sternorrhyncha species *Aphis craccivora* and *Acyrthosiphon pisum* having only *ASL*. Since these two enzymes work together in this conversion, we tested the possibility of the *R. prolixus ASS* gene being a result of contamination of its genome. For this, we searched for homologous sequences in the NCBI database for the protein sequences of the two flanking proteins of the putative ASS gene annotated in the *R. prolixus* scaffold KQ036543 using BLASTp and also tested the expression values of these three genes in our RNA‐seq analysis. Our results showed that this scaffold is probably a sequence from a bacterium belonging to the order Enterobacterales present in the middle portion of the midgut of *R. prolixus* (Supplementary Material 5). Therefore, to test whether all Hemiptera might have lost the ability to synthesize Arg from Cit, we assessed the possibility of contamination in all *ASS* and *ASL* sequences annotated in Hemiptera genomes. The *Ac. pisum* ASL sequence (XP_029347774.1) and the genome scaffold for which this sequence was annotated (NW_021764953.1), as well as the *N. lugens* ASS (XP_039301184.1) and ASL (XP_039301185.1) sequences, both annotated in the same genome scaffold (NW_024092249.1), were removed from NCBI databases as possible contaminations. The *Ap. craccivora* ASL sequence (KAF0746383.1) is still available in the NCBI, but as for *R. prolixus*, all sequences from the scaffold containing the suspicious sequence (VUJU01007234.1) have Enterobacterales sequences as the best hit (Supplementary Material 6), showing that this scaffold is probably also the result of a genome sequence contamination. Thus, all Hemiptera probably lost their ability to form Arg from Cit. To search if this contamination problem happened only in the Hemiptera genomes, and since both *ASS* and *ASL* are needed to produce Arg, we tested the probability of genome contamination in all insect genomes containing only one of these genes. Of the 14 organisms analysed, two sequences have already been removed from NCBI as possible contaminations, and 12 have insect sequences as the best hits, so they are probably not a contamination (Supplementary Material 7). Thus, for these species, only one gene is present, or the missing gene is simply not annotated in this version of the species genome.

Arg can be converted to Orn by the action of ARG in the urea cycle or to Cit and NO by the action of the NOS (Figure 1). At least one ARG gene was conserved in all insect orders except Hemiptera. In Hemiptera, a gene encoding for this enzyme was found only in the species *Apolygus lucorum* (Heteroptera, Cimicomorpha, Miridae) and *Cimex lectularius* (Heteroptera, Cimicomorpha, Cimicoidea). To verify if ARG may be present in other Hemiptera not sampled in our genome analysis, we performed a BLASTp search against the non‐redundant protein database from NCBI, using the sequences of these two Hemiptera species as queries. We found protein sequences similar to them in three other Hemiptera species, the Heteroptera *Nesidiocoris tenuis* (Cimicomorpha, Miridae) and the Auchenorryncha species *Homalodisca vitripennis* and *Macrosteles quadrilineatus* (both, Cicadomorpha, Cicadellidae) (Supplementary Material 8). Interestingly, the most closely related species in our analysis, such as *R. prolixus* (Heteroptera, Cimicomorpha, Reduvioidea) and the Auchenorrhyncha species *Laodelphax striatellus* and *N. lugens* (both, Fulgoromorpha; Fulgoroidea; Delphacidae), lacked annotated ARG genes. This suggests that not all species within the Heteroptera Cimicomorpha have lost ARG, and despite its absence in the two Auchenorrhyncha Fulgoromorpha species analysed, ARG is still present in at least two Cicadomorpha species.

According to KEGG, there are two types of NOS in Arthropoda: homologous to the brain (K13240) or inducible (K13241) human *NOS* genes. However, the putative inducible *NOS* gene was annotated in KEGG for only six Arthropoda species, the insect *Bradysia coprophila* (Diptera), one Brachiopoda, three Arachnida and one Merostomata species. Therefore, our results probably represent the number of genes homologous to the *Homo sapiens NOS* that produce NO in the brain and nervous system (K13240). This gene was found in the genomes of species from all the insect taxonomic orders analysed (Supplementary Material 1). In most orders, several species have only one copy of this gene, but it is noticeable that all Lepidoptera species have at least two copies of this gene. According to the *Spodoptera frugiperda* RNA‐seq analysis, both genes were associated with higher TPM levels in the carcass sample than in any midgut part of the larvae analysed (Supplementary Material 9), which may indicate that these genes are involved in NOS production in the nervous system of this species.

### Alternative paths to ornithine biosynthesis

Most Hemiptera species have lost the ability to synthesize Orn from Arg. Therefore, we examined the available pathways to produce and utilize Orn in this and other insect groups.

One way of synthesizing Orn is from Glu, using a pathway that has aminoacylase (ACY) as the final step. This enzyme is present in all insect groups. Nevertheless, according to KEGG, the enzymes involved in synthesizing *N*‐acetylornithine from Glu are absent in any arthropod genome. So, this aminoacylase is likely involved in the hydrolysis of other *N*‐acetylated amino acids in acetate and free amino acids.

**FIGURE 3:**
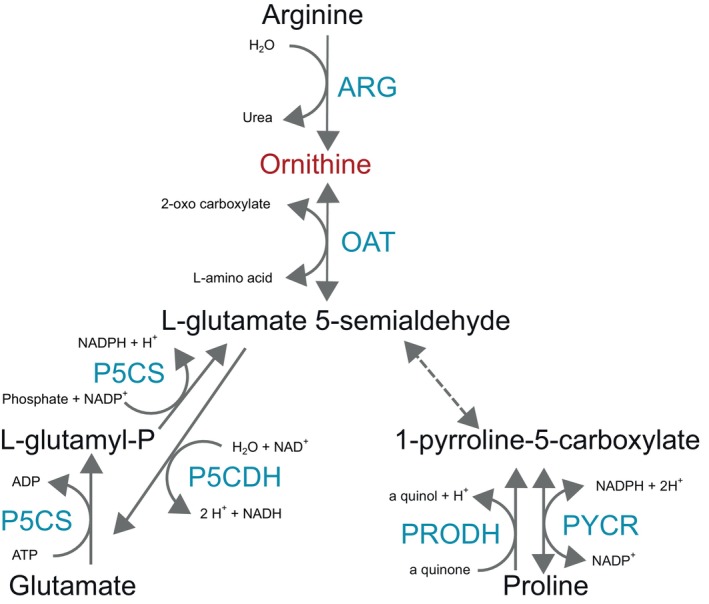
Enzymes involved in the ornithine biosynthesis and metabolism. Arg, arginase; OAT, Ornithine aminotransferase; P5CDH, delta‐1‐pyrroline‐5‐carboxylate dehydrogenase; P5CS, delta‐1‐pyrroline‐5‐carboxylate synthetase; PRODH, proline dehydrogenase; PYCR, pyrroline‐5‐carboxylate reductase.

Orn can be synthesized from Glu through another pathway and from Pro, as described in Figure 3. Orn production from Glu and Pro involves the formation of l‐glutamate 5‐semialdehyde, which can be interconverted to Orn by the action of the enzyme ornithine aminotransferase (OAT). This enzyme was found in all insect orders analysed (Supplementary Material 1), showing that even the Hemiptera species that lost ARG can synthesize Orn from or convert it to Glu and Pro.

The irreversible synthesis of l‐glutamate‐5‐semialdehyde from Glu involves the conversion of Glu to l‐glutamyl‐*P* and of l‐glutamyl‐*P* to l‐glutamate‐5‐semialdehyde, catalysed by the enzyme Delta‐1‐pyrroline‐5‐carboxylate synthase (P5CS), a bifunctional enzyme that has Glu kinase and ∂‐Glutamyl‐*P* reductase activities. This enzyme was also found in the genome annotation of insects from all insect orders analysed in this work (Supplementary Material 1). The synthesis of l‐glutamate from l‐glutamate 5‐semialdehyde can be produced by the action of the enzyme Delta‐1‐pyrroline‐5‐carboxylate dehydrogenase (P5CDH). This enzyme was also found in the genome annotation of insects from all insect orders analysed in this work (Supplementary Material 1). The enzymes involved in the interconversion of Glu to l‐glutamate 5‐semialdehyde are present in the genomes of all insect species analysed, and their importance may be because they are essential in the interconversion of Glu and Pro. Consistent with this, conservation in all analysed insect genomes was also found for the pyrroline‐5‐carboxylate reductase (PYCR), which converts Pro to 1‐pyrroline‐5‐carboxylate, and the proline dehydrogenase (PRODH), which converts Pro to 1‐pyrroline‐5‐carboxylate (Figure 2), likely due to the critical role of proline in insect metabolism, particularly in winged insects, where it serves as an energy substrate for flight (Scaraffia & Wells, 2003; Teulier et al., 2016).

Taken together, our results show that besides the lack of ARG, all Hemiptera species can potentially produce Orn from Glu or Pro and use Orn to produce these two amino acids.

### The role of ornithine in polyamine synthesis

**FIGURE 4:**
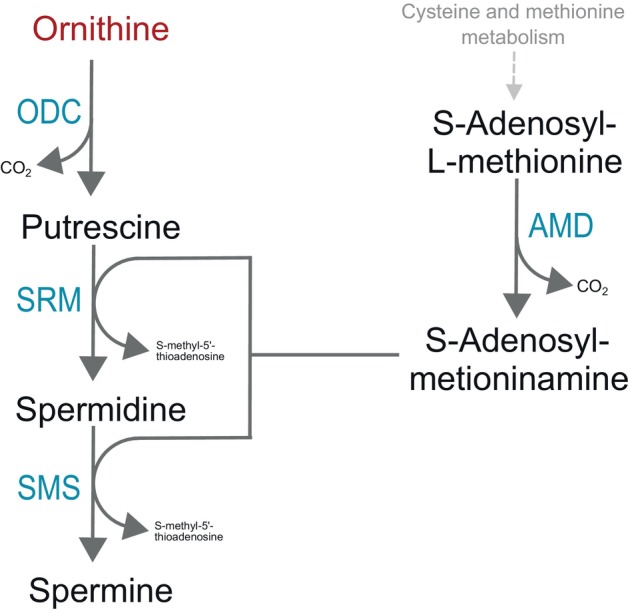
Enzymes involved in polyamine biosynthesis. AMD, adenosylmethionine decarboxylase; ODC, ornithine decarboxylase; SMS, spermine synthase; SRM, spermidine synthase.

Besides the possibility of its conversion to Glu or Pro, Orn can also form polyamines (Figure 4). Orn can be converted to putrescine by ODC. The gene encoding this enzyme was found in the genomes of more than 90% of the analysed species from all insect orders, and in several of them, multiple copies were found. For example, multiple copies of *ODC* were found in the genomes of mosquitoes, some Coleoptera, some Hymenoptera and the Orthoptera *Schistocerca americana* (Supplementary Material 1).

The enzyme spermidine synthase (SRM, EC number: 2.5.1.16, KO number: K00797), which catalyses the conversion of putrescine to spermidine, is conserved in the genomes of at least 90% of the species from all insect orders analysed (Supplementary Material 1).

The last polyamine that can be formed in this pathway is spermine, which is formed from spermidine by the enzyme SMS. This enzyme is conserved in the genomes of at least 90% of the species from all insect orders except Blattodea, Hemiptera and Coleoptera (Figure 2). Our results showed that Hemiptera Sternorrhyncha, from the infraorder Aphidomorpha, may not synthesize spermine but only putrescine and spermidine.

### Expression analysis of the genes involved in the biosynthesis and metabolism of arginine and ornithine

To better understand the role of the analysed genes in insect metabolism, we searched for their expression profiles in different digestive tissues of insects from different taxonomic orders. For this, we used previously obtained RNA‐seq results from each tissue sampled for adults of *Periplaneta americana* (Blattodea) and *R. prolixus* (Hemiptera), and larvae of *Tenebrio molitor* (Coleoptera), *Mu. domestica* (Diptera) and *S. frugiperda* (Lepidoptera) (Supplementary Materials 9–13). Moreover, we also used RNA‐seq analysis conducted for different digestive tissues and carcasses of adults of *Abracris flavolineata* (Orthoptera), *Mahanarva fimbriolata* (Hemiptera, Auchenorrhyncha), *Dysdercus peruvianus* (Hemiptera, Heteroptera) and larvae of *Dermestes maculatus* (Coleoptera), for which no genome was available (Supplementary Materials 14–17).

To compare the RNA‐seq results for all enzymes involved in the incomplete urea cycle (Figure 1), we summed the TPM values for genes encoding the same enzyme (Supplementary Material 18). We had RNA‐seq data for the Malpighian Tubules (MT) in only two insects: *P. americana* and *A. flavolineata*. In both cases, this tissue showed higher expression levels (TPM) in comparison to the remaining body samples for one of the two genes involved in the conversion of Cit to Arg, *ASS* in *P. americana* and *ASL* in *A. flavolineata*. For the other insect species, we sampled the midgut, while all remaining body parts (excluding heads, wings and legs) were categorized as ‘carcasses’. Thus, in these species, MT was grouped with other carcass tissues. All other species exhibited higher TPM values for *ASS* and *ASL* in the carcass samples, which may also be related to the role of MT in this enzyme production.

*NOS* genes had TPM values below 15 in all samples from *P. americana*, *R. prolixus*, *M. fimbriolata*, *M. domestica* and *S. frugiperda*, suggesting that this gene may be constitutively expressed at low levels in these species in the experimental conditions used in our work. However, it is noteworthy that *NOS* plays a significant role in the midgut of larvae from different Coleoptera species, as high TPM values were observed in the midgut samples of *T. molitor* and *D. maculatus*. In *D. maculatus*, a high expression value was also detected in the carcass sample. In contrast, in *A. flavolineata*, *NOS* showed higher TPM values in the MT sample, a pattern not observed in *P. americana*, where *NOS* was undetectable in this tissue. For *D. peruvianus*, high *NOS* expression was observed in the carcass sample, which contrasts with the low expression levels found in the other Hemiptera species analysed in our work (*R. prolixus* and *M. fimbriolata*).

*ARG* genes exhibited higher TPM values in the carcass samples of all insects, except for *A. flavolineata*, which showed negligible expression levels across all samples; the hemipteran species *R. prolixus*, which lacks this gene; and *M. fimbriolata*, where *ARG* TPM values were consistent across all tissue samples, with no significant differences observed.

Since some Hemiptera species, such as *R. prolixus*, lack the *ARG* gene, they are likely unable to synthesize Orn from Arg. To explore whether alternative pathways for Orn biosynthesis exist in these insects, we searched for genes involved in Orn production and metabolism within their genomes and our RNA‐seq data. Our analysis of *R. prolixus* revealed that *OAT* and *ODC* genes were expressed in all tissues examined. Notably, *ODC* displayed TPM values at least 1.92 times higher than *OAT* across all tissues, especially in the carcass, in which *ODC*'s TPM values were 5.66 times higher than the combined TPM values of both *OAT* genes. These results suggest that Orn is likely used for putrescine production under the experimental conditions, potentially sourced from L‐glutamate 5‐semialdehyde or through dietary or symbiotic origins. In *D. peruvianus*, *ODC* also showed higher TPM values—more than 3.57 times that of *OAT* across all tissues. In *M. fimbriolata*, TPM values for *ODC* were at least 1.38 times higher than *OAT* in all tissues.

In the adults of *P. americana*, among the genes involved in Orn biosynthesis and metabolism analysed in our study (Figure 3), *P5CDH* exhibited the highest TPM values across all sampled tissues, except in the foregut and carcass, where *ODC* and *OAT* were the most highly expressed genes, respectively (Supplementary Material 19). Similarly, *P5CDH* was the most highly expressed gene in the Malpighian tubules of adult *A. flavolineata*. However, in all other samples, *P5CS* showed values equal to or higher than *P5CDH*, indicating that in these cases, the conversion of Glu to Pro might hold equal or greater significance compared to Glu biosynthesis through this pathway.

To analyse the conversion of Orn to polyamines, we compared the TPM values of *ODC*, *SRM*, *SMS* and *AMD* in our RNA‐seq data (Supplementary Material 20). Among these genes, *ODC* had the highest TPM values across all tissues in *P. americana*, *R. prolixus*, *T. molitor*, *A. flavolineata*, *D. maculatus*, *D. peruvianus* and *M. fimbriolata*. In *Mu. domestica* larvae, *ODC* was also the most expressed gene in the third portion of the posterior midgut (PM3) and the carcass (CAR). However, all four genes, including *ODC*, showed low expression levels in the remaining samples. In *S. frugiperda* larvae, *ODC* was the most highly expressed gene in all midgut portions, but in the carcass, *AMD* had a TPM value 1.94 times higher than *ODC*. Together, these results highlight the significance of putrescine biosynthesis from Orn in all the insects analysed.

## DISCUSSION

### The urea cycle is absent in arthropods

Analysing the genomes of 27 insect species, Panfilio et al. (2019) found that only the Lepidoptera species had all genes involved in the urea cycle in their genome. However, when we searched the KEGG database for the ornithine transcarbamoylase (OTC) sequences, we found sequences from 40 Arthropoda species, including sequences from 25 Lepidoptera species, but none of them had the catalytic site described for OTC. Consistent with this finding, OTC activity was not detected in the fat body of the cockroach *Blaberys cranifera* and the silkmoth *Hyalophora gloveri* (Reddy & Campbell, 1977). Thus, we believe that none of the Arthropoda species have the complete urea cycle since they also lack the *CPS 1* gene in their genomes, which would impair the synthesis of the carbamoyl‐phosphate used in the first step of the cycle.

The absence of a complete urea cycle has several implications, starting with the impossibility of synthesizing Arg, which is an essential amino acid not only because of its requirement in protein synthesis but also because of its involvement in NO and Orn synthesis, which can be used in the synthesis of Pro, Glu and polyamines (Fernandes & Murakami, 2010).

### Arginine metabolism

The genomes of all insects analysed in our work preserve at least one copy of the gene encoding for NOS, an enzyme that uses Arg, molecular oxygen and NADPH as substrates to produce Cit and NO. NO is an important signalling molecule in the nervous system of diverse organisms, including mammals and insects. In insects, NO is also involved in the immune response, acting as an effector to kill pathogens or regulating the production of antimicrobial peptides (Chen et al., 2022).

In some Hemiptera, all urea cycle enzymes were lost but not the *NOS* gene, highlighting the importance of this gene for the survival of these animals. Examples of the *NOS* relevance to Hemipterans were obtained from the fact that NOS activity was detected in the bacteriocytes that host the *Buchnera aphidicola* symbionts in *Magoura viciae* (Sternorrhyncha: Aphididae) (Ganassi et al., 2005) and in the salivary glands of the hematophagous *R. prolixus* (Heteroptera: Reduviidae), in which NOS also acts as a vasorelaxing molecule (Ribeiro & Nussenzveig, 1993).

Two enzymes, ASS and ASL, are involved in the conversion of Cit to Arg. In our analysis, both *ASS* and *ASL* genes were missing in the genomes of 28.4% of the insects analysed, while 9.9% had only one of these genes. Our analysis suggests that no Hemiptera species contains both *ASS* and *ASL* genes in its genome. In cases where one of these genes was annotated in certain species, our findings indicate that the gene may originate from bacterial DNA. However, we cannot conclude whether these annotations are due to contamination or horizontal gene transfer (HGT), a phenomenon documented in some insects (Dhaygude et al., 2019; Li et al., 2022). In contrast, our analysis found no signs of genomic contamination for some Hymenoptera and Diptera species, where only one of the two genes was identified. This leads us to hypothesize that these insects either utilize only one of the genes or, more likely, their genome sequencings are incomplete, which could explain the absence of one gene.

The absence of *ASS* and *ASL* in 28.4% of the insect genomes analysed suggests that the loss of both genes occurred independently across different insect taxonomic groups during their evolutionary history. So why should this loss be advantageous or neutral so these groups were not counter‐selected? One hypothesis is that NOS activity is very low in these insects, reducing the demand for Arg, which is also used in protein synthesis. For the Hemiptera species with available RNA‐seq data, this seems plausible given the low *NOS* expression levels observed in *R. prolixus* (Supplementary Material 11) and *Ma. fimbriolata* (Supplementary Material 17), both of which lack detectable *ASS* and *ASL* genes. However, in the Hemiptera species *D. peruvianus*, where *ASS* and *ASL* genes are also absent, we identified five transcripts coding for putative *NOS* with high TPM values, particularly in the carcass (Supplementary Material 16), contradicting this hypothesis. An alternative hypothesis is that the last common ancestor of Hemiptera had a diet rich in Arg, making the loss of the arginine‐recovery pathway via *ASS* and *ASL* neutral or even beneficial.

The gene encoding ARG was present in all insects analysed except for Hemiptera. It is absent from the genomes of 14 of the 16 (87.5%) Hemiptera species analysed. Hemiptera have variable food sources and are composed of four suborders: Sternorrhyncha, Heteroptera, Auchenorrhyncha and Coleorrhyncha (the latter with no genome available) (Johnson et al., 2018). In our analysis, including searches against the entire NCBI protein database, genes encoding for ARG were found only in two species of Cicadellidae (Auchenorrhyncha) and two species of Cimicomorpha (Heteroptera), suggesting that the loss of *ARG* probably occurred several times along the evolutionary history of hemipterans, as this gene was likely present in the ancestors of these most derived groups. In contrast, *ARG* was not found in any of the genomes of the Sternorrhyncha species analysed, suggesting that the loss may be very basal in this lineage.

In addition to our comparative genomics analysis, we also found one transcript encoding putative ARG in the transcriptome of *Ma. fimbriolata* (Auchenorrhyncha, Cicadormorpha, Cercopidae), which was similarly expressed in all tissue samples analysed in our RNA‐seq analysis (Supplementary Material 17), and one transcript in the transcriptome of the seed‐feeding species *Dy. peruvianus* (Heteroptera, Pentatomomorpha, Pyrrhocoridae), with higher TPM levels in the carcass than in any midgut tissue (Supplementary Material 16). This result for *D. peruvianus* is consistent with the findings of Panfilio et al. (2019), who also described the presence of *ARG* in another seed‐feeding Pentatomomorpha species, *Oncopeltus fasciatus* (Lygaeidae). Taken together, the distribution of the *ARG* among Hemiptera species shows that the loss of this gene was very random among Hemiptera lineages, and it is not clear whether diet acted as a selective pressure to preserve or lose this gene.

In our RNA‐seq analyses, the only species that did not have ARG was *R. prolixus*. For this species, the gene most expressed in the Orn biosynthesis and metabolism was *PYCR*, which can interconvert Pro to 1‐pyrroline‐5‐carboxylate, followed by *ODC*, which converts Orn to putrescine (Supplementary Material 11). Notably, this relatively high expression of *ODC* shows that *R. prolixus* needs to use Orn at least to synthesize putrescine, so these insects may synthesize Orn from Pro or Glu or obtain it from its food or symbiont sources.

### Ornithine and polyamine production

All genes involved in polyamine biosynthesis were found in all insect orders, except for Hemiptera, where the SMS gene was missing in all Sternorrhyncha from the infraorder Aphidomorpha.

SMS deficiency in humans causes Snyder‐Robinson syndrome (SRS), an X‐linked intellectual disability, and in *Drosophila melanogaster*, it leads to survival deficits and synaptic degeneration due to increased spermidine catabolism (Li et al., 2017). This excessive catabolism produces toxic metabolites, such as aldehydes and reactive oxygen species (ROS) like H₂O₂, which induce oxidative stress and disrupt lysosomal functions (Li et al., 2017). However, the *SMS* loss in aphids may not cause the same problems observed in *Dr. melanogaster*, as Buchnera, a long‐standing aphid symbiont, could utilize spermidine. Therefore, understanding how aphids survive without SMS and how this loss may have influenced their symbiotic relationship with *Buchnera* remains an essential area of study. Additionally, it would be interesting to investigate whether spermine is present in any form in aphids and, if not, how they manage to survive without this polyamine.

### Expression profile of genes involved in arginine biosynthesis and metabolism

To better understand the role of enzymes in the incomplete urea cycle across different insect groups, we examined the expression profiles of genes encoding these enzymes using RNA‐seq data previously generated by our group. Our results suggest that the conversion of Cit to Arg was likely more active in the Malpighian tubules or other carcass tissues across all insects analysed where these genes were found (Supplementary Material 18). The carcass may also serve as a preferential site for converting Arg to urea, as this tissue exhibited the highest TPM values for *ARG* in all insect samples (Supplementary Material 18). Additionally, the conversion of Arg to Cit, which plays a crucial role in NO production, occurred constitutively in most of our samples. However, the expression profile of *NOS* was notably different between the two Coleoptera species included in our analysis.

*NOS* was highly expressed in the midgut samples of larvae from *T. molitor* and *D. maculatus* (Supplementary Materials 12 and 15, respectively). The NO plays a crucial role in mediating immune responses in insects. In Coleoptera, upregulation of *NOS* expression has been previously observed in the hemolymph of *Pterostichus melas italicus* (Carabidae) after exposure to *Escherichia coli* lipopolysaccharide (Giglio et al., 2015). Although our samples were not exposed to any bacterial treatment, it is known that there is a rich microbiota in *T. molitor* larvae (Genta et al., 2006). The *NOS* genes identified in these Coleoptera species may similarly contribute to immune responses in these insects. Furthermore, despite the lower expression levels observed in the midgut of other insect species sampled in our RNA‐seq analysis, we hypothesize that *NOS* expression might be induced under different experimental conditions involving microbial challenges. For example, all Lepidoptera species analysed in our study possess at least two copies of the *NOS* gene in their genomes, suggesting that this gene may play a particularly important role in these insects. Although the two *NOS* copies identified in *S. frugiperda* were not highly expressed in our experiments, *NOS* levels have been shown to increase in the midgut of other Lepidoptera, such as *Antheraea pernyi* (Saturniidae), following infection by the microsporidian parasite *Nosema pernyi* (Liu et al., 2020). In *Manduca sexta*, ingestion of the bacterium *Photorhabdus luminescens* also induced *NOS* expression in the larval gut, potentially inhibiting bacterial translocation across the gut wall (Eleftherianos et al., 2009). Therefore, we suggest that future RNA‐seq experiments involving different treatments are necessary to further elucidate the role of NOS in insect immune responses.

Regarding Orn biosynthesis and metabolism, we observed that *R. prolixus*, despite lacking the *ARG* gene and thus being unable to synthesize Orn from Arg, can still use Orn to produce putrescine, as the *ODC* gene is expressed in all tissue samples analysed (Supplementary Material 11). This suggests that *R. prolixus* likely acquires Orn from the metabolism of Pro or Glu, as *OAT* was also expressed in all samples or possibly from another external source. Additionally, the RNA‐seq data from all insect species analysed indicate that putrescine biosynthesis from Orn is a crucial metabolic step for all of them, as *ODC* is consistently expressed across all samples (Supplementary Material 19).

In conclusion, our results show that the complete urea cycle was probably absent in Insecta's last common ancestor species, as the *OTC* and *CPS* genes were not found in the insect genomes analysed. In addition, we detected subsequent losses in some of the insect lineages in other enzymes that make up the cycle. For example, the ability to recover Arg from Cit was probably lost in the last common ancestor of the Paraneoptera, as the *ASL* and *ASS* genes were not found in any Phthiraptera, Thysanoptera and Hemiptera species. In Hemiptera, other enzymes were also lost in some groups, with *ARG* found in the genomes of only a few species and *SMS* absent in Sternorrhyncha. These results show that the loss of the urea cycle was followed by several changes in Arg metabolism along the evolutionary history of insects, particularly in Hemiptera.

## EXPERIMENTAL PROCEDURES

### Acquisition of genomic data for Arthropoda species

Available arthropod genomic data (GFF files and genome assemblies, protein and CDS fasta files) were retrieved from NCBI's Genome Database, i5K, VectorBase, LepBase, FireflyBase, WaspBase, BIPAA's AphidBase, Coleoptera, LepidoDB and ParWaspDB databases between November 2021 and May 2022. Data for *P. americana* were kindly provided by Li et al. (2018). Only the longest isoform for each gene was retained for further analysis, utilizing AGAT v0.8.1 scripts (Dainat, 2022). Genome completeness was assessed using BUSCO software v5.3.0 (Manni, Berkeley, Seppey, & Zdobnov, 2021). The species included in the analyses were selected based on the following criteria: (1) having a genome completeness index >90% and a single‐copy gene index >75%; (2) having a higher genome completeness index compared to other congeneric species; (3) availability in the NCBI database when family counterparts were sourced from other databases; (4) being the sole representative of its family; (5) inclusion among the species selected for RNA sequencing (see item 4.4). Representatives of Chelicerata orders with the highest genome completeness indexes were selected as outgroup species. The genomes selected and their respective accession numbers and BUSCO statistics are listed in Supplementary Material 21.

### Arthropoda phylogenomic tree proposal

A non‐ultrametric phylogenomic tree was predicted following BUSCO's phylogenomic pipeline (Manni, Berkeley, Seppey, Simão, et al., 2021) with some adaptations, as briefly described below. Protein sequences from single‐copy orthologous genes, or the highest‐rated duplicate copies shared across all species, were aligned using MAFFT software v7.503 (Katoh & Standley, 2013) in the L‐INS‐i model with 1000 iteration cycles. Low‐quality positions in the alignments were removed using the trimAL tool with the ‐automated1 option (Capella‐Gutiérrez et al., 2009). The trimmed alignments from each orthologous group were concatenated using the catfasta2phyml script (https://github.com/nylander/catfasta2phyml). Phylogenetic relationships were predicted using IQ‐TREE software v1.6.12 (Nguyen et al., 2015) with the LG + F + R10 amino acid substitution model, selected as the best fit by ModelFinder Plus (Kalyaanamoorthy et al., 2017) based on the Bayesian Information Criterion. Branch support for the phylogeny was assessed with 1000 ultrafast bootstrap replicates (Hoang et al., 2017). In addition to the sequence alignment, a constraint tree was provided as input in the IQ‐TREE analysis to achieve a topology of Arthropoda orders consistent with that described by Misof et al. (2014). The constraint tree was predicted using sequences from representative genomes of each order following the same steps described above, except for using RAxML software v.8.2.12 (Stamatakis, 2014) instead of IQ‐TREE. The resulting phylogenomic tree was rooted in the chelicerate species branch using iTOL (www.itol.embl.de), and the relationships among species were checked according to the phylogenies proposed in the articles listed in Supplementary Material 22. The proposed phylogenetic tree is presented in Supplementary Material 23.

### Identification of the orthologous groups containing the enzymes involved in the analysed pathways

Protein datasets annotated for each analysed genome were grouped into orthologous groups (OGs) using OrthoFinder (Emms & Kelly, 2019). OGs corresponding to each KEGG Orthology (KO) group involved in the analysed pathways were identified as follows.

The identification and the amino acid sequences of enzymes involved in the pathways of Arg biosynthesis (KEGG ID: map00220) and Arg and Pro metabolism (KEGG ID: map00330) were retrieved from the KO database. A BLASTp search (Altschul et al., 1990) was performed to identify similar sequences in the analysed genome, using all sequences associated with a specific KO in the KEGG database as queries and a maximum *e*‐value threshold of *e*‐5, and a minimum sequence identity of 70%. The OGs containing the sequences identified in the BLASTp search for each KO were determined and manually verified to prevent misidentification between KO and OG groups.

To estimate the number of genes in each OG that may encode functional enzymes, reference sequences with annotated active site residues were retrieved from the UNIPROT database. When available, these reference sequences were aligned with the corresponding OG sequences using MAFFT v7.503. This alignment was used to quantify how many sequences within each OG have the annotated active site residue at the same position as in the reference. Only the total number of genes per species genome was considered for enzymes lacking a described active site. A list of the analysed enzymes, their reference sequences, and the corresponding active site residues used for filtering is provided in Supplementary Material 24.

In cases where a gene was not identified in the genome annotation, the amino acid sequences associated with the corresponding KO in the KEGG database were aligned using MAFFT and subsequently used as queries to search for the gene in the genome sequences with the BITACORA software (Vizueta et al., 2020), utilizing genome mode and the proximity algorithm. All putative amino acid sequences identified through this analysis were validated using BLASTp against the NCBI non‐redundant database to detect potential misannotations or contamination from exogenous DNA. Furthermore, for genes with described active site residues, the amino acid sequences were carefully analysed to confirm the presence of these key residues.

### Gene expression analysis

Gene expression was examined using RNA sequencing (RNA‐seq) analysis. For this, the total RNA content for three biological samples from different tissues for the species *Ab. flavolineata* (Orthoptera), *P. americana* (Blattodea), *Ma. fimbriolata* (Hemiptera), *R. prolixus* (Hemiptera), *Dy. peruvianus* (Hemiptera), *T. molitor* (Coleoptera), *De. maculatus* (Coleoptera), *S. frugiperda* (Lepidoptera) and *Mu. domestica* (Diptera) was extracted and sequenced in an Illumina equipment. The insect‐rearing and RNA‐sequencing details can be found in Dias et al. (2019) and in Supplementary Material 25.

For the species with a genome available (*P. americana*, *R. prolixus*, *T. molitor*, *S. frugiperda* and *M. domestica*), RNA‐seq analysis was performed using the nf‐core/rna‐seq pipeline (Ewels et al., 2020). The STAR alignment strategy was used as a parameter for the pipeline (Dobin et al., 2013), with the reads being aligned against the respective genome sequences. The results of these analyses were measured under the RSEM quantification method (Li & Dewey, 2011), which describes the transcripts per million (TPM) values used as a standardized measure of the expression values per tissue (Wagner et al., 2012). The obtained TPM values for each biological triplicate were then used to calculate the average TPM value per tissue.

The transcriptome assembly and transcript expression analyses for *Ma. fimbriolata*, *Dy. peruvianus*, *Ab. flavolineata* and *De. maculatus* were retrieved from the analyses performed by Dias et al. (2018). For this species, the transcriptome protein sequences corresponding to each analysed enzyme were identified using a BLASTp search, using the corresponding OG sequences as queries. To verify the classification of these transcripts, when available, the active site residues described for the genome analysis were used (Supplementary Material 24). When active site residues were not annotated for the corresponding enzyme, the sequences were filtered based on the presence of the enzyme protein domains. For this filter, all OG sequences were submitted to InterProScan v.5.61‐93.0 (Jones et al., 2014) to identify the domains commonly associated with the corresponding enzymes. The transcriptome sequences containing at least one of the domains identified as present in at least 50% of the OG sequences were selected for the analysis. Finally, the sequences filtered by the presence of these domains were subjected to BLASTp searches against the UNIPROT database to remove any potential contamination from the dataset.

To establish the coherence of the dataset for *R. prolixus*, a transcriptome was also assembled for the species using Trinity v2.13.2 software (Grabherr et al., 2011). The coding sequences within the assembled transcript sequences were identified using Transdecoder v5.6.0 (https://github.com/TransDecoder/TransDecoder).

## AUTHOR CONTRIBUTIONS

**Jessica Cristina Silva Martins:** Methodology; writing – original draft; investigation; formal analysis; data curation. **Héctor Antônio Assunção Romão:** Investigation; writing – original draft; writing – review and editing; methodology; formal analysis; data curation; visualization. **Carolina Kurotusch Canettieri:** Investigation; methodology; formal analysis; data curation. **Amanda Caetano Cercilian:** Methodology; data curation; formal analysis. **Patrícia Rasteiro Ordiale Oliveira:** Data curation; formal analysis. **Clelia Ferreira:** Conceptualization; writing – review and editing; supervision; funding acquisition. **Walter R. Terra:** Conceptualization; funding acquisition; writing – review and editing; supervision. **Renata de Oliveira Dias:** Conceptualization; investigation; writing – original draft; writing – review and editing; supervision; project administration; visualization; resources.

## CONFLICT OF INTEREST STATEMENT

The authors declare no conflicts of interest.

## Supporting information


**Supplementary Material 1.** Number of genes identified in each genome.
**Supplementary Material 2**. Verification of missing genes in annotation using BITACORA.
**Supplementary Material 3**. Sequence alignment of all insect amino acid sequences classified as OTC in the KEGG Orthologous database, along with reference sequences.
**Supplementary Material 4**. Three‐dimensional structure alignment between *Homo sapiens* OTC and the predicted structure of the putative OTC from *Helicoverpa armigera*.
**Supplementary Material 5**. BLAST results for *Rhodnius prolixus* scaffold.
**Supplementary Material 6**. BLAST results for *Aphis craccivora* scaffold.
**Supplementary Material 7**. BLAST results for non‐Hemiptera ASS and ASL genes.
**Supplementary Material 8**. BLAST results for *Cimex* and *Apolygus* arginase genes.
**Supplementary Material 9**. Expression profile of genes analysed in *Spodoptera frugiperda*.
**Supplementary Material 10**. Expression profile of genes analysed in *Periplaneta americana*.
**Supplementary Material 11**. Expression profile of genes analysed in *Rhodnius prolixus*.
**Supplementary Material 12**. Expression profile of genes analysed in *Tenebrio molitor*.
**Supplementary Material 13**. Expression profile of genes analysed in *Musca domestica*.
**Supplementary Material 14**. Expression profile of genes analysed in *Abracris flavolineata*.
**Supplementary Material 15**. Expression profile of genes analysed in *Dermestes maculatus*.
**Supplementary Material 16**. Expression profile of genes analysed in *Dysdercus peruvianus*.
**Supplementary Material 17**. Expression profile of genes analysed in *Mahanarva fimbriolata*.
**Supplementary Material 18**. Sum of TPM values for genes encoding urea cycle enzymes in analysed insects.
**Supplementary Material 19**. Sum of TPM values for genes involved in ornithine biosynthesis and metabolism.
**Supplementary Material 20**. Sum of TPM values for genes involved in spermine biosynthesis and metabolism.
**Supplementary Material 21**. Summary of species analysed, including genome source and BUSCO performance.
**Supplementary Material 22**. List of articles used to assess phylogenetic relationships in the phylogenomic tree.
**Supplementary Material 23**. Proposed phylogenomic tree of Arthropoda.
**Supplementary Material 24**. Sequences and active site residues used to filter enzyme annotations.
**Supplementary Material 25**. Insect rearing protocols for RNA‐seq analysis.

## Data Availability

The data that support the findings of this study are openly available in NCBI at https://www.ncbi.nlm.nih.gov/, reference number provided in the Supplementary Materials.
