# Supplementary material for: The loss of the urea cycle and ornithine metabolism in different insect orders: An omics approach
Source: Insect Mol Biol. 2025 Mar 13;34(5):632–44. doi: 10.1111/imb.12989 (PMC12419136; doi:10.1111/imb.12989)
Supplement: Supplementary file 1 — Supplementary Material 1. Number of genes identified in each genome. Supplementary Material 2. Verification of missing genes in annotation using BITACORA. Supplementary Material 3. Sequence alignment of all insect amino acid sequences classified as OTC in the KEGG Orthologous database, along with reference sequences. Supplementary Material 4. Three‐dimensional structure alignment between Homo sapiens OTC and the predicted structure of the putative OTC from Helicoverpa armigera. Supplementary Material 5. BLAST results for Rhodnius prolixus scaffold. Supplementary Material 6. BLAST results for Aphis craccivora scaffold. Supplementary Material 7. BLAST results for non‐Hemiptera ASS and ASL genes. Supplementary Material 8. BLAST results for Cimex and Apolygus arginase genes. Supplementary Material 9. Expression profile of genes analysed in Spodoptera frugiperda. Supplementary Material 10. Expression profile of genes analysed in Periplaneta americana. Supplementary Material 11. Expression profile of genes analysed in Rhodnius prolixus. Supplementary Material 12. Expression profile of genes analysed in Tenebrio molitor. Supplementary Material 13. Expression profile of genes analysed in Musca domestica. Supplementary Material 14. Expression profile of genes analysed in Abracris flavolineata. Supplementary Material 15. Expression profile of genes analysed in Dermestes maculatus. Supplementary Material 16. Expression profile of genes analysed in Dysdercus peruvianus. Supplementary Material 17. Expression profile of genes analysed in Mahanarva fimbriolata. Supplementary Material 18. Sum of TPM values for genes encoding urea cycle enzymes in analysed insects. Supplementary Material 19. Sum of TPM values for genes involved in ornithine biosynthesis and metabolism. Supplementary Material 20. Sum of TPM values for genes involved in spermine biosynthesis and metabolism. Supplementary Material 21. Summary of species analysed, including genome source and BUSCO performance. Supplemen [file IMB-34-632-s001.docx]

**Supplementary Material**

**Supplementary Material 1** – Number of genes identified in each genome analysed.

| Species | ASS: T | ASL: T | ASL: F | NOS: T | ARG: T | OAT: T | P5CS: T | P5CDH: T | P5CDH: F | PYCR: T | PRODH: T | ODC: T | ODC: F | SRM: T | SRM: F | SMS: T | SMS: F | AMD: T | AMD: F |  |
| --- | --- | --- | --- | --- | --- | --- | --- | --- | --- | --- | --- | --- | --- | --- | --- | --- | --- | --- | --- | --- |
| Non-Insect genomes | | | | | | | | | | | | | | | | | | | | |
| *Tetranychus urticae* | 0 | 0 | 0 | 1 | 1 | 2 | 2 | 1 | 1 | 2 | 1 | 2 | 1 | 1 | 1 | 1 | 1 | 2 | 2 |  |
| *Dermatophagoides pteronyssinus* | 0 | 0 | 0 | 1 | 1 | 1 | 1 | 1 | 1 | 2 | 1 | 1 | 1 | 2 | 2 | 1 | 1 | 1 | 1 |  |
| *Varroa jacobsoni* | 0 | 0 | 0 | 0 | 2 | 2 | 1 | 1 | 1 | 2 | 1 | 4 | 4 | 1 | 1 | 1 | 1 | 1 | 1 |  |
| *Ixodes scapularis* | 1 | 1 | 1 | 1 | 7 | 3 | 1 | 1 | 1 | 2 | 3 | 3 | 1 | 1 | 1 | 1 | 1 | 1 | 1 |  |
| *Limulus polyphemus* | 1 | 1 | 1 | 6 | 3 | 1 | 1 | 1 | 1 | 2 | 5 | 9 | 4 | 2 | 2 | 1 | 1 | 1 | 1 |  |
| *Parasteatoda tepidariorum* | 1 | 1 | 1 | 3 | 2 | 1 | 2 | 1 | 1 | 3 | 3 | 4 | 4 | 1 | 1 | 1 | 1 | 1 | 1 |  |
| *Centruroides sculpturatus* | 1 | 1 | 1 | 4 | 2 | 1 | 1 | 1 | **1** | 2 | 3 | 7 | 4 | 1 | 1 | 1 | 1 | 1 | 1 |  |
| *Notodromas monacha* | 0 | 1 | 1 | 4 | 2 | 0 | 1 | 1 | 1 | 2 | 3 | 1 | 1 | 3 | 2 | 1 | 1 | 1 | 1 |  |
| *Darwinula stevensoni* | 3 | 1 | 1 | 9 | 2 | 2 | 1 | 1 | 1 | 1 | 1 | 1 | 1 | 1 | 1 | 1 | 1 | 1 | 0 |  |
| *Daphnia magna* | 1 | 1 | 1 | 3 | 1 | 1 | 1 | 1 | 1 | 2 | 2 | 1 | 1 | 1 | 1 | 1 | 1 | 1 | 1 |  |
| *Eurytemora affinis* | 0 | 0 | 0 | 0 | 2 | 1 | 1 | 1 | 1 | 2 | 5 | 1 | 0 | 0 | 0 | 1 | 1 | 1 | 1 |  |
| *Tigriopus californicus* | 0 | 0 | 0 | 1 | **1** | **1** | 1 | 1 | 1 | 2 | 2 | 2 | 1 | 0 | 0 | 4 | 2 | 1 | 1 |  |
| *Lepeophtheirus salmonis* | 0 | 0 | 0 | 0 | 1 | 1 | 1 | 1 | 1 | 3 | 1 | 0 | 0 | 0 | 0 | 2 | 2 | 1 | 1 |  |
| *Hyalella azteca* | 1 | 1 | 1 | 2 | 3 | 2 | 1 | 1 | 1 | 2 | 2 | 1 | 1 | 1 | 1 | 1 | 1 | 1 | 1 |  |
| *Penaeus japonicus* | 1 | 1 | 1 | 3 | 3 | 2 | 1 | 1 | 1 | 3 | 2 | 1 | 1 | 1 | 1 | 1 | 1 | 1 | 1 |  |
| *Portunus trituberculatus* | 1 | 1 | 1 | 3 | 3 | 1 | 1 | 1 | 1 | 3 | 2 | 1 | 1 | 2 | 2 | 1 | 1 | 1 | 1 |  |
| *Procambarus clarkii* | 1 | 1 | 1 | 4 | 4 | 1 | 1 | 1 | 1 | 2 | 2 | 2 | 2 | **1** | **1** | 1 | 1 | 1 | 1 |  |
| *Homarus americanus* | 1 | 1 | 1 | 1 | 3 | 1 | 1 | 1 | 1 | 2 | 2 | 1 | 1 | 1 | 1 | 2 | **1** | 1 | 1 |  |
| *Folsomia candida* | 0 | 0 | 0 | 1 | 8 | 1 | 1 | 1 | 1 | 1 | 3 | 2 | 2 | 1 | 1 | 2 | 2 | 1 | 1 |  |
| *Catajapyx aquilonaris* | 0 | 0 | 0 | 2 | 1 | 1 | 1 | 1 | 1 | 2 | 2 | 3 | 1 | 1 | 1 | 1 | **1** | 1 | 1 |  |
| Odonata | | | | | | | | | | | | | | | | | | | | |
| *Ischnura elegans* | 0 | 0 | 0 | 1 | 1 | 1 | 1 | 1 | 1 | 2 | 2 | 1 | 1 | 1 | 1 | 1 | 1 | 1 | 1 |  |
| Orthoptera | | | | | | | | | | | | | | | | | | | | |
| *Schistocerca americana* | 1 | 1 | 1 | 2 | 1 | 1 | 1 | 1 | 1 | 2 | 2 | 8 | 8 | 1 | 1 | 2 | 2 | 1 | 1 |  |
| Blattodea | | | | | | | | | | | | | | | | | | | | |
| *Blattella germanica* | 1 | 1 | 1 | 2 | 2 | 1 | 1 | 1 | 1 | 3 | 4 | 2 | 2 | 1 | 1 | 1 | **1** | 1 | 0 |  |
| *Periplaneta americana* | 2 | 5 | 4 | 2 | 2 | 1 | 2 | 1 | 1 | 2 | 1 | 3 | 3 | 2 | 2 | 1 | 1 | 1 | 1 |  |
| *Zootermopsis nevadensis* | 1 | 1 | 1 | 1 | 1 | 1 | 1 | 1 | 1 | 2 | 2 | 1 | 1 | 1 | 1 | 1 | 1 | 1 | 1 |  |
| *Cryptotermes secundus* | 1 | 1 | 1 | 1 | 1 | 1 | 1 | 1 | 1 | 2 | 2 | 1 | 1 | 1 | 1 | 1 | 1 | 1 | 1 |  |
| *Coptotermes formosanus* | 1 | 1 | 1 | 1 | 1 | 1 | 1 | 1 | 1 | 2 | 3 | 1 | 1 | 1 | 1 | 1 | 1 | 1 | 1 |  |
| Thysanoptera | | | | | | | | | | | | | | | | | | | | |
| *Thrips palmi* | 0 | 0 | 0 | 1 | 1 | 1 | 1 | 1 | 1 | 2 | 1 | 2 | 2 | 1 | 1 | 1 | 1 | 1 | 1 |  |
| *Frankliniella occidentalis* | 0 | 0 | 0 | 1 | 1 | **1** | 1 | 1 | 1 | 2 | 1 | 3 | 2 | 1 | 1 | 1 | 1 | 1 | 1 |  |
| Hemiptera | | | | | | | | | | | | | | | | | | | | |
| *Nilaparvata lugens* | **0** | **0** | **0** | 2 | 0 | 2 | 1 | 1 | 1 | 2 | 2 | 1 | 1 | 1 | 1 | 2 | 2 | 1 | 1 |  |
| *Laodelphax striatellus* | 0 | 0 | 0 | 1 | 0 | 2 | 1 | 1 | 1 | 2 | 2 | 1 | 1 | 2 | 1 | 2 | 2 | 1 | 1 |  |
| *Halyomorpha halys* | 0 | 0 | 0 | 1 | 0 | 2 | 1 | 1 | 1 | 2 | 2 | 2 | 1 | 1 | 1 | 1 | 1 | 1 | 1 |  |
| *Rhodnius prolixus* | **0** | 0 | 0 | 2 | 0 | 2 | 1 | 1 | **1** | 3 | 3 | 1 | **1** | 1 | 1 | 2 | 1 | 1 | 1 |  |
| *Cimex lectularius* | 0 | 0 | 0 | 1 | 1 | 4 | 1 | 1 | 1 | 2 | 2 | 2 | 1 | 1 | 1 | 1 | 1 | 1 | 1 |  |
| *Apolygus lucorum* | 0 | 0 | 0 | 1 | 1 | 2 | 1 | 1 | 1 | 3 | 2 | 2 | 1 | 1 | 1 | 1 | 1 | 1 | 1 |  |
| *Bemisia tabaci* | 0 | 0 | 0 | 1 | 0 | 3 | 1 | 1 | 1 | 4 | 1 | 4 | 4 | 1 | 1 | 1 | 1 | 1 | 1 |  |
| *Daktulosphaira vitifoliae* | 0 | 0 | 0 | 1 | 0 | 1 | 1 | 1 | 1 | 1 | 2 | 2 | 1 | 2 | 2 | 0 | 0 | 1 | 1 |  |
| *Eriosoma lanigerum* | 0 | 0 | 0 | 1 | 0 | 1 | 1 | 1 | 1 | 2 | 1 | 3 | 1 | 2 | 2 | 0 | 0 | 1 | 1 |  |
| *Sipha flava* | 0 | 0 | 0 | 1 | 0 | 1 | 1 | 1 | 1 | 2 | 1 | 1 | 1 | 2 | 2 | 0 | 0 | 1 | 1 |  |
| *Aphis craccivora* | 0 | **0** | **0** | 2 | 0 | 1 | 1 | 1 | 1 | 2 | 2 | 2 | 2 | 5 | 4 | 0 | 0 | 3 | 0 |  |
| *Rhopalosiphum maidis* | 0 | 0 | 0 | 1 | 0 | 1 | 1 | 1 | 1 | 2 | 1 | 1 | 1 | 2 | 2 | 0 | 0 | 1 | 1 |  |
| *Melanaphis sacchari* | 0 | 0 | 0 | 1 | 0 | 1 | 1 | 1 | 1 | 2 | 1 | 2 | 1 | 2 | 2 | 0 | 0 | 1 | 1 |  |
| *Diuraphis noxia* | 0 | 0 | 0 | 1 | 0 | 1 | 1 | 1 | 1 | 2 | 1 | 3 | 3 | 3 | 3 | 0 | 0 | 1 | 1 |  |
| *Myzus persicae* | 0 | 0 | 0 | 1 | 0 | 2 | 1 | 1 | 1 | 2 | 1 | 4 | 3 | 3 | 3 | 0 | 0 | 1 | 1 |  |
| *Acyrthosiphon pisum* | 0 | **0** | **0** | 1 | 0 | 1 | 1 | 1 | 1 | 3 | 1 | 2 | 2 | 3 | 3 | 0 | 0 | 1 | 1 |  |
| Phthiraptera | | | | | | | | | | | | | | | | | | | | |
| *Pediculus humanus corporis* | 0 | 0 | 0 | 1 | 1 | 2 | 1 | 1 | 1 | 2 | 2 | 1 | 1 | 1 | 1 | 1 | 1 | 1 | 1 |  |
| Hymenoptera | | | | | | | | | | | | | | | | | | | | |
| *Athalia rosae* | 0 | 0 | 0 | 1 | 1 | 1 | 1 | 1 | 1 | 2 | 1 | 4 | 2 | 1 | 1 | 1 | 1 | 1 | 1 |  |
| *Neodiprion lecontei* | 0 | 0 | 0 | 1 | 1 | 1 | 1 | 1 | 1 | 3 | 1 | 4 | 4 | 1 | 1 | 1 | 1 | 1 | 1 |  |
| *Diprion similis* | 0 | 0 | 0 | 1 | 1 | 2 | 1 | 1 | 1 | 3 | 1 | 3 | 2 | 1 | 1 | 1 | 1 | 0 | 0 |  |
| *Cephus cinctus* | 0 | 0 | 0 | 1 | 1 | 2 | 1 | 1 | 1 | 3 | 1 | 2 | 2 | 1 | 1 | 1 | 1 | 1 | 1 |  |
| *Orussus abietinus* | 1 | 1 | 1 | 1 | 1 | 2 | 1 | 1 | 1 | 3 | 2 | 2 | 2 | 1 | 1 | 1 | 1 | 1 | 1 |  |
| *Leptopilina heterotoma* | 1 | 1 | 1 | 1 | **1** | 2 | 1 | 1 | 1 | 2 | 1 | 3 | 2 | 1 | 1 | 1 | 1 | 1 | 1 |  |
| *Belonocnema treatae* | **1** | 0 | 0 | 1 | 1 | 2 | 1 | 1 | 1 | 2 | 1 | 4 | 2 | 1 | 1 | 1 | 1 | 1 | 1 |  |
| *Trichogramma pretiosum* | 0 | 0 | 0 | 3 | 1 | 2 | 1 | 1 | 1 | 2 | 1 | 2 | 2 | 1 | 1 | 1 | 1 | 1 | 1 |  |
| *Copidosoma floridanum* | 0 | 0 | 0 | 2 | 1 | 2 | 1 | 1 | 1 | 2 | 1 | 4 | 3 | 2 | 2 | 3 | 3 | 1 | 1 |  |
| *Nasonia vitripennis* | 1 | 1 | 1 | 1 | 1 | 2 | 1 | 1 | 1 | 2 | 1 | 4 | 4 | 1 | 1 | 1 | 1 | 1 | 1 |  |
| *Ceratosolen solmsi marchali* | 0 | 1 | 1 | 1 | 1 | 2 | 1 | 1 | 1 | 2 | 1 | 2 | 2 | 1 | 1 | 1 | 1 | 1 | 1 |  |
| *Venturia canescens* | 1 | 1 | 1 | 1 | 1 | 2 | 1 | 1 | 1 | 2 | 1 | 2 | 2 | 1 | 1 | 1 | 1 | 1 | 1 |  |
| *Diachasma alloeum* | 0 | 0 | 0 | 1 | 1 | 2 | 1 | 1 | 1 | 2 | 1 | 2 | 2 | 1 | 1 | 1 | 1 | 1 | 1 |  |
| *Fopius arisanus* | 0 | 0 | 0 | 1 | 1 | 1 | 1 | 1 | 1 | 2 | 1 | 2 | 2 | 1 | 1 | 1 | 1 | 1 | 1 |  |
| *Aphidius gifuensis* | 0 | 0 | 0 | 1 | 1 | 2 | 1 | 1 | 1 | 2 | 1 | 4 | 3 | 1 | 1 | 1 | 1 | 1 | 1 |  |
| *Chelonus insularis* | 0 | 0 | 0 | 1 | 1 | 1 | 1 | 1 | 1 | 2 | 1 | 1 | 1 | 1 | 1 | 1 | 1 | 1 | 1 |  |
| *Microplitis demolitor* | 0 | 0 | 0 | 1 | 1 | 1 | 1 | 1 | 1 | 2 | 1 | 2 | 2 | 1 | 1 | 1 | 1 | 1 | 1 |  |
| *Cotesia glomerata* | 0 | 0 | 0 | 1 | 1 | 2 | 1 | 1 | 1 | 2 | 1 | 2 | 2 | 1 | 1 | 1 | 1 | 1 | 1 |  |
| *Ampulex compressa* | 1 | 1 | 1 | 1 | **1** | 2 | 1 | 1 | 1 | 2 | 1 | 2 | 2 | 1 | 1 | 1 | 1 | 1 | 1 |  |
| *Colletes gigas* | 0 | 0 | 0 | 1 | 1 | 2 | 1 | 1 | 1 | 2 | 1 | 2 | 2 | 1 | 1 | 1 | 1 | 1 | 1 |  |
| *Dufourea novaeangliae* | **1** | 0 | 0 | 1 | 1 | 2 | 1 | 1 | 1 | 2 | 1 | 2 | 2 | 2 | 2 | 1 | 1 | 1 | 1 |  |
| *Nomia melanderi* | 1 | 1 | 1 | 1 | 1 | 2 | 1 | 1 | 1 | 2 | 1 | 2 | 2 | 1 | 1 | 1 | 1 | 1 | 1 |  |
| *Megalopta genalis* | 1 | 2 | 2 | 2 | 1 | 2 | 1 | 1 | 1 | 2 | 1 | 2 | 2 | 1 | 1 | 1 | 1 | 1 | 1 |  |
| *Osmia lignaria* | 0 | 0 | 0 | 0 | 1 | 2 | 1 | 1 | 1 | 2 | 1 | 2 | 2 | 1 | 1 | 1 | 1 | 1 | 1 |  |
| *Megachile rotundata* | 0 | 0 | 0 | 1 | 1 | 1 | 1 | 1 | 1 | 2 | 1 | 2 | 2 | 1 | 1 | 1 | 1 | 1 | 1 |  |
| *Habropoda laboriosa* | 1 | 1 | 1 | 1 | 1 | 2 | 1 | 1 | 1 | 2 | 1 | 2 | 2 | 1 | 1 | 1 | 1 | 1 | 1 |  |
| *Ceratina calcarata* | 0 | 0 | 0 | 1 | 1 | 2 | 1 | 1 | 1 | 2 | 1 | 3 | 3 | 1 | 1 | 2 | 1 | 1 | 1 |  |
| *Eufriesea mexicana* | 1 | 1 | 1 | 1 | 1 | 1 | 1 | 1 | 1 | 3 | 1 | 3 | 3 | 1 | 1 | 1 | 1 | 1 | 1 |  |
| *Apis laboriosa* | 1 | 1 | 1 | 1 | 2 | 2 | 1 | 1 | 1 | 2 | 1 | 1 | 1 | 1 | 1 | 1 | 1 | 1 | 1 |  |
| *Bombus terrestris* | 1 | 0 | 0 | 1 | 1 | 2 | 1 | 1 | 1 | 2 | 1 | 3 | 2 | 1 | 1 | 1 | 1 | 1 | 1 |  |
| *Melipona quadrifasciata* | 1 | 1 | 1 | 1 | 1 | 2 | **1** | 1 | 1 | 1 | **1** | 2 | 2 | 1 | 1 | 1 | 1 | 1 | 0 |  |
| *Frieseomelitta varia* | 1 | 1 | 1 | 1 | 1 | 1 | 1 | 1 | 1 | 2 | 1 | 2 | 2 | 1 | 1 | 2 | 1 | 1 | 0 |  |
| *Polistes canadensis* | 1 | 1 | 1 | 1 | 1 | 2 | 1 | 1 | 1 | 2 | 1 | 3 | 3 | 1 | 1 | 1 | 1 | 1 | 1 |  |
| *Vespula pensylvanica* | 1 | 1 | 1 | 1 | 1 | 2 | 1 | 1 | 1 | 2 | 1 | 8 | 6 | 1 | 1 | 1 | 1 | 1 | 1 |  |
| *Vespa crabro* | 1 | 1 | 1 | 1 | 1 | 2 | 1 | 1 | 1 | 2 | 1 | 8 | 7 | 1 | 1 | 1 | 1 | 1 | 1 |  |
| *Dinoponera quadriceps* | 1 | 1 | 1 | 1 | 1 | 1 | 1 | 1 | 1 | 2 | 3 | 5 | 5 | 1 | 1 | 1 | 1 | 1 | 1 |  |
| *Odontomachus brunneus* | 1 | 1 | 1 | 1 | 1 | 2 | 1 | 1 | 1 | 2 | 1 | 5 | 2 | 1 | 1 | 1 | 1 | 1 | 1 |  |
| *Harpegnathos saltator* | 1 | 1 | 1 | 1 | 1 | 2 | 1 | 1 | 1 | 2 | 2 | 5 | 2 | 1 | 1 | 1 | 1 | 1 | 1 |  |
| *Ooceraea biroi* | 0 | 0 | 0 | 1 | 1 | 2 | 1 | 1 | 1 | 2 | 1 | 4 | 2 | 1 | 1 | 1 | 1 | 1 | 1 |  |
| *Eciton burchellii* | 1 | 0 | 0 | 1 | 1 | 2 | 1 | 1 | 1 | 2 | 1 | 3 | 2 | 1 | 1 | 1 | 1 | 1 | 1 |  |
| *Pseudomyrmex gracilis* | 1 | 0 | 0 | 1 | 1 | 2 | 1 | 1 | 1 | 2 | 1 | 3 | 2 | 1 | 1 | 1 | 1 | 1 | 1 |  |
| *Linepithema humile* | 1 | 1 | 1 | 1 | 1 | 1 | 1 | 1 | 1 | 2 | 1 | 4 | 4 | 1 | 1 | 1 | 1 | 1 | 1 |  |
| *Nylanderia fulva* | 1 | 1 | 1 | 1 | 1 | 2 | 1 | 1 | 1 | 2 | 1 | 1 | 0 | 1 | 1 | 1 | 1 | 1 | 1 |  |
| *Formica exsecta* | 1 | **1** | **1** | 1 | 1 | 2 | 1 | 1 | 1 | 2 | 1 | 5 | 3 | 1 | 1 | 1 | 1 | 1 | 1 |  |
| *Camponotus floridanus* | 1 | 1 | 1 | 1 | 1 | 2 | 1 | 1 | 1 | 2 | 1 | 3 | 2 | 1 | 1 | 1 | 1 | 1 | 1 |  |
| *Pogonomyrmex barbatus* | 1 | 1 | 0 | 1 | 1 | 1 | 1 | 1 | 1 | 2 | 1 | 5 | 2 | 1 | 1 | 1 | 1 | 1 | 1 |  |
| *Vollenhovia emeryi* | 1 | 1 | 1 | 1 | 1 | 1 | 1 | 1 | 1 | 2 | 1 | 4 | 4 | 1 | 1 | 1 | 1 | 1 | 1 |  |
| *Solenopsis invicta* | 1 | 1 | 1 | 1 | 1 | 5 | 1 | 1 | 1 | 2 | 1 | 4 | 2 | 1 | 1 | 1 | 1 | 1 | 1 |  |
| *Monomorium pharaonis* | 1 | 0 | 0 | 1 | 1 | 4 | 1 | 1 | 1 | 2 | 1 | 4 | 2 | 1 | 1 | 1 | 1 | 1 | 1 |  |
| *Temnothorax longispinosus* | **1** | 1 | 0 | 1 | **1** | 1 | 1 | 1 | 1 | 2 | 1 | 4 | 2 | 1 | 1 | **1** | **1** | 1 | 1 |  |
| *Wasmannia auropunctata* | 1 | 1 | 1 | 1 | 1 | 1 | 1 | 1 | 1 | 2 | 1 | 4 | 4 | 1 | 1 | 1 | 1 | 1 | 1 |  |
| *Cyphomyrmex costatus* | 0 | 0 | 0 | 1 | 1 | 1 | 1 | 1 | 1 | 2 | 1 | 6 | 6 | 1 | 1 | 1 | 1 | 1 | 1 |  |
| *Trachymyrmex septentrionalis* | 1 | 0 | 0 | 1 | 1 | 1 | 1 | 1 | 1 | 3 | 1 | 3 | 3 | 1 | 1 | 1 | 1 | 1 | 1 |  |
| *Atta colombica* | 1 | 0 | 0 | 1 | 1 | 1 | 1 | 1 | 1 | 2 | 1 | 3 | 3 | 1 | 1 | 1 | 1 | 1 | 1 |  |
| *Pseudoatta argentina* | **1** | 0 | 0 | 1 | 1 | 1 | 1 | 1 | 1 | 2 | 1 | 2 | 1 | 1 | 1 | 1 | 1 | 1 | 1 |  |
| *Acromyrmex echinatior* | 1 | 0 | 0 | 1 | 1 | 1 | 1 | 1 | 1 | 2 | 1 | 3 | 3 | 1 | 1 | 1 | 1 | 1 | 1 |  |
| Neuroptera | | | | | | | | | | | | | | | | | | | | |
| *Chrysoperla carnea* | 1 | 1 | 1 | 1 | 1 | 2 | 1 | 2 | 2 | 2 | 1 | 9 | 6 | 1 | 1 | 1 | 1 | 1 | 1 |  |
| Coleoptera | | | | | | | | | | | | | | | | | | | | |
| *Agrilus planipennis* | 0 | 0 | 0 | 1 | 1 | 2 | 1 | 2 | 2 | 3 | 4 | 1 | 1 | 1 | 1 | 1 | 1 | 1 | 1 |  |
| *Ignelater luminosus* | 1 | 1 | 1 | 2 | 1 | 2 | 1 | 1 | 1 | 3 | 1 | 3 | 3 | 1 | 1 | 1 | 1 | 1 | 1 |  |
| *Photinus pyralis* | 1 | 1 | 1 | 3 | 2 | 5 | 1 | 1 | 1 | 2 | 1 | 7 | 5 | 1 | 1 | 1 | 1 | 1 | 1 |  |
| *Lamprigera yunnana* | 2 | 2 | 2 | 1 | 1 | 2 | 1 | 1 | 1 | 2 | 1 | 5 | 4 | 1 | 1 | 1 | 1 | 1 | 1 |  |
| *Abscondita terminalis* | 2 | 3 | 3 | 1 | 6 | 5 | 1 | 1 | 1 | 1 | 1 | 4 | 3 | 1 | 1 | 1 | 1 | 1 | 1 |  |
| *Onthophagus taurus* | 1 | 1 | 1 | 1 | 2 | 6 | 1 | 1 | 1 | 3 | 1 | 1 | 1 | 1 | 1 | 1 | 1 | 1 | 1 |  |
| *Nicrophorus vespilloides* | 1 | 1 | 1 | 2 | 1 | 3 | 1 | 1 | 1 | 5 | 1 | 1 | 1 | 1 | 1 | 1 | 1 | 1 | 1 |  |
| *Coccinella septempunctata* | 0 | 0 | 0 | 1 | 0 | 1 | 1 | 1 | 1 | 2 | 1 | 1 | 1 | 1 | 1 | 1 | 1 | 1 | 1 |  |
| *Tribolium madens* | 1 | 1 | 1 | 1 | 1 | 1 | 1 | 1 | 1 | 3 | 2 | 1 | 1 | 1 | 1 | 1 | 1 | 1 | 1 |  |
| *Tenebrio molitor* | 1 | 1 | 1 | 1 | 1 | 1 | 1 | 1 | 1 | 2 | 1 | 1 | 1 | 1 | 1 | 1 | 1 | 1 | 1 |  |
| *Brassicogethes aeneus* | 0 | 0 | 0 | 1 | 1 | 1 | 1 | 1 | 1 | 4 | 1 | 3 | 3 | 1 | 1 | 1 | 1 | 2 | 2 |  |
| *Aethina tumida* | 1 | 1 | 1 | 2 | 1 | 1 | 1 | 2 | 2 | 4 | 1 | 1 | 1 | 1 | 1 | 1 | 1 | 1 | 1 |  |
| *Dendroctonus ponderosae* | 1 | 1 | 1 | 1 | 1 | 1 | 2 | 1 | 1 | 3 | 1 | 1 | 1 | 1 | 1 | 1 | 1 | 1 | 1 |  |
| *Sitophilus oryzae* | 1 | 1 | 1 | 2 | 1 | 1 | 2 | 1 | 1 | 3 | 1 | 1 | 1 | 1 | 1 | 1 | 1 | 1 | 1 |  |
| *Rhynchophorus ferrugineus* | 2 | 1 | 1 | 1 | 1 | 1 | 2 | 2 | 2 | 3 | 1 | 6 | 1 | 1 | 1 | 1 | 1 | 1 | 0 |  |
| *Anoplophora glabripennis* | 1 | 1 | 1 | 1 | 1 | 1 | 2 | 1 | 1 | 3 | 2 | 2 | 2 | 1 | 1 | 1 | 1 | 1 | 1 |  |
| *Diabrotica virgifera virgifera* | 0 | 0 | 0 | 3 | 1 | 2 | 1 | 1 | 1 | 2 | 2 | 5 | 3 | 1 | 1 | 0 | 0 | 1 | 1 |  |
| *Leptinotarsa decemlineata* | 1 | 1 | 1 | 1 | 1 | 2 | 1 | 1 | 1 | 3 | 1 | 2 | 2 | 1 | 1 | 2 | 1 | 1 | 1 |  |
| *Gonioctena quinquepunctata* | 1 | 1 | 1 | 2 | 2 | 4 | 1 | 1 | 1 | 3 | 1 | 1 | 1 | 1 | 1 | 0 | 0 | 1 | 1 |  |
| Diptera | | | | | | | | | | | | | | | | | | | | |
| *Polypedilum vanderplanki* | 1 | 1 | 1 | 1 | 1 | 1 | 1 | 1 | 1 | 2 | 1 | 9 | 5 | 1 | 1 | 1 | 1 | 1 | 1 |  |
| *Clunio marinus* | 1 | 1 | 1 | 2 | 1 | 1 | 1 | 1 | 1 | 2 | 1 | 3 | 3 | 1 | 1 | 1 | 1 | 1 | 1 |  |
| *Anopheles albimanus* | 1 | 1 | 1 | 1 | 1 | 1 | 1 | 1 | 1 | 2 | 1 | 7 | 7 | 1 | 1 | 1 | 1 | 1 | 1 |  |
| *Aedes aegypti* | 1 | 1 | 1 | 1 | 1 | 1 | 1 | 1 | 1 | 3 | 1 | 6 | 5 | 2 | 2 | 1 | 1 | 1 | 0 |  |
| *Culex quinquefasciatus* | 1 | 1 | 1 | 1 | 1 | 2 | 1 | 1 | 1 | 2 | 1 | 9 | 9 | 1 | 1 | 2 | 2 | 1 | 1 |  |
| *Culex pipiens pallens* | 1 | 1 | 1 | 1 | 1 | 2 | 1 | 1 | 1 | 3 | 1 | 7 | 5 | 1 | 1 | 1 | 1 | 1 | 1 |  |
| *Contarinia nasturtii* | 0 | 0 | 0 | 1 | 1 | 1 | 1 | 1 | 1 | 2 | 1 | 2 | 1 | 1 | 1 | 2 | 2 | 1 | 1 |  |
| *Bradysia coprophila* | 0 | 1 | 1 | 3 | 1 | 1 | 1 | 3 | 3 | 2 | 1 | 3 | 3 | 1 | 1 | 1 | 1 | 1 | 1 |  |
| *Hermetia illucens* | 1 | 1 | 1 | 1 | 1 | 1 | 1 | 2 | 2 | 3 | 1 | 2 | 2 | 2 | 2 | 1 | 1 | 1 | 1 |  |
| *Ceratitis capitata* | 1 | 1 | 1 | 2 | 1 | 1 | 1 | 2 | 1 | 2 | 1 | 1 | 1 | 1 | 1 | 1 | 1 | 1 | 1 |  |
| *Zeugodacus cucurbitae* | 1 | 1 | 1 | 1 | **1** | 1 | 1 | 2 | 2 | 2 | 1 | 1 | 1 | 1 | 1 | 1 | 1 | 1 | 1 |  |
| *Bactrocera latifrons* | 1 | 1 | 1 | 2 | 1 | 1 | 1 | 1 | 1 | 2 | 1 | 1 | 1 | 1 | 1 | 1 | 1 | 1 | 1 |  |
| *Glossina fuscipes* | 0 | 1 | 1 | 1 | 1 | 1 | 1 | 2 | 2 | 2 | 1 | 2 | 2 | 1 | 1 | 1 | 1 | 1 | 1 |  |
| *Lucilia cuprina* | 1 | 1 | 1 | 1 | 1 | 1 | 1 | 3 | 3 | 3 | 1 | 2 | 2 | 1 | 1 | 1 | 1 | 1 | 1 |  |
| *Stomoxys calcitrans* | 1 | 1 | 1 | 1 | 1 | 1 | 2 | 3 | 3 | 2 | 1 | 3 | 3 | 1 | 1 | 1 | 1 | 1 | 1 |  |
| *Musca domestica* | 2 | 1 | 1 | 2 | 1 | 1 | 2 | 2 | 2 | 2 | 1 | 3 | 3 | 1 | 1 | 1 | 1 | 1 | 1 |  |
| *Scaptodrosophila lebanonensis* | 1 | 1 | 1 | 1 | 1 | 1 | 1 | 3 | 2 | 2 | 1 | 2 | 2 | 1 | 1 | 1 | 1 | 1 | 1 |  |
| *Zaprionus bogoriensis* | 1 | 1 | 1 | 2 | 1 | 1 | 1 | 2 | 1 | 2 | 1 | 2 | 2 | 1 | 1 | 1 | 1 | 1 | 1 |  |
| *Drosophila virilis* | 1 | 1 | 1 | 1 | 1 | 1 | 1 | 2 | 1 | 2 | 1 | 1 | 1 | 2 | 2 | 1 | 1 | 1 | 1 |  |
| *Drosophila takahashii* | 1 | 1 | 1 | 1 | 1 | 1 | 1 | 3 | 2 | 2 | 1 | 2 | 2 | 1 | 1 | 1 | 1 | 1 | 1 |  |
| *Drosophila melanogaster* | 1 | 1 | 1 | 1 | 1 | 1 | 1 | 3 | 2 | 2 | 1 | 2 | 2 | 1 | 1 | 1 | 1 | 1 | 1 |  |
| *Drosophila pseudoobscura* | 1 | 1 | 1 | 1 | 1 | 1 | 1 | 2 | 1 | 4 | 1 | 1 | 1 | 1 | 1 | 1 | 1 | 1 | 1 |  |
| *Drosophila guanche* | 1 | 1 | 1 | 1 | 1 | 1 | 1 | 3 | 2 | 2 | 1 | 1 | 1 | 1 | 1 | 1 | 1 | 1 | 1 |  |
| Lepidoptera | | | | | | | | | | | | | | | | | | | | |
| *Plutella xylostella* | 1 | 1 | 1 | 2 | 1 | 1 | 1 | 1 | 1 | 2 | 1 | 3 | 2 | 1 | 1 | 1 | 1 | 1 | 1 |  |
| *Parnassius apollo* | 1 | 1 | 1 | 2 | 1 | 1 | 1 | 1 | 1 | 2 | 1 | 2 | 2 | 1 | 1 | 1 | 1 | 1 | 1 |  |
| *Papilio xuthus* | 1 | 1 | 1 | 2 | 1 | 1 | 1 | 1 | 1 | 2 | 1 | 2 | 2 | 1 | 1 | 1 | 1 | 1 | 1 |  |
| *Pieris brassicae* | 1 | 1 | 1 | 2 | 1 | 1 | 1 | 1 | 1 | 2 | 1 | 2 | 1 | 1 | 1 | 1 | 1 | 1 | 1 |  |
| *Zerene cesonia* | 1 | 1 | 1 | 2 | 1 | 1 | 1 | 1 | 1 | 2 | 2 | 2 | 0 | 1 | 1 | 1 | 1 | 1 | 1 |  |
| *Colias croceus* | 1 | 1 | 1 | 2 | 1 | 1 | 1 | 1 | 1 | 2 | 1 | 2 | 1 | 1 | 1 | 1 | 1 | 1 | 1 |  |
| *Aricia agestis* | 1 | 1 | 1 | 2 | 1 | 1 | 1 | 1 | 1 | 2 | 1 | 1 | 1 | 1 | 1 | 1 | 1 | 1 | 1 |  |
| *Danaus plexippus plexippus* | 1 | 1 | 1 | 2 | 1 | 1 | 1 | 1 | 1 | 2 | 1 | 5 | 3 | 1 | 1 | 1 | 1 | 1 | 1 |  |
| *Vanessa atalanta* | 1 | 1 | 1 | 2 | 1 | 1 | 1 | 1 | 1 | 2 | 1 | 2 | 2 | 1 | 1 | 1 | 1 | 1 | 1 |  |
| *Brenthis ino* | 1 | 1 | 1 | 2 | 1 | 1 | 1 | 1 | 1 | 2 | 1 | 3 | 2 | 1 | 1 | 1 | 1 | 1 | 1 |  |
| *Maniola jurtina* | 1 | 1 | 1 | 2 | 1 | 1 | 1 | 1 | 1 | 2 | 1 | 3 | 3 | 1 | 1 | 1 | 1 | 1 | 1 |  |
| *Pararge aegeria* | 1 | 1 | 1 | 2 | 1 | 1 | 1 | 1 | 1 | 3 | 1 | 2 | 2 | 1 | 1 | 1 | 1 | 1 | 1 |  |
| *Bicyclus anynana* | 1 | 1 | 1 | 2 | 1 | 1 | 1 | 1 | 1 | 2 | 1 | 3 | 2 | 1 | 1 | 1 | 1 | 1 | 1 |  |
| *Hyposmocoma kahamanoa* | 1 | 1 | 1 | 3 | 1 | 1 | 1 | 1 | 1 | 2 | 1 | 2 | 2 | 1 | 1 | 1 | 1 | 1 | 0 |  |
| *Ostrinia furnacalis* | 1 | 1 | 1 | 2 | 1 | 1 | 1 | 1 | 1 | 2 | 1 | 1 | 1 | 1 | 1 | 1 | 1 | 1 | 1 |  |
| *Chilo suppressalis* | 2 | 1 | 1 | 2 | 1 | 1 | 1 | **1** | **1** | 2 | 1 | 2 | 2 | 1 | 1 | 1 | 1 | 1 | 1 |  |
| *Galleria mellonella* | 1 | 1 | 1 | 2 | 1 | 1 | 1 | 1 | 1 | 2 | 1 | 1 | 1 | 1 | 1 | 1 | 1 | 1 | 1 |  |
| *Amyelois transitella* | 1 | 1 | 1 | 3 | 1 | 1 | 1 | 1 | 1 | 3 | 1 | 2 | 2 | 1 | 1 | 1 | 1 | 1 | 1 |  |
| *Manduca sexta* | 1 | 1 | 1 | 2 | 1 | 1 | 1 | 1 | 1 | 3 | 2 | 5 | 4 | 1 | 1 | 1 | 1 | 1 | 1 |  |
| *Bombyx mori* | 1 | 1 | 1 | 2 | 1 | 1 | 1 | 1 | 1 | 2 | 1 | 1 | 1 | 1 | 1 | 1 | 1 | 1 | 1 |  |
| *Arctia plantaginis* | 1 | 1 | 1 | 3 | 1 | 3 | 1 | 1 | 1 | 2 | 1 | 1 | 1 | 1 | 1 | 1 | 1 | 2 | 1 |  |
| *Trichoplusia ni* | 1 | 1 | 1 | 3 | 1 | 1 | 1 | 3 | 2 | 2 | 1 | 3 | 2 | 1 | 1 | 1 | 1 | 1 | 1 |  |
| *Helicoverpa zea* | 1 | 1 | 1 | 2 | 1 | 1 | 1 | 2 | 1 | 2 | 1 | 1 | 1 | 1 | 1 | 1 | 1 | 1 | 1 |  |
| *Spodoptera litura* | 1 | 2 | 2 | 2 | 1 | 2 | 1 | 3 | 2 | 2 | 1 | 1 | 1 | 1 | 1 | 2 | 1 | 1 | 1 |  |
| *Spodoptera frugiperda* | 1 | 1 | 1 | 2 | 1 | 1 | 1 | 4 | 2 | 2 | 2 | 1 | 1 | 1 | 1 | 1 | 1 | 1 | 1 |  |

*The species are shown in the same order as they appear on the taxonomic tree in the Supplementary Material 3.

**For genes with active residues identified, a second column (shaded in red) was added containing the number of genes containing the respective residues in the same alignment position as the reference sequence (F).

***All columns were shaded to highlight the genomes with a more significant number of genes for each analyzed protein.

**** Numbers highlighted in yellow represent genes predicted by BITACORA, while those highlighted in green were removed from the annotations as potential misannotations, as detailed in the manuscript.

**Supplementary Material 2 -** Verification of missing genes in annotation using BITACORA

| **Species** | **Gene** | **Found using BITACORA** | **Sequence** |
| --- | --- | --- | --- |
| *Tetranychus urticae* | ASS | No |  |
|  | ASL | No |  |
| *Dermatophagoides pteronyssinus* | ASS | No |  |
|  | ASL | No |  |
| *Varroa jacobsoni* | ASS | No |  |
|  | ASL | No |  |
|  | NOS | No |  |
| *Centruroides sculpturatus* | P5CDH | Yes | >NW_019384643.1_exon9_exon7_exon5_exon8_exon6_exon1_exon4_exon3_exon2_9dom  WYSXFIFSCRLRCIQTVASVEDFTVENEKNYLYEKGSKEQKELEAALKKYKNDCTEIPIIIGGEEIKTNNVQYQVMVSMFLSEKVIEKFILNTCKRYCRNYDNYKYLFLKXKNISVYTYIIFFKCLQDVIKKAIEKNLQVRRDWEAVPISEKIKLFLKAGDLVSGKYRMDLNAATMLGQVXYCLLCKNNKYRYISMFNFDMYFINSFVLNITFICRNFIQSFSQGKTVYEAEIDSAAELADFFRFNAFFAKVKIFHKRIFFFFFLCVNNLFCHFRNLQNSLYFKYQYTESLSGXILIXGNVVMWKPSDTAILSNYVVMKVLQEAGFPPGVINFVPAEGKTFGDTITSYPSLAGINFTGSVAXVTIILFINCCCFNIIYFHIKCFSQLXYLNIIFQGFIASISPFNFTAIGGNLASAPTLMVXLALFSLYFKYQYTESLSGXILIXGNVVMWKPSDTAILSNYVVMKVLQEAGFPPGVINFVPAEGKTFGDTITSYPSLAGINFTGSVAXVTIILFINCCCFNIIYFHIKCFSQLILKIKNFCILYFKNTVFNSYCYRKFMEEATEQLKMAAGNFYINDKSTGSVVGQQPFGGGRLSGKFXNCVCLNVVFIFXLEIKLEFFFXEQMTKQVDLIFLIRHTRLFNLKIDIYIIFSTFRHLWKQISQNLELYKNFPRMVGGNLIXIXNILXNLKTKCIVIVLLILKXFFLNHLECGGKNFHFVHSSADVESVVGATIRASFGYSGQKCSACSRMYVPDNLWPKVCEYKEIYIMIICYFDNQSIVLDDXIFILKKHFQIKEGLLEEQKKVKLGSPLEYDTFLSAVIDGNSFKRIKSYLDHAKSSSQLEILAGGKCDNRLXLFXILXNSKTYNKFIFFSKGYFIEPSIVQSKDPTDKIMKDELFGPVVSVYVYPEKNVTEALNLVKETTPYALTGSLFTKDEXVRFFSYISYELMSYVMLC |
| *Notodromas monacha* | ASS | No |  |
|  | OAT | No |  |
| *Darwinula stevensoni* | AMD | No |  |
| *Eurytemora affinis* | ASS | No |  |
|  | ASL | No |  |
|  | NOS | No |  |
|  | ODC | No |  |
|  | SRM | No |  |
| *Tigriopus californicus* | ASS | No |  |
|  | ASL | No |  |
|  | ARG | Yes | XP_059096458.1 |
|  | OAT | Yes | XP_059090521.1 |
|  | SRM | No |  |
| *Lepeophtheirus salmonis* | ASS | No |  |
|  | ASL | No |  |
|  | NOS | No |  |
|  | ODC | No |  |
|  | SRM | No |  |
| *Procambarus clarkii* | SRM | Yes | >NC_059631.1_exon5_exon1_exon4_exon2_exon7_exon3_exon6_5dom  XNSIFIEVKVVLLTGKMDENLKGWYTERSPMWPGQALSLQIKQVLHEKQSQFQKIQVLERSDKXLIVVPSLSSVWFGQFSYILLMLYSSXYSPFINKLSVGLVYINDLLPGQGLHYPIHFPYFPIHSDFPANEXNNSFVCGVDSTHYGKVLVLDGAIQFTERDEASYQEMITFLPINSHPNPKKVNHIPVPRRXITLIQEGKSPLSRKVNHPIQYTVVVERARILDEQVILNVWTHSGPIXSILLCCIECTVALLYHLKCIMSSVSDDDFVVVLSNXGVNPQVLIVGGGDGGVARELAKHPLVQEVIQCEIDADVSYFGLTDEVLIRIFLSRKDKIDYLYRYFIVNFGNYNNAKCVLLYCKSEVIQIWVFGTLTRIGIKIKMLYESFWSNQLNGFFPQFSCGNMNCLSWYKILQFFYKKTYKIHDVMHIIRRSIWILXQVIDACKTFVPSMGCGFSNPKLTLHTGDGAEFLEKTDAKFDVIITDASDPVVGIDDGETKDGKMXYHLYNIGLINIVLGPAVSLFNEDYYSRMKDKLAPGGILCCQVGSFYCNGLLLXCLNYNFXHNCTISXGESMWLHAELISRLVTSCRKIFPVVEYAHTCTPTYPSGQIGFILCSKNSVSRICIIIYLLKMXNISVIGSYIMYSGISIQRTSFEMIFVWVFWICLEIARDIIIISFAKSFKGLHLKIYHLSCATIXNKHSIXLDLHMPAHLVWEIVTNFTSVQYLVTLQATNFREPVTVWDDEKANSLSLEYYNAEIHRASFVMPNFMKNALREVKXNEPLLSQ |
| *Homarus americanus* | SMS | Yes | >NW_024711254.1_exon1  XYLLDNINFFLQIIVGDAISHMEEMIKGERRFDYVFADLTDVPISPSPRGELWDFMRKVMNLGTKIMKSDTGKYLTHVS |
| *Folsomia candida* | ASS | No |  |
|  | ASL | No |  |
| *Catajapyx aquilonaris* | ASS | No |  |
|  | ASL | No |  |
|  | SMS | Yes | >scaffold124865_cov117_exon2_exon3_exon1_4dom  DCIVGPGCYFLXYFAVERKTMQQDVEATLKDHLPPETVRRHQDIELADGFLTVMLGPRNSFMTLRVFSHGLVTLNIEYYKNEGDEPLLTLEVFALLASWKRHGQDHTFTNAMLCHKRERRDQYAVLLQQIRSLEIAVKNLFLGRRSKFFPPIRRGADIDVYLTTSSKVGSTPTLFLSVAVNCFELSDHFHPVDLAESDLIYTETLMQRGKENFEGKEILILGGGDGALLWELLKEKPKFVTMIEVWLFEKDLQPQFNIPNFPYRLTRWXWKHAGNIYAAVVATALTTTRGRTMRXCAGSSSSXXWKNFHIVLQSQIIVDDCVKHMKKYQDEGKSFDYIFADLTDIPLSSTPQGEMWDFIRLILQLSFAILKPTGKYMTHVSXFSVRLRHXVQKTQTECRQDMKLVSLQGNGVSSVQSLAMFEDVITSLGAEFTKCDAFVPSFMELWVFYQVTKKMAEGHXKSLSR |
| *Ischnura elegans* | ASS | No |  |
|  | ASL | No |  |
| *Blattella germanica* | SMS | Yes | >PYGN01001050.1_exon6_exon5_exon7_exon4_exon1_exon2_exon3_4dom  FLFCDSEESRGVQDDMESVLANFVPGLKKKSVYDFGGSFMALLTADRGTVVTLRGFPQGLVTCNIEYYKDDEEKPLLLFEVCETWAPLQQIRALESRLKAKLSCCRSKIFPPIKRGASLDVYLTTSGKEVRLILLQSIRKLERELAQKLCSKRSHSIPALKRGYRVDRYFPTAGKRICNINEYNLNQXRLKRHNSVGLMESLSAHSWGLISDERILEYDIDKVLFEEKSPYQKILIVHSRSLGNLLVLDDLQSKFDFFHLLSCRPPGRLKAEPRYXFYLICNXHASTNPDLNQYIFPKILLNARXQSXIYIEETKFFLYLYLIYSVMXLRDICAAVVDMSESDLIYTETLMQRGKENYEGKEIVILGGGDGALLWELLKEKPKFVYMLEVSTIXHNHFRRTCPMRSCITRAPIVIILLLLMGSNFVXSFFXIVVDDCVKVLSKYIEEGRTFDYVFGDLTDIPLSSTPQGEVWDFIRLILNMSMSVLKPDGRYMTHVSYNYSGLKPPWSLAPWMRAVVCHXSVFLKXFQGNGASCPDSLKMYEEQLKKLKVPVEFSRAHAFVPSFMEDWVFYQVWLKQNXQKRRQWLQKA |
|  | AMD | No |  |
| *Thrips palmi* | ASS | No |  |
|  | ASL | No |  |
| *Frankliniella occidentalis* | ASS | No |  |
|  | ASL | No |  |
|  | OAT | Yes | XP_052121103.1 |
| *Nilaparvata lugens* | ARG | No |  |
| *Laodelphax striatellus* | ASS | No |  |
|  | ASL | No |  |
|  | ARG | No |  |
| *Halyomorpha halys* | ASS | No |  |
|  | ASL | No |  |
|  | ARG | No |  |
| *Rhodnius prolixus* | ASL | No |  |
|  | ARG | No |  |
|  | P5CDH | Yes | XJQ61297.1 |
|  | ODC | Yes | >KQ034107_exon4_exon5_exon3_exon1_exon2_4dom  ISIKFHVSLQRYIFCFPCFXILFXSQEEAFFVMDVGEIIKQYETWTKMLPRVKPFYGMLFXKNNRFLAGKGSAVKCNDSQVVLQIMAALGANFDCASKVGTYEIFFLKIIFVYPMIVPPXFXRKXICVLFKIIVIIIVXGEINKVLDLGVTADRIIFANPAKIRSHISYAAKAGVDMVTFDSISELNKIKEFHPNAKYVIIIHLLYLTMRLKYXYNKTNCYSQISAGTKKEFLNIIILVPFNLNIGSLQQCSXNIIYSLLNTFNYNLCSSNIIKGXFFSRLVLRIAADSYDSQCPAMSLKFGVDPEKEAYPLLKEAVAMGLNVVGISFHVGSGCREVEAFGRALAFAAGLFIRAQDGLGLRMSILDIGGGFQMNNFEKVKYKYXLKLIIKIXFIHRXNDEXSVYLHVXXELIVLLKYFSTKFIHKSFSSSSYIFLRLLCFYLKKYXFVXSVHIYXTCATINRALGEYFEDDLELEIIAEPGRFMVASAYTLATQVCYVSKVAKIIICIYIFXSIXHIINILLFPAQIHSMRVNSNQTMLYINDGVYGSFNCLLFDHASVTPIPLKVIHEVAQNVYRTNFIPSKMNLLIALLKRXKCFGDIYRIGYYXIPSLRRQEYIXGLRGLILLIQSGSTFLVNLYVILAIKDFSIFLFLFQENFFGNSLITCSIWGPTCDSLDLVSEACLLPSCLNIGDWILFESMGAYTIPTSTNFNGFAPPKIHHICDFNNWXVIIFXFXLENEVVEXGTLSPQN |
| *Cimex lectularius* | ASS | No |  |
|  | ASL | No |  |
| *Apolygus lucorum* | ASS | No |  |
|  | ASL | No |  |
| *Bemisia tabaci* | ASS | No |  |
|  | ASL | No |  |
|  | ARG | No |  |
| *Daktulosphaira vitifoliae* | ASS | No |  |
|  | ASL | No |  |
|  | ARG | No |  |
|  | SMS | No |  |
| *Eriosoma lanigerum* | ASS | No |  |
|  | ASL | No |  |
|  | ARG | No |  |
|  | SMS | No |  |
| *Sipha flava* | ASS | No |  |
|  | ASL | No |  |
|  | ARG | No |  |
|  | SMS | No |  |
| *Aphis craccivora* | ASS | No |  |
|  | ARG | No |  |
|  | SMS | No |  |
|  | AMD | No |  |
| *Rhopalosiphum maidis* | ASS | No |  |
|  | ASL | No |  |
|  | ARG | No |  |
|  | SMS | No |  |
| *Melanaphis sacchari* | ASS | No |  |
|  | ASL | No |  |
|  | ARG | No |  |
|  | SMS | No |  |
| *Diuraphis noxia* | ASS | No |  |
|  | ASL | No |  |
|  | ARG | No |  |
|  | SMS | No |  |
| *Myzus persicae* | ASS | No |  |
|  | ASL | No |  |
|  | ARG | No |  |
|  | SMS | No |  |
| *Acyrthosiphon pisum* | ASS | No |  |
|  | ARG | No |  |
|  | SMS | No |  |
| *Pediculus humanus corporis* | ASS | No |  |
|  | ASL | No |  |
| *Athalia rosae* | ASS | No |  |
|  | ASL | No |  |
| *Neodiprion lecontei* | ASS | No |  |
|  | ASL | No |  |
| *Diprion similis* | ASS | No |  |
|  | ASL | No |  |
|  | AMD | No |  |
| *Cephus cinctus* | ASS | No |  |
|  | ASL | No |  |
| *Leptopilina heterotoma* | ARG | Yes | XP_043476531.1 |
| *Belonocnema treatae* | ASS | Yes | >NC_046665.1_exon1  NIKKKYKIVNQNIKLKILQLLCIIKRKCEISLYTGTLAXMDVKXDFRLEDSXGFIRIQAVRLKEYQRFKMQXNLNKI |
|  | ASL | No |  |
| *Trichogramma pretiosum* | ASS | No |  |
|  | ASL | No |  |
| *Copidosoma floridanum* | ASS | No |  |
|  | ASL | No |  |
| *Ceratosolen solmsi marchali* | ASS | No |  |
| *Diachasma alloeum* | ASS | No |  |
|  | ASL | No |  |
| *Fopius arisanus* | ASS | No |  |
|  | ASL | No |  |
| *Aphidius gifuensis* | ASS | No |  |
|  | ASL | No |  |
| *Chelonus insularis* | ASS | No |  |
|  | ASL | No |  |
| *Microplitis demolitor* | ASS | No |  |
|  | ASL | No |  |
| *Cotesia glomerata* | ASS | No |  |
|  | ASL | No |  |
| *Ampulex compressa* | ARG | Yes | >JAHFVI010000073.1_exon2_exon1_2dom  SKAKKDVAVIWVDAHADLNTNKTSGSGNVHGMPVALLTSELSDYWPHLPGMDWQMPMXICNNNLLICSYTAMSNFCXYWKIKQXIKXCITIXISFRLSIRNVAYIGLRSVDSYERLVIEKFGITAFGMEDVERYGKTLWKSHLLLFSYIYSLSIQSIGIHDVICMALNKIDPDNSKSLHISFDIDSLDPLEAPSTGTP |
| *Colletes gigas* | ASS | No |  |
|  | ASL | No |  |
| *Dufourea novaeangliae* | ASS | Yes | >NW_015374058.1_exon1  LSSGFWFSPECDFVRNSILYSQKYVNGVVRLQLYKGNGGCKXKKINLIXNGDCTATRKNLILQFLY |
|  | ASL | No |  |
| *Osmia lignaria* | ASS | No |  |
|  | ASL | No |  |
|  | NOS | No |  |
| *Megachile rotundata* | ASS | No |  |
|  | ASL | No |  |
| *Ceratina calcarata* | ASS | No |  |
|  | ASL | No |  |
| *Bombus terrestris* | ASL | No |  |
| *Melipona quadrifasciata* | P5CS | Yes | >KQ435744.1_exon5_exon4_exon1_exon2_exon3_3dom  HRELQTTEGPRKATMFNERSQLSYTRRLVVKLGSAVITREDEHGLALGRLASIVEQVAECQNGGRECIMVTSGAVAFGKQKLAQELLMSLSMRETLSPGGHMRKRSGIILHPYVDTRSIKRTSNLMVQWYRMIRXFRCNIYTGTPLEPRAAAAVGQSGLMSLYDAMFAQYGVKLAQVLVTKPDFYNEETRKNLFSTLTELLSLNIVPIINTNDAVSPPLSVDEEITGRGIPIKDNDSLAAMLAAEIQADLLILMSDVDGIYNLPPWQDGAKMLHTFSMDLRDTIKFGQKSKVGTGGMDAKVNAALWALDRGVSVVICNGTQEKAIKNILSGRKIGTFFTQTTETSTPVEVVAEDGKSFFSFXQTFICFTFECFMEKXKEYFTMPKFYPXIRLSDSRKTQFLSKHXIYDINAKXKNSKMIIYTARIGSRTLQALQPEERASCINTLADLLESRQREILSANMKDLEAAEKSGLAKALLSRLSLTPAKLKSLSSGLRQIANDSLTNVGRVLRRTKLAEGLELKQITVPIGVLLVIFESRPDSLPQVAALAMSSANGLLLKGGKEAANSNRYLMELVKEALNTVGAANAISLISTREDVGDLLSMGKHIDLVIPRGSSDLVRNIQEQSKHIPVLGHAEGICHVYVDKDADLIKALKIVRDSKCDYPAACNAMETLLIHESHMKDSFFTDVCNMLQKEGVRGLTREEWLLLYVTKRLTFXVKVYSGPNLRKQLTFGPPAAKSMKTEYGALECAIEVVSDVDDAINHIHKYGSGHTDTIVTENNHSAAHFQREVDSACVFHNASTRFSDGYRFGLGAEVSHXNEKFKNXKIXTSERLGNSXNLNIKFFYXYXKIQNFKNSTTXSLKISTKYEFFFNFFYYFKVGISTARIHARGPVGVDGLLTTKWVLQGDGHAAADFAEGGNQVWLHQSLPIYESAXIKSEQSENIGIFASETRI |
|  | PRODH |  | >KQ435868.1_exon7_exon3_exon5_exon4_exon6_exon2_exon1_4dom  KFNDPVAAFKSKTTKELLRAYIVYQLCSIEYIVENNMKVSRYFSLLKLFIACYCLRLLHVCLRIVKYFTMYRRXRRKIFLPKKFCRDPPVNNVENFHIIPRDESLIRVYLIVNRIVKFLFLLMKTILGEKLFTVLMKSTFYGHFVAGEDEVQITPVLDRLRQFGVKPILDYSVEEDISQEEAERREIQXVSTVLFPMISCFFFKNGSRMVLICRASVSEAGDEKREGPLKKYHVAKPFADRRYKVSSARTYFYLNEASCERNMDIFIRCLESVAGESIIQVNILKQLQLSEVIMRARQYMSDVVGGEGAVLTHHAKPDDFMKKFEEAQIKDEAPVKKFLQKLQSDKEGXRFIKXKIVHXFATVFEAIKDGEIVTRLLHKSFTFXFHVSLKKKFITQCYKLIRQGTLXFVTLAKTELXILLQVADKLDVRIMIDAEQTYFQPAISRLTLEMMRKYNTRKVNALLLXMRYYHLVNFQWSKRKWSTSXAVVFNTYQTYLQEAFNEVKTDLEQAERQNFYFGAKLVRGAYIEQVIPHIIHRLAFYNFLLLDSFTPDHCVGXHTDQIVSHVGNFCFSDCCIDARVRIKFLKLLMXERARAAAMGYPDPTNPTYEATTDSYHRTLMECLRRMKQYKDKGEDPKKIGIMVASHNEDTVRFAIEKYVSQNILYTYVRFKYVHAELFPFEYTIFENFNNNCTKYEXNKFEXXLHRLNKLNIPFTRVKVIIQTITIYXDILNMLXNRMKEIGISPEDKVICFGQLLGMCDYITFPLGTCLYSVLSNCVGNTTGVNXRIDCLTKLKILGQSGYSAYKYIPYGPVKEVLPYLSRRAQENRGILTKIKKEKRLLLAEILRRFASGQIFYKPKGNYTPV |
|  | AMD | No |  |
| *Frieseomelitta varia* | AMD | No |  |
| *Ooceraea biroi* | ASS | No |  |
|  | ASL | No |  |
| *Eciton burchellii* | ASL | No |  |
| *Pseudomyrmex gracilis* | ASL | No |  |
| *Nylanderia fulva* | ODC | No |  |
| *Formica exsecta* | ASL | Yes | >NW_021850453.1_exon5_exon7_exon6_exon3_exon2_exon1_exon4_5dom  LILFYVHEIKTCQKLWGGRFGEDIDPDFHNFNASIDIDKRLYAEDIQVNIXGSIAYARSLYKAKLISQEEMQTINTALKQNRIFNXXVQDEWENNKFTINHEDEDIHSANERRLINSITNVLNIQELIGDIAKKLHIGRSRNDQTVTDTKLWLRKSIDKLLFRLKNFIEVNKIISFIXVGVLTKQTNTITNXYXSHMLNVNSLLILKILDSHSSSILNVYSVXKTCNLXNFFVYYFSINNEQWLKFFSGYSGWXPXQHICQGQDMIRFGSYSKYLPLILSLIFQISYSYAWYAKQDLERLLEIRKHVNILPLGSGAIAGNPFAIDRQFLAMELGFDEITENSMHTVGDRDFVGMFYKYISVTCVHIYAYIHRASRLTIETINISDSIEXKNVXLYDSQMQIRHKNINKYYSLRIIPKLSIYLPIFSLSLLINIKNLLFSYIIKFSYERRSTFIVQFFDFRYYLSLFVIQFXIKVLAKXKICLKLTKKRNVNXKKNRFEVTKNANFEYKINKILPLYCTYSVXTFFNLIFXAEFLFWSSLSSMHLSRFCEDLIIYSTQEFNFIQFSDKYSTGSSLMPQKRNPDCMELIRGKTGTIFGKVCLNRFKTIGSIYGSYVCIFYLSLSQCLVIXNLRKIVNFSLSYKITAXIKFRVIMLQFYKHIFSSFSLVKLSXFXCIGFMVTLKGIPSTYNKDLQEDKESLFYTYDTLYQMFHIAEKALATLKVNSKNCKKALTSDMLATDMAYYLVKKGVSILFFFLSLKNLILLDNFXGNCFTNLISLNFNXMLIEISLNICVNTQISFRESHHLAGKAVALAESKGIPLLELSVQQLKTIRYIIKIFNRDEVVAFEENSKRKINYFSEAFEENISCIWDFNSSVDQYKIAGGTSFEAVQQQINSLRCWLRNYSFK |
| *Pogonomyrmex barbatus* | ASL | No |  |
| *Monomorium pharaonis* | ASL | No |  |
| *Temnothorax longispinosus* | ASS | Yes | >QBLH01003561.1_exon2_exon1_exon3_3dom  XKTELXKMSESKGKVILAYSGGLDTSCILLWLKEKGYQVIAYVVRNFVITMXILSAASRHKNLXFVXANIGQKEDFDAIRDKALKVGAAKVKIIFLPNILXYKIIXRYXKYPYAINFIFITKQTNSGLSLKNLXDGVSNRIFLICSYESGILENPAVPAPKGLYKMTVDPTDSALEAEEIEITFKTGHPINVKSLKDGKMFNVPLAIIEYLNEVGGAHGIGRIDIVENRYIGVKVYFLXNELYERIKLIVTLFVQTFQSRGIYESPGVNILKIAHRDLEVFILDREVLRVKSYLTDKMSDYVYNGSQIRESAIYIXLXDKIXKLXKYLXTKWIKLMKXMLCRFLVFAGMRLRTRMHPAFAKICKRHSETAXLFFMSMVIKGISFPLIAMAIARSSEVSLYNQTLVSMDVQGDFEPTDATGFIRTQALRLKEFHRFNKQYNVXIKIL |
|  | ASL | No |  |
|  | ARG | Yes | >QBLH01000142.1_exon4_exon5_exon3_exon2_exon1_2dom  DTXFKMNVLTKARTISSKLGIRYYSGKVGIIGVPFDKGQVNVKYAKQKKEGVAHGPEAIRAARLVDELKILGKSATMCDVYLFYKYIYIYFIKKELDVRDYGNVFYSANNIDDVKNMSHLGHVASCMSSLSEQIRQVLRDGRQALTIGGDHSLSIGTIDGHVKVCKMEIQDVAVVWVDAHADLNTNKTSESGNVHGMPVALLTNCALIVAXNRSMHLTDILLGIHDVIHMALNKIDPHNSRSLHVSFDIDVLDPLEAPSTGTPGLQYIILMYMHTYMHYSYYKSLYLHICTVRGGLSLREAIHLMEEIHRTHRLSAIDLVEVNPQIGSERDVEMTVQAAIHIIQAALGYTRRGLRVPKGITDMPLQTFH |
|  | SMS | Yes | >QBLH01001859.1_exon4_exon6_exon5_exon1_exon2_exon3_6dom  IAVSPSGLTSHILYLCSYIAEAMVAHTVLLDFTVSSSVIADMEKRSGLKSAIANVLAEHFTGLKPLTESNIDGSLLVLYTGPRGSLITVRGYTEGLITLNIEYYKQDDQEALLTFEVCMHELDKQFAVNVRKISVEERTLVLLRNTTYLLRTLXQWRYLEADVAMALNSQRSKRLPPVRRGTIYDLYLTLSGRKXLAKELETALQTAATATRSHTLTPIKRGGPFERYYPTAGEFLFRRTPKXVVLCYNFVYYLQLTRNSSADMSEADLIYTETLMQRGKENYAGKEIVILGGGDGGLLWELLKEKPKFITMLEVCESNFTRELYERIICTICFYIFFLQIDDIVMKACSQHMRSICGDCLDKRKGDNYEVSFVLXIFKRLNSKNRAFFNLNRNNNNLSLXIIVGDCVKALAHMIEEGRQFDYVFGDLTDIPISTTPHGDAWDFIRLILNSSMKVLKSTGKYMTHVSIFIHFQSTFNFIYYAIKWXYCFFFCMXGNGASCPESLKMYEEVLSQLCVPVTFTKDRAFVPSFFEDWIFYQVSLKXWSKEYS |
| *Cyphomyrmex costatus* | ASS | No |  |
|  | ASL | No |  |
| *Trachymyrmex septentrionalis* | ASL | No |  |
| *Atta colombica* | ASL | No |  |
| *Pseudoatta argentina* | ASS | Yes | >JAANIA010001464.1_exon1_exon2_exon3_3dom  YDIREVFITSYVXPTVACSXLLYKGRYLLGTSTTMYQPKFNKSHKGXRFIAHGATGKWNDQIRFELNCYNLYLKITVLXTLXIDFIKDFTSDKKQMKNIXGLKNKSNRSFIYKFFYSCRNVPYSCFTSIKIQCXNIIVSNFKLIIPKKLSRFXLHGEKSNFTNFKEVPRKIGLAGVRTTNGISIFATAKELWNTDAKLVHIYNFNLYSYESRILEDPAVPAPKGHYKMTIDPIYSSFFFLTAMAIARSSEVSLYNXILVSMNATEFIRTQALKLKEFYRFNKQHNV |
|  | ASL | No |  |
| *Acromyrmex echinatior* | ASL | No |  |
| *Agrilus planipennis* | ASS | No |  |
|  | ASL | No |  |
| *Coccinella septempunctata* | ASS | No |  |
|  | ASL | No |  |
|  | ARG | No |  |
| *Brassicogethes aeneus* | ASS | No |  |
|  | ASL | No |  |
| *Rhynchophorus ferrugineus* | AMD | No |  |
| *Diabrotica virgifera virgifera* | ASS | No |  |
|  | ASL | No |  |
|  | SMS | No |  |
| *Gonioctena quinquepunctata* | SMS | No |  |
| *Aedes aegypti* | AMD | No |  |
| *Contarinia nasturtii* | ASS | No |  |
|  | ASL | No |  |
| *Bradysia coprophila* | ASS | No |  |
| *Zeugodacus cucurbitae* | ARG | Yes | XP_011177103.1 |
| *Glossina fuscipes* | ASS | No |  |
| *Zerene cesonia* | ODC | No |  |
| *Hyposmocoma kahamanoa* | AMD | No |  |
| *Chilo suppressalis* | P5CDH | Yes | >RSAL01000083.1_exon1  SIVELPKLQNFDVQNEPILGYREGSQERNMLKEELARAAAVTEDVPIVIGDEEVRDGEPRFQVMPHNHSKKIAKFYYASEKTIQKAIKVSVAAQERWDRTSLEERVRIWQCAAELMAGRYRQSLNAATMLGQSKTAIQAEIDSAAELIDFFRFNVFFLKENAKYQPISENAKVTRNSLRFRGIDGFIAAISPFNFTAIGGNLAYTPALMGNGVVWKPSDTALLSNWRIFNIMREAGLPPGVVNFVPADGPTFGKTITSSPDLAGINFTGSVPTFNWLWNAVGQNLSKYRNYPRLIGECGGKNYHFVHPSADVQSVVTGTIRSAFEYCGQKCSACSRMYVPKSLFEPIKNGLVTERNKLKIGDPSDFRVFTGAVIDDKAFARISGYIKGAKSNSKNKILAGGKCDDSKGYFVEPTIIQTEDPLDKLMTEEIFGPVLTVYVYEDADVHKTLSMVGESTKFALTGAVFSQDKDFSKVALDVLKTTAGNFYINDKSTGSVVGQQPFGGGRMSGTNDKAGGPNYVMRWTSPQSIKETFVPLRDIDYPYMRDXFMLLQIYRXLKHHNQYISTIINVSY |

**Supplementary Material 3** - Sequence alignment among all insect amino acid sequences classified as OTC in the KEGG Orthologous database (KO ID: K00611) and reference sequences from *Bos taurus* (Uniprot ID: Q9N1U7), *Homo sapiens* (Uniprot ID: P00480), *Saccharomyces cerevisiae* (Uniprot ID: P05150), *Escherichia coli* (Uniprot ID: P06960), *Pseudomonas aeruginosa* (Uniprot ID: P08308), *Gallus gallus* (Uniprot ID: Q9YHY9), and *Arabidopsis thaliana* (Uniprot ID: O50039). Amino acids composing the catalytic site in *H. sapiens* are highlighted in blue, in *E. coli* in red, and residues common to both are highlighted in purple. Stars indicate the catalytic residues used as filters for our sequence selection

**
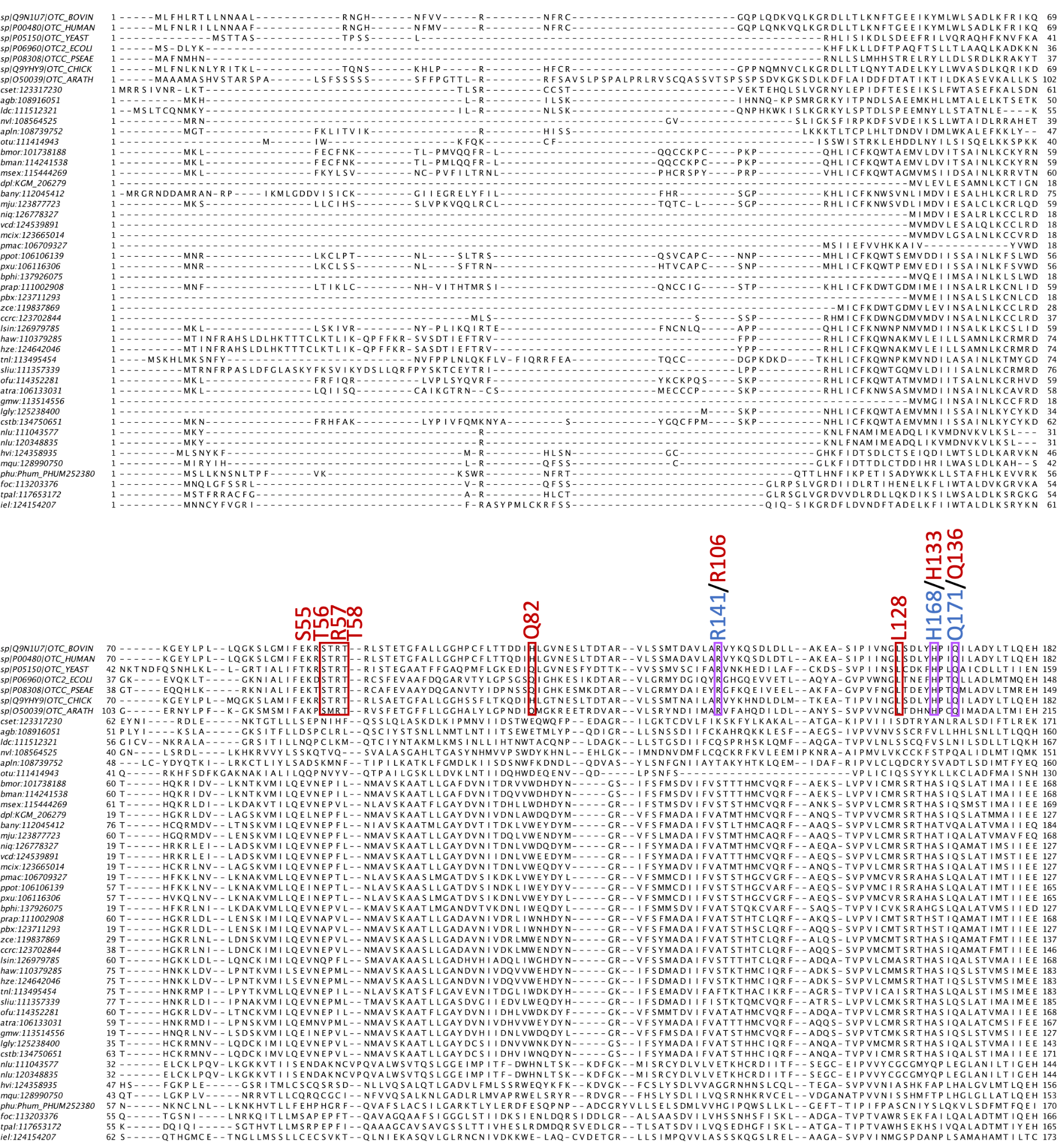
**

(Continue)

(Continuation)

**
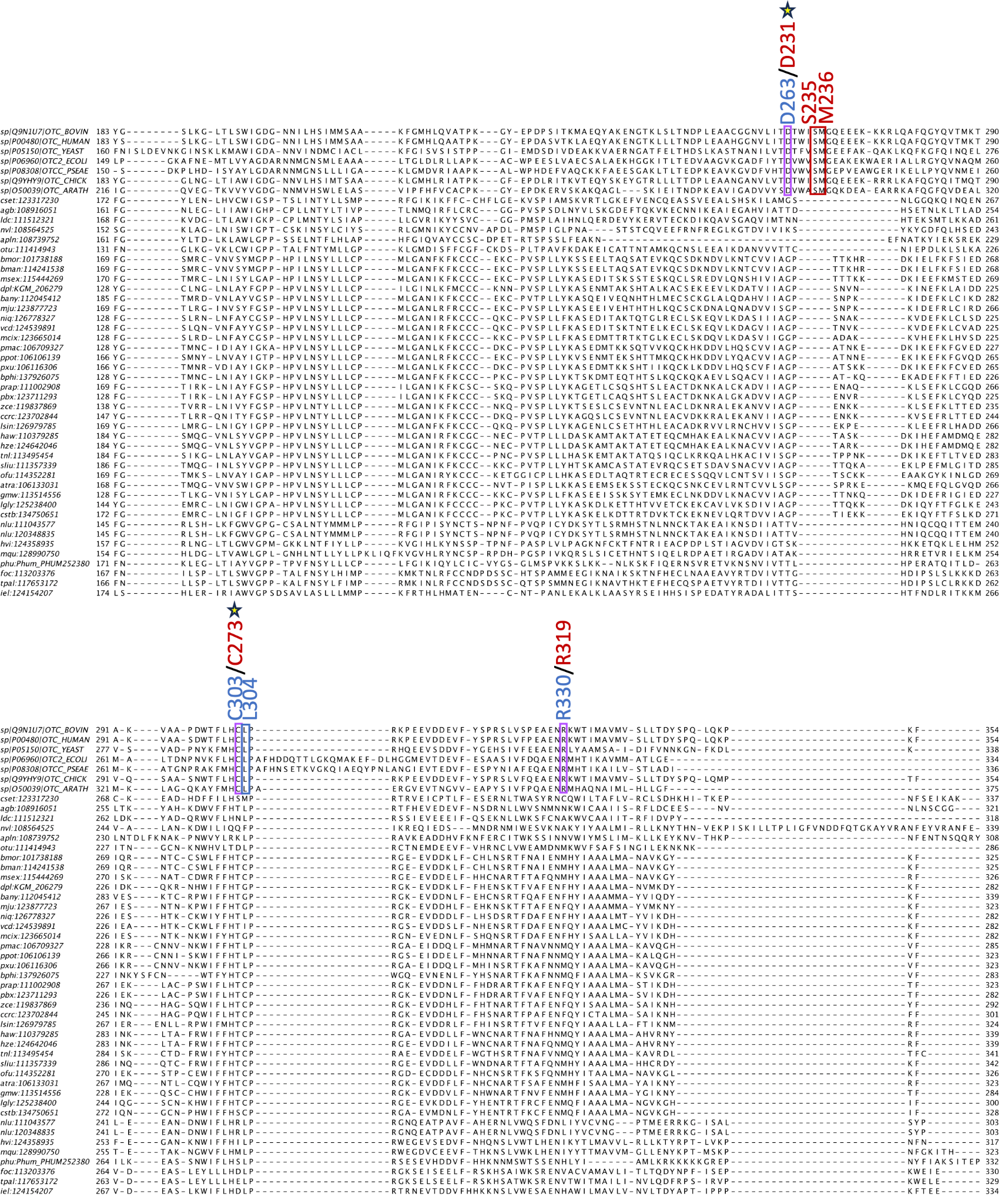
**

**Supplementary Material 4 -** Tridimensional structure alignment between the *H. sapiens* OTC structure (PDB ID: 1C9Y) (blue) and the predicted structure of *Helicoverpa armigera* putative OTC (yellow) generated using AlphaFold2. The catalytic sites predicted for the human OTC are highlighted in red.

**
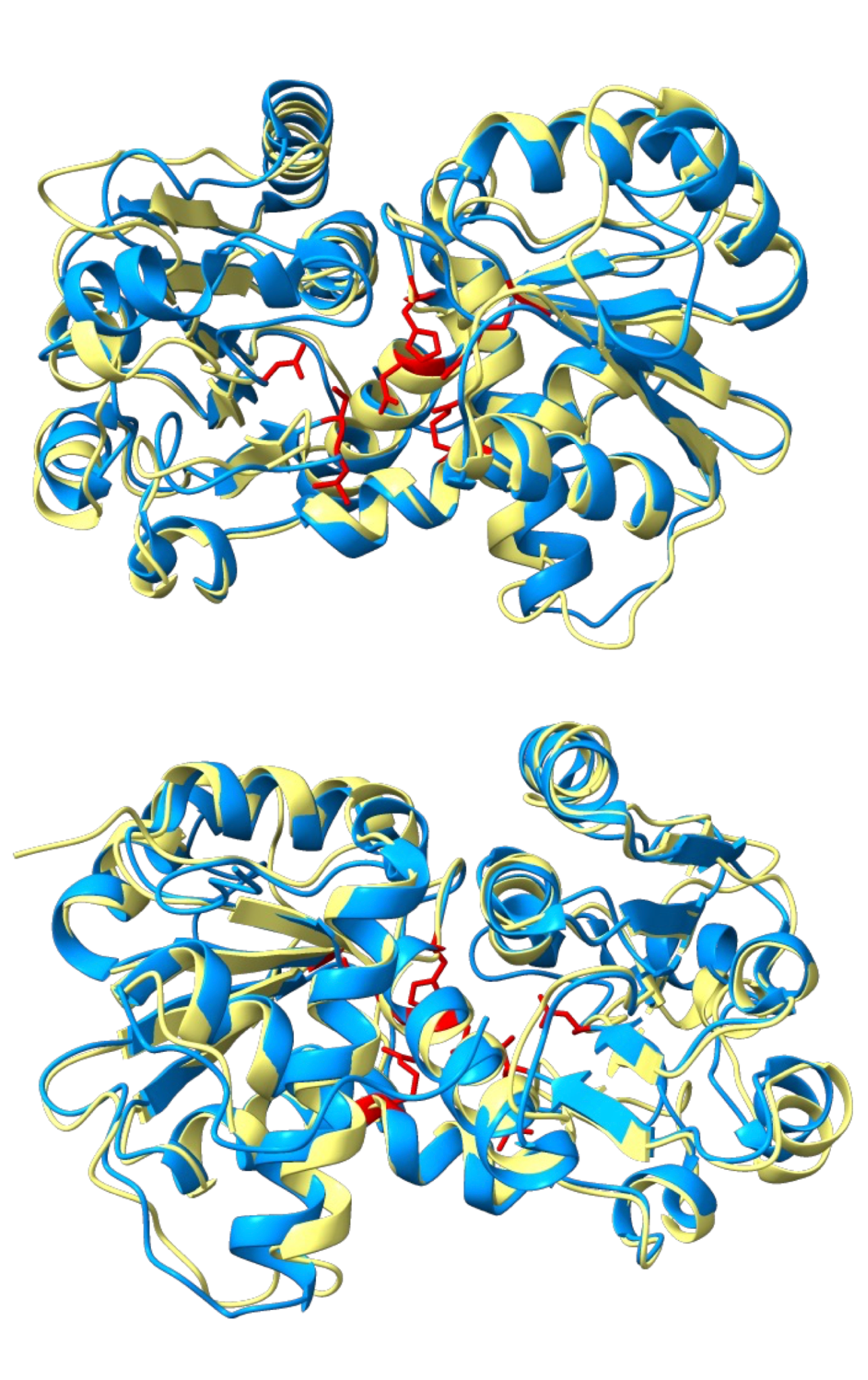
**

**Supplementary Material 5 -** Best hits results for BLAST search of proteins found in *R. prolixus* scaffold KQ036543 and TPM values expressed per tissue.

|  | **Sequence ID** | **RPRC003927** | **RPRC006164** | **RPRC012901** |
| --- | --- | --- | --- | --- |
| **BLAST results** | **Description** | Argininosuccinate synthase | dTDP-glucose 4,6-dehydratase | thiol:disulfide interchange protein DsbA |
|  | **Max Score** | 557 | 451 | 410 |
|  | **Total Score** | 557 | 451 | 410 |
|  | **Query Cover** | 100 | 87% | 100% |
|  | **E-Value** | 0.0 | 5^-138 | 6^-144 |
|  | **Similarity (%)** | 97.16 | 97.92% | 97.10% |
|  | **Scientific Name** | *Kluyvera cryocrescens* | *Symbiopectobacterium purcellii* | *Candidatus Symbiopectobacterium* sp. NZEC135 |
|  | **Order** | Enterobacterales | Enterobacterales | Enterobacterales |
| **Average TPM values per tissue** | **IA** | 0 | 0 | 0 |
|  | **IM** | 0.0433 | 0 | 0 |
|  | **IP** | 0 | 0 | 0 |
|  | **Car** | 0 | 0 | 0 |

**Supplementary Material 6 -** Best hits results for BLAST search of proteins found in *Aphis craccivora* scaffold VUJU01007234.1 and TPM values expressed per tissue.

| **Sequence ID** | **KAF0746381.1** | **KAF0746382.1** | **KAF0746383.1** | **KAF0746384.1** | **KAF0746380.1** |
| --- | --- | --- | --- | --- | --- |
| **Description** | Acetylornithine deacetylase | N-acetyl-gamma-glutamyl-phosphate reductase | Argininosuccinate lyase | Serine acetyltransferase | methylenetetrahydrofolate reductase |
| **Max Score** | 565 | 1091 | 932 | 555 | 434 |
| **Total Score** | 565 | 1091 | 932 | 555 | 434 |
| **Query Cover** | 100% | 100% | 100% | 76% | 100% |
| **E-Value** | 0.0 | 0.0 | 0.0 | 0.0 | 4^-152 |
| **Similarity (%)** | 88.71% | 52.77% | 100% | 100% | 100% |
| **Scientific Name** | *Buchnera aphidicola* | *Klebsiella pneumo* | *Buchnera aphidicola* | *Buchnera aphidicola* | *Buchnera aphidicola* |
| **Order** | Enterobacterales | Enterobacterales | Enterobacterales | Enterobacterales | Enterobacterales |

**Supplementary Material 7 -** Results from BLASTp searches of the unique ASS or ASL sequences found in non-Hemiptera insects.

| **Species** | **ASS** | **ASL** | **Best hit of the found gene** | **Status as contamination** |
| --- | --- | --- | --- | --- |
| **Hymenoptera** | | | | |
| *Bombus terrestris* | XP_020721345.1 | Absent | *Bombus affinis* (Hymenoptera) | Record removed from NCBI |
| *Ceratosolen solmsi marchali* | Absent | XP_011504123.1 | *Enterobacter cloacae* (Enterobacterales) | Record removed from NCBI |
| *Temnothorax longispinosus* | Absent | TGZ51724.1 | *Vollenhovia emeryi (*Hymenoptera*)* | Not a contamination |
| *Eciton burchellii* | KAH0953883.1 | Absent | *Linepithema humile* (Hymenoptera) | Not a contamination |
| *Pseudomyrmex gracilis* | XP_020283146.1 | Absent | *Formica exsecta* (Hymenoptera) | Not a contamination |
| *Formica exsecta* | XP_029668323.1 | Absent | *Cataglyphis hispanica* (Hymenoptera) | Not a contamination |
| *Pogonomyrmex barbatus* | XP_036138948.1 | Absent | *Wasmannia auropunctata* (Hymenoptera) | Not a contamination |
| *Trachymyrmex septentrionalis* | XP_018351323.1 | Absent | *Wasmannia auropunctata* (Hymenoptera) | Not a contamination |
| *Atta colombica* | XP_018049270.1 | Absent | *Atta cephalotes* (Hymenoptera) | Not a contamination |
| *Pogonomyrmex barbatus* | XP_036138948.1 | Absent | *Wasmannia auropunctata* (Hymenoptera) | Not a contamination |
| *Atta colombica* | XP_018049270.1 | Absent | *Atta cephalotes* (Hymenoptera) | Not a contamination |
| *Acromyrmex echinatior* | XP_011052578.1 | Absent | *Vollenhovia emeryi* (Hymenoptera*)* | Not a contamination |
| **Diptera** | | | | |
| *Glossina fuscipes* | Absent | XP_037895385.1 | *Lucilia cuprina* (Diptera) | Not a contamination |
| *Bradysia coprophila* | Absent | XP_037033424.1 | *Halteromyces radiatus* (Diptera) | Not a contamination |

**Supplementary Material 8 -** Best hits results for BLASTp search of putative arginase proteins from *Cimex lectularius* (XP_014242461.1) and *Apolygus lucorum* (KAF6204566.1).

| **Query ID** | **Accession** | **Query Cover** | **E-**  **Value** | **Similarity** | **Species** | **Lineage** |
| --- | --- | --- | --- | --- | --- | --- |
| KAF6204566.1 | BES93014.1 | 81% | 0.0 | 82.62% | *Nesidiocoris tenuis* | Heteroptera;  Cimicomorpha; Miridae |
|  | XP_014242461.1 | 81% | 0.0 | 70.50% | *Cimex lectularius* | Heteroptera; Cimicomorpha; Cimicidae |
|  | XP_046663880.1 | 77% | 2E-131 | 56.60% | *Homalodisca vitripennis* | Auchenorrhyncha;  Cicadellidae |
|  | XP_054262818.1 | 90% | 2E-128 | 51.89% | *Macrosteles quadrilineatus* | Auchenorrhyncha;  Cicadellidae |
| XP_014242461.1 | KAF6204566.1 | 70.50% | 0.0 | 85% | *Apolygus lucorum* | Heteroptera;  Cimicomorpha; Miridae |
|  | BES93014.1 | 68.22% | 0.0 | 85% | *Nesidiocoris tenuis* | Heteroptera;  Cimicomorpha; Miridae |
|  | XP_046663880.1 | 53.57% | 1E-118 | 83% | *Homalodisca vitripennis* | Auchenorrhyncha;  Cicadellidae |
|  | XP_054262818.1 | 51.18% | 3E-111 | 81% | *Macrosteles quadrilineatus* | Auchenorrhyncha;  Cicadellidae |

**Supplementary Material 9 -** Expression profile of the genes involved in arginine biosynthesis and metabolism in *Spodoptera frugiperda*.

| **Enzyme** | **Protein ID** | **Expression (TPM)** | | | |
| --- | --- | --- | --- | --- | --- |
|  |  | **AM** | **MM** | **PM** | **CAR** |
| **ASS** | XP_035430658.1 | 27.9 | 7.3 | 0.9 | 511.7 |
| **ASL** | XP_035436057.1 | 2.3 | 0.8 | 0.4 | 31.4 |
| **NOS** | XP_035436564.1 | 2.1 | 0.9 | 0.8 | 8.6 |
|  | XP_035436572.1 | 0.9 | 0.5 | 1.1 | 6.0 |
| **ARG** | XP_035429469.1 | 1.6 | 0.2 | 0.1 | 26.7 |
| **OAT** | XP_035444448.1 | 10.10 | 354.60 | 244.10 | 59.20 |
| **P5CS** | XP_035450343.1 | 70.90 | 29.60 | 297.20 | 104.20 |
| **P5CDH** | XP_035429984.1 | 5.9 | 3.4 | 3.3 | 37.5 |
|  | XP_035429985.1 | 6.8 | 2.8 | 4.2 | 36.7 |
| **PRODH** | XP_035447897.1 | 1.6 | 0.9 | 1.9 | 9.1 |
|  | XP_035448331.1 | 1.2 | 0.7 | 1.2 | 6.0 |
| **PYCR** | XP_035440612.1 | 1.10 | 0.40 | 0.70 | 2.50 |
|  | XP_035457014.1 | 75.80 | 386.70 | 302.10 | 264.00 |
| **ODC** | XP_035451660.1 | 51.1 | 89.4 | 90.5 | 56.6 |
| **SRM** | XP_035433962.1 | 7.3 | 5.7 | 5.7 | 13.1 |
| **SMS** | XP_035458503.1 | 5.1 | 7.0 | 6.0 | 12.2 |
| **AMD** | XP_035443840.1 | 17.6 | 14.6 | 11.6 | 110.1 |

Expression data from RNA-sequencing (RNA-seq) [average transcript per million (TPM) values from three biological replicates], where AM, MM, and PM, anterior, middle and posterior midgut, respectively; and CAR, carcass (larvae less midgut).

TPM values of each gene are highlighted with different shades of green in the Tables so that one can, at a glance, know in which midgut section or carcass it is most expressed.

**Supplementary Material 10 -** Expression profile of the genes involved in arginine biosynthesis and metabolism in *Periplaneta americana.*

| **Enzyme** | **Protein ID** | **Expression (TPM)** | | | | | | | |
| --- | --- | --- | --- | --- | --- | --- | --- | --- | --- |
|  |  | **GC** | **SG** | **HG** | **AM** | **PM** | **FG** | **MT** | **CAR** |
| **ASS** | PaOGS27282 | 0.8 | 0.8 | 3.7 | 0.0 | 0.0 | 4.7 | 151.8 | 53.4 |
|  | PaOGS30577 | 0.2 | 0.1 | 0.5 | 0.0 | 0.6 | 4.7 | 120.0 | 46.4 |
| **ASL** | PaOGS03537 | 0.0 | 0.0 | 0.0 | 0.0 | 0.0 | 0.0 | 0.0 | 0.0 |
|  | PaOGS03540 | 0.0 | 0.0 | 0.0 | 0.0 | 0.0 | 0.0 | 0.0 | 0.0 |
|  | PaOGS22235 | 0.0 | 0.0 | 0.0 | 0.0 | 0.0 | 0.0 | 0.0 | 0.0 |
|  | PaOGS26486 | 0.0 | 0.0 | 0.0 | 0.0 | 0.0 | 0.0 | 0.0 | 0.0 |
| **NOS** | PaOGS16265 | 0.0 | 0.0 | 0.0 | 0.0 | 0.0 | 0.0 | 0.0 | 0.0 |
|  | PaOGS33165 | 0.1 | 4.5 | 0.1 | 1.4 | 1.7 | 0.8 | 0.0 | 1.9 |
| **ARG** | PaOGS31682 | 0.2 | 1.4 | 0.2 | 0.3 | 0.8 | 2.3 | 6.7 | 37.4 |
|  | PaOGS31683 | 0.0 | 0.2 | 0.0 | 0.4 | 0.3 | 0.2 | 0.1 | 3.5 |
| **OAT** | PaOGS24101 | 39.3 | 8.2 | 2.4 | 2.2 | 1.8 | 23.1 | 39.7 | 125.6 |
| **P5CS** | PaOGS14419 | 0.1 | 1.2 | 0.4 | 0.0 | 0.0 | 1.7 | 3.8 | 20.3 |
|  | PaOGS36001 | 0.1 | 1.4 | 0.8 | 0.0 | 0.0 | 0.4 | 2.0 | 16.9 |
| **P5CDH** | PaOGS00359 | 42.4 | 46.1 | 44.0 | 63.4 | 82.6 | 133.8 | 210.3 | 55.2 |
| **PRODH** | PaOGS02710 | 1.8 | 31.7 | 23.2 | 3.2 | 7.5 | 62.8 | 17.8 | 26.6 |
| **PYCR** | PaOGS01722 | 11.0 | 14.8 | 20.5 | 14.0 | 25.5 | 33.8 | 15.6 | 14.8 |
|  | PaOGS29221 | 0.4 | 1.6 | 0.6 | 0.0 | 0.2 | 0.6 | 13.4 | 45.5 |
| **ODC** | PaOGS00385 | 0.0 | 0.1 | 0.5 | 0.0 | 0.1 | 0.0 | 0.0 | 0.7 |
|  | PaOGS05915 | 21.7 | 27.8 | 50.3 | 31.2 | 57.0 | 74.2 | 68.0 | 69.1 |
|  | PaOGS28565 | 8.7 | 11.8 | 18.6 | 12.4 | 21.6 | 29.9 | 30.9 | 32.5 |
| **SRM** | PaOGS02053 | 1.3 | 0.6 | 0.9 | 2.1 | 4.4 | 2.3 | 1.4 | 4.2 |
|  | PaOGS28151 | 3.6 | 0.9 | 1.3 | 2.5 | 4.9 | 3.0 | 1.6 | 6.4 |
| **SMS** | PaOGS30743 | 1.4 | 1.2 | 0.0 | 6.2 | 5.3 | 4.3 | 10.3 | 2.3 |
| **AMD** | PaOGS20852 | 8.3 | 10.8 | 8.0 | 13.1 | 21.2 | 20.5 | 35.1 | 9.1 |

Expression data from RNA-sequencing (RNA-seq) [average transcript per million (TPM) values from three biological replicates], where GC, gastric cecum; SG, salivary gland; HG, hindgut; AM and PM, anterior and posterior midgut, respectively; FG, foregut; MT, Malpighi Tubule; and CAR, carcass (adult less the previously mentionated parts, head, legs and wings).

TPM values of each gene are highlighted with different shades of green in the Tables so that one can, at a glance, know in which midgut section or carcass it is most expressed.

**Supplementary Material 11 -** Expression profile of the genes involved in arginine biosynthesis and metabolism in *Rhodnius prolixus.*

| **Enzyme** | **Gene ID** | **Expression (TPM)** | | | |
| --- | --- | --- | --- | --- | --- |
|  |  | **AM** | **MM** | **PM** | **CAR** |
| **ASS** | Not found | | | | |
| **ASL** |  |  |  |  |  |
| **ARG** |  |  |  |  |  |
| **NOS** | RPRC003886 | 0.2 | 0.2 | 0.2 | 5.4 |
|  | RPRC003942 | 0.0 | 0.57 | 0.0 | 2.0 |
| **OAT** | RPRC003932 | 6.06 | 22.26 | 15.29 | 22.77 |
|  | RPRC005617 | 8.61 | 9.82 | 3.77 | 25.65 |
| **P5CS** | RPRC017364 | 11.0 | 1.7 | 0.9 | 92.4 |
| **P5CDH** | RPRC001985* (PQ463010) | 1.6 | 3.8 | 5.5 | 49.5 |
| **PRODH** | RPRC001370 | 0.6 | 0.4 | 0.4 | 1.7 |
|  | RPRC011234 | 1.2 | 1.7 | 1.1 | 7.3 |
|  | RPRC009250 | 0.0 | 0.0 | 0.0 | 10.4 |
| **PYCR** | RPRC005640 | 2.60 | 4.93 | 2.04 | 15.71 |
|  | RPRC015317 | 220.3 | 993.1 | 589.0 | 1028.8 |
|  | RPRC009314 | 1.7 | 0.3 | 0.3 | 100.5 |
| **ODC** | RPRC009690* (PQ463011) | 58.7 | 61.6 | 58.1 | 274.1 |
| **SRM** | RPRC014159 | 11.5 | 2.2 | 3.1 | 45.9 |
| **SMS** | RPRC000830 | 11.8 | 5.6 | 3.6 | 17.9 |
| **AMD** | RPRC001223 | 0.7 | 2.6 | 1.7 | 10.6 |

Expression data from RNA-sequencing (RNA-seq) [average transcript per million (TPM) values from three biological replicates], where AM, MM, and PM, anterior, middle and posterior midgut, respectively; and CAR, carcass (adult less the previously mentioned parts, head, legs and wings).

TPM values of each gene are highlighted with different shades of green in the Tables so that one can, at a glance, known in which midgut section or carcass it is most expressed.

* Genome sequence lacking the active site, with active site residues identified in our transcriptome provided in parentheses.

**Supplementary Material 12 -** Expression profile of the genes involved in arginine biosynthesis and metabolism in *Tenebrio molitor.*

| **Enzyme** | **Protein ID** | **Expression (TPM)** | | | |
| --- | --- | --- | --- | --- | --- |
|  |  | **AM** | **MM** | **PM** | **CAR** |
| **ASS** | CAH1379689.1 | 19.26 | 15.36 | 14.55 | 79.86 |
| **ASL** | CAH1375267.1 | 0.52 | 0.09 | 0.65 | 70.50 |
| **NOS** | CAH1374762.1 | 178.39 | 106.73 | 38.26 | 6.24 |
| **ARG** | CAH1368839.1 | 0.59 | 0.21 | 0.36 | 33.94 |
| **OAT** | CAH1381476.1 | 154.9 | 169.0 | 77.0 | 230.9 |
| **P5CS** | CAH1372972.1 | 152.4 | 119.4 | 90.9 | 157.6 |
| **P5CDH** | CAH1380301.1 | 9.3 | 8.5 | 9.1 | 110.3 |
| **PRODH** | CAH1382571.1 | 0.5 | 0.5 | 1.0 | 52.3 |
| **PYCR** | CAH1373134.1 | 15.7 | 12.8 | 11.5 | 10.1 |
|  | CAH1372158.1 | 40.2 | 25.3 | 23.8 | 118.7 |
| **ODC** | CAH1365079.1 | 92.8 | 79.1 | 74.5 | 207.2 |
| **SRM** | CAH1382431.1 | 7.1 | 6.7 | 6.8 | 53.8 |
| **SMS** | CAH1381688.1 | 33.8 | 28.1 | 29.5 | 98.5 |
| **AMD** | CAH1376975.1 | 15.8 | 12.2 | 12.1 | 20.1 |

Expression data from RNA-sequencing (RNA-seq) [average transcript per million (TPM) values from three biological replicates], where AM, MM, and PM, anterior, middle and posterior midgut, respectively; and CAR, carcass (larvae less midgut).

TPM values of each gene are highlighted with different shades of green in the Tables so that one can, at a glance, known in which midgut section or carcass it is most expressed.

**Supplementary Material 13 -** Expression profile of the genes involved in arginine biosynthesis and metabolism in *Musca domestica*.

| **Enzyme** | **Protein ID** | **Expression (TPM)** | | | | | | | | |
| --- | --- | --- | --- | --- | --- | --- | --- | --- | --- | --- |
|  |  | **AM1** | | **AM2** | **MM1** | **MM2** | **PM1** | **PM2** | **PM3** | **Car** |
| **ASS** | XP_005191602.3 | | 0.2 | 0.1 | 1.9 | 0.0 | 0.0 | 1.0 | 1.8 | 99.3 |
| **ASL** | XP_005181580.1 | | 0.4 | 0.1 | 0.7 | 0.4 | 0.1 | 0.7 | 1.4 | 53.8 |
| **NOS** | XP_005184458.3 | | 0.0 | 0.0 | 0.0 | 0.0 | 0.0 | 0.0 | 0.0 | 0.4 |
|  | XP_019892832.1 | | 0.0 | 0.0 | 0.0 | 0.0 | 0.0 | 0.0 | 0.1 | 0.6 |
| **ARG** | XP_005180026.1 | | 0.2 | 0.1 | 1.2 | 0.7 | 0.4 | 0.3 | 0.4 | 69.3 |
| **OAT** | XP_005180511.1 | | 6.4 | 7.2 | 13.1 | 17.3 | 28.4 | 62.5 | 52.9 | 182.7 |
| **P5CS** | XP_005189354.2 | | 10.2 | 11.4 | 84.9 | 86.9 | 2.4 | 1.3 | 5.5 | 67.8 |
|  | XP_005175053.2 | | 13.2 | 13.2 | 95.8 | 90.8 | 3.6 | 0.8 | 7.3 | 67.6 |
|  | XP_005189354.2 | | 10.2 | 11.4 | 84.9 | 86.9 | 2.4 | 1.3 | 5.5 | 67.8 |
| **P5CDH** | XP_005180747.1 | | 0.0 | 0.0 | 0.0 | 0.0 | 0.0 | 0.0 | 0.0 | 0.0 |
|  | XP_005187618.1 | | 1.8 | 0.5 | 1.2 | 1.7 | 0.8 | 1.2 | 2.4 | 21.3 |
| **PRODH** | XP_019892511.1 | | 0.7 | 0.2 | 0.7 | 1.0 | 0.9 | 0.8 | 2.3 | 9.8 |
| **PYCR** | XP_005175930.1 | | 3.4 | 4.7 | 12.9 | 26.8 | 2.8 | 5.9 | 10.2 | 20.5 |
|  | XP_005190974.1 | | 12.6 | 13.7 | 23.6 | 33.2 | 17.1 | 24.7 | 40.7 | 29.4 |
| **ODC** | NP_001273820.1 | | 0.0 | 0.1 | 0.4 | 0.1 | 0.0 | 0.0 | 2.5 | 14.0 |
|  | XP_011294312.1 | | 0.0 | 0.0 | 0.0 | 0.0 | 0.0 | 0.0 | 0.0 | 0.0 |
|  | XP_005187899.1 | | 0.1 | 0.0 | 0.0 | 0.0 | 0.0 | 0.1 | 54.2 | 26.8 |
| **SRM** | XP_005191411.1 | | 0.1 | 0.0 | 0.3 | 0.3 | 0.2 | 0.3 | 0.5 | 16.3 |
| **SMS** | XP_005188877.1 | | 0.95 | 0.54 | 3.36 | 8.06 | 0.74 | 1.16 | 12.36 | 4.54 |
| **AMD** | XP_005175677.1 | | 1.2 | 1.1 | 2.5 | 3.3 | 1.9 | 2.5 | 3.8 | 10.8 |

Expression data from RNA-sequencing (RNA-seq) [average transcript per million (TPM) values from three biological replicates], where AM2 and AM2, first and second portions of the anterior midgut, respectively; MM1 and MM2, first and second portions of the middle midgut, respectively; PM1, PM2, and PM3, first, second and third positions of the posterior midgut, respectively; and CAR, carcass (larvae less midgut).

TPM values of each gene are highlighted with different shades of green in the Tables so that one can, at a glance, know in which midgut section or carcass it is most expressed.

**Supplementary Material 14 -** Expression profile of the genes involved in arginine biosynthesis and metabolism in *Abracris flavolineata*.

| **Enzyme** | **Protein ID** | **Expression (TPM)** | | | | | |
| --- | --- | --- | --- | --- | --- | --- | --- |
|  |  | **CECA** | **SG** | **MT** | **VENT** | **ALL** | **CAR** |
| **ASS** | PQ462851 | 0.0 | 0.4 | 0.1 | 0.0 | 2.2 | 4.1 |
| **ASL** | PQ462852 | 0.3 | 0.6 | 9.8 | 0.0 | 1.1 | 1.2 |
|  | PQ462853 | 5.7 | 2.3 | 73.1 | 3.4 | 13.0 | 15.6 |
| **NOS** | PQ462854 | 0.1 | 0.2 | 0.0 | 0.3 | 0.2 | 0.3 |
|  | PQ462855 | 0.0 | 0.0 | 0.0 | 0.0 | 0.1 | 0.3 |
| **ARG** | PQ462856 | 0.0 | 0.0 | 0.0 | 0.0 | 0.2 | 0.3 |
| **OAT** | PQ462857 | 43.1 | 11.0 | 0.5 | 8.7 | 20.0 | 19.9 |
| **P5CS** | PQ462858 | 8.5 | 2.4 | 0.1 | 1.4 | 4.1 | 4.3 |
| **P5CDH** | PQ462859 | 0.4 | 0.3 | 54.9 | 0.1 | 5.4 | 5.7 |
| **PRODH** | PQ462860 | 0.1 | 0.9 | 1.0 | 0.0 | 0.6 | 0.9 |
| **PYCR** | PQ462861 | 0.0 | 0.1 | 0.5 | 0.0 | 0.3 | 0.5 |
|  | PQ462862 | 1.5 | 1.9 | 16.0 | 7.1 | 5.3 | 5.4 |
| **ODC** | PQ462863 | 3.9 | 1.7 | 1.8 | 1.8 | 2.4 | 2.3 |
|  | PQ462864 | 4.9 | 2.9 | 85.8 | 3.6 | 18.3 | 24.8 |
| **SRM** | PQ462865 | 1.0 | 1.8 | 4.2 | 0.7 | 1.5 | 1.7 |
|  | PQ462866 | 0.7 | 0.2 | 20.6 | 0.3 | 2.3 | 2.5 |
| **SMS** | PQ462867 | 0.2 | 0.1 | 4.4 | 0.1 | 0.6 | 0.7 |
| **AMD** | PQ462868 | 0.4 | 0.0 | 6.9 | 0.3 | 1.1 | 1.3 |

Expression data from RNA-sequencing (RNA-seq) [average transcript per million (TPM) values from three biological replicates], where VENT, ventriculus (tubular region of the midgut); MT, Malpighian tubules; SG, salivary gland; ALL, whole insect; CAR, carcass (adult less the previously mentioned parts, head, legs and wings).

TPM values of each gene are highlighted with different shades of green in the Tables so that one can, at a glance, know in which midgut section or carcass it is most expressed.

**Supplementary Material 15 -** Expression profile of the genes involved in arginine biosynthesis and metabolism in *Dermestes maculatus*.

| **Enzyme** | **Protein ID** | **Expression (TPM)** | | |
| --- | --- | --- | --- | --- |
|  |  | **AM** | **PM** | **CAR** |
| **ASS** | PQ462972 | 7.9 | 9.7 | 40.4 |
| **ASL** | PQ462945 | 18.6 | 28.3 | 240.3 |
|  | PQ462950 | 0.3 | 0.4 | 37.1 |
| **NOS** | PQ462960 | 8.9 | 8.1 | 1.5 |
|  | PQ462967 | 43.0 | 72.9 | 202.5 |
|  | PQ462968 | 5.5 | 5.7 | 0.1 |
|  | PQ462977 | 5.3 | 5.2 | 6.9 |
| **ARG** | PQ462943 | 0.2 | 0.1 | 45.1 |
| **OAT** | PQ462969 | 2.4 | 0.0 | 25.3 |
|  | PQ462973 | 38.7 | 45.5 | 296.3 |
|  | PQ462975 | 4.8 | 6.1 | 0.2 |
| **P5CS** | PQ462959 | 13.1 | 16.1 | 367.9 |
|  | PQ462974 | 9.4 | 8.9 | 0.3 |
| **P5CDH** | PQ462940 | 2.3 | 1.0 | 0.7 |
| **PRODH** | PQ462958 | 1.4 | 0.8 | 99.9 |
| **PYCR** | PQ462938 | 20.4 | 15.4 | 52.1 |
|  | PQ462946 | 33.5 | 37.4 | 36.6 |
|  | PQ462949 | 6.3 | 7.5 | 16.5 |
| **ODC** | PQ462976 | 158.1 | 183.3 | 1143.9 |
| **SRM** | PQ462947 | 17.7 | 17.7 | 272.9 |
|  | PQ462961 | 2.2 | 2.4 | 0.0 |
| **SMS** | PQ462952 | 7.4 | 10.2 | 18.6 |
| **AMD** | PQ462970 | 0.6 | 1.8 | 33.2 |

Expression data from RNA-sequencing (RNA-seq) [average transcript per million (TPM) values from three biological replicates], where AM and PM, anterior and posterior midgut, respectively; and CAR, carcass (larvae less midgut).

TPM values of each gene are highlighted with different shades of green in the Tables so that one can, at a glance, know in which midgut section or carcass it is most expressed.

**Supplementary Material 16 -** Expression profile of the genes involved in arginine biosynthesis and metabolism in *Dysdercus peruvianus*.

| **Enzyme** | **Protein ID** | **Expression (TPM)** | | | |
| --- | --- | --- | --- | --- | --- |
|  |  | **AM** | **MM** | **PM** | **CAR** |
| **ASS** | Not Found | | | | |
| **ASL** |  |  |  |  |  |
| **NOS** | PQ462980 | 0.6 | 0.4 | 3.0 | 30.3 |
|  | PQ462981 | 2.1 | 1.9 | 10.1 | 54.6 |
| **ARG** | PQ462982 | 0.8 | 2.1 | 3.3 | 201.6 |
| **OAT** | PQ462983 | 3.1 | 1.9 | 1.7 | 44.1 |
|  | PQ462984 | 0.1 | 0.0 | 0.0 | 182.0 |
| **P5CS** | PQ462985 | 355.8 | 372.1 | 786.5 | 306.3 |
| **P5CDH** | PQ462986 | 0.0 | 0.0 | 0.0 | 0.0 |
|  | PQ462987 | 2.1 | 1.6 | 6.0 | 84.3 |
| **PRODH** | PQ462988 | 0.7 | 0.8 | 2.8 | 17.9 |
|  | PQ462989 | 6.3 | 5.3 | 16.0 | 32.2 |
|  | PQ462990 | 4.0 | 3.1 | 10.4 | 25.5 |
| **PYCR** | PQ462991 | 29.4 | 34.0 | 35.5 | 32.1 |
| **ODC** | PQ462992 | 0.1 | 0.0 | 0.4 | 136.0 |
|  | PQ462993* | 828.0 | 1,180.6 | 1,579.0 | 672.1 |
| **SRM** | PQ462994 | 0.5 | 0.5 | 0.8 | 312.9 |
| **SMS** | PQ462995 | 3.0 | 3.0 | 6.4 | 15.8 |
| **AMD** | PQ462996 | 0.2 | 0.2 | 0.5 | 59.5 |

Expression data from RNA-sequencing (RNA-seq) [average transcript per million (TPM) values from three biological replicates], where AM, MM, and PM, anterior, middle and posterior midgut; and CAR, carcass (adult less midgut, head, legs and wings).

TPM values of each gene are highlighted with different shades of green in the Tables so that one can, at a glance, know in which midgut section or carcass it is most expressed.

**Supplementary Material 17 –** Expression profile of the genes involved in arginine biosynthesis and metabolism in *Mahanarva fimbriolata*.

| **Enzyme** | **Protein ID** | **Expression (TPM)** | | | | |
| --- | --- | --- | --- | --- | --- | --- |
|  |  | **FC** | **AM** | **MM** | **PM** | **CAR** |
| **ASS** | Not found | | | | | |
| **ASL** |  |  |  |  |  |  |
| **NOS** | PQ462885 | 6.5 | 8.6 | 6.6 | 6.7 | 8.2 |
| **ARG** | PQ462886 | 0.7 | 0.5 | 0.6 | 10.2 | 22.2 |
| **OAT** | PQ462887 | 3.3 | 5.0 | 5.5 | 4.4 | 3.8 |
|  | PQ462888 | 0.1 | 0.1 | 0.2 | 3.4 | 23.2 |
|  | PQ462889 | 2.1 | 2.8 | 2.3 | 10.1 | 22.9 |
| **P5CS** | PQ462890 | 0.9 | 7.1 | 2.2 | 5.9 | 5.8 |
|  | PQ462891 | 72.4 | 71.5 | 117.4 | 16.1 | 6.5 |
| **P5CDH** | PQ462892 | 0.7 | 0.9 | 0.8 | 2.8 | 4.4 |
| **PRODH** | PQ462893 | 0.0 | 3.3 | 0.2 | 1.5 | 2.0 |
|  | PQ462894 | 3.7 | 3.6 | 3.9 | 9.6 | 28.0 |
| **PYCR** | PQ462895 | 1.8 | 2.1 | 3.4 | 0.4 | 0.3 |
|  | PQ462896 | 55.1 | 34.2 | 45.9 | 15.8 | 4.6 |
|  | PQ462897 | 9.2 | 13.7 | 11.6 | 9.6 | 5.4 |
|  | PQ462898 | 1.2 | 2.0 | 1.6 | 1.2 | 0.6 |
|  | PQ462899 | 2.8 | 2.8 | 10.6 | 6.2 | 0.7 |
| **ODC** | PQ462900 | 10.1 | 25.7 | 21.2 | 19.4 | 24.0 |
|  | PQ462901 | 218.8 | 115.0 | 91.5 | 169.0 | 44.7 |
| **SRM** | PQ462902 | 1.5 | 4.6 | 1.5 | 5.8 | 4.1 |
|  | PQ462903 | 16.2 | 15.8 | 15.4 | 12.9 | 16.8 |
| **SMS** | PQ462904 | 1.3 | 0.8 | 2.0 | 1.0 | 3.5 |
| **AMD** | PQ462905 | 0.4 | 0.3 | 1.8 | 0.0 | 1.6 |
|  | PQ462906 | 135.1 | 77.2 | 71.1 | 61.2 | 29.5 |
|  | PQ462907 | 119.5 | 56.4 | 52.7 | 41.1 | 21.1 |

Expression data from RNA-sequencing (RNA-seq) [average transcript per million (TPM) values from three biological replicates], where FC, filter chamber; AM, MM, and PM, anterior, middle and posterior midgut, respectively; and CAR, carcass (adult less midgut, head, legs and wings).

TPM values of each gene are highlighted with different shades of green in the Tables so that one can, at a glance, know in which midgut section or carcass it is most expressed.

**Supplementary Material 18 –** Sum of the TPM values of all genes coding for urea cycle enzymes in the insects analyzed.

| Species | Sample | ASS | ASL | NOS | ARG |
| --- | --- | --- | --- | --- | --- |
| *Periplaneta americana* | # genes | 2 | 4 | 2 | 2 |
|  | GC | 1.0 | 0.0 | 0.1 | 0.2 |
|  | SG | 0.9 | 0.0 | 4.5 | 1.6 |
|  | A | 0.0 | 0.0 | 0.1 | 0.2 |
|  | M1 | 0.0 | 0.0 | 1.4 | 0.7 |
|  | M2 | 0.6 | 0.0 | 1.7 | 1.1 |
|  | P | 9.4 | 0.0 | 0.8 | 2.5 |
|  | MT | 271.8 | 0.0 | 0.0 | 6.8 |
|  | CAR | 99.9 | 0.0 | 1.9 | 41.0 |
| *Rhodnius prolixus* | # genes | 0 | 0 | 2 | 0 |
|  | A | NF | NF | 0.20 | NF |
|  | M | NF | NF | 0.80 | NF |
|  | P | NF | NF | 0.20 | NF |
|  | CAR | NF | NF | 7.40 | NF |
| *Tenebrio molitor* | # genes | 1 | 1 | 1 | 1 |
|  | A | 19.3 | 0.5 | 178.4 | 0.6 |
|  | M | 15.4 | 0.1 | 106.7 | 0.2 |
|  | P | 14.6 | 0.7 | 38.3 | 0.4 |
|  | CAR | 79.9 | 70.5 | 6.2 | 33.9 |
| *Musca domestica* | # genes | 1 | 1 | 2 | 1 |
|  | A1 | 0.2 | 0.4 | 0.0 | 0.2 |
|  | A2 | 0.1 | 0.1 | 0.0 | 0.1 |
|  | M1 | 1.9 | 0.7 | 0.0 | 1.2 |
|  | M2 | 0.0 | 0.4 | 0.0 | 0.7 |
|  | P1 | 0.0 | 0.1 | 0.0 | 0.4 |
|  | P2 | 1.0 | 0.7 | 0.0 | 0.3 |
|  | P3 | 1.8 | 1.4 | 0.1 | 0.4 |
|  | CAR | 99.3 | 53.8 | 1.0 | 69.3 |
| *Spodoptera frugiperda* | # genes | 1 | 1 | 2 | 1 |
|  | A | 27.9 | 2.3 | 3.0 | 1.6 |
|  | M | 7.3 | 0.8 | 1.4 | 0.2 |
|  | P | 0.9 | 0.4 | 1.9 | 0.1 |
|  | CAR | 511.7 | 31.4 | 14.6 | 26.7 |
| *Abracris flavolineata* | # genes | 1 | 2 | 2 | 1 |
|  | Ceccum | 0.0 | 6.0 | 0.1 | 0.0 |
|  | Salivar Gland | 0.4 | 2.9 | 0.2 | 0.0 |
|  | Malpighi | 0.1 | 82.0 | 0.0 | 0.0 |
|  | Ventriculo | 0.0 | 3.4 | 0.3 | 0.0 |
|  | All | 2.2 | 14.1 | 0.3 | 0.2 |
|  | Car | 4.1 | 16.8 | 0.6 | 0.3 |
| *Dermestes maculatus* | #genes | 1 | 2 | 4 | 1 |
|  | A | 7.9 | 18.8 | 62.6 | 0.2 |
|  | IMP | 9.7 | 28.7 | 91.9 | 0.1 |
|  | Car | 40.4 | 277.4 | 210.0 | 45.1 |
| *Dysdercus peruvianus* | #genes | 0 | 0 | 2 | 1 |
|  | A | NF | NF | 2.7 | 0.8 |
|  | M | NF | NF | 2.3 | 2.1 |
|  | P | NF | NF | 13.2 | 3.3 |
|  | CAR | NF | NF | 84.8 | 201.6 |
| *Mahanarva fimbriolata* | #genes | 0 | 0 | 1 | 6 |
|  | CF | NF | NF | 6.5 | 0.7 |
|  | DC | NF | NF | 8.6 | 0.5 |
|  | DTA | NF | NF | 6.6 | 0.6 |
|  | DTP | NF | NF | 6.7 | 10.2 |
|  | CAR | NF | NF | 8.2 | 22.2 |

**Supplementary Material 19:** Sum of the TPM values of all genes coding for enzymes involved in ornithine biosynthesis and metabolism.

| Species | Sample | PRODH | PYCR | P5CS | P5CDH | OAT | ODC | ARG |
| --- | --- | --- | --- | --- | --- | --- | --- | --- |
| *Periplaneta americana* | # genes | 1 | 2 | 2 | 1 | 1 | 3 | 2 |
|  | GC | 1.8 | 11.4 | 0.2 | 42.4 | 39.3 | 30.4 | 0.2 |
|  | SG | 31.7 | 16.4 | 2.6 | 46.1 | 8.2 | 39.7 | 1.6 |
|  | A | 23.2 | 21.1 | 1.2 | 44.0 | 2.4 | 69.4 | 0.2 |
|  | M1 | 3.2 | 14.0 | 0.0 | 63.4 | 2.2 | 43.6 | 0.7 |
|  | M2 | 7.5 | 25.7 | 0.0 | 82.6 | 1.8 | 78.8 | 1.1 |
|  | P | 62.8 | 34.4 | 2.1 | 133.8 | 23.1 | 104.1 | 2.5 |
|  | MT | 17.8 | 29.0 | 5.8 | 210.3 | 39.7 | 98.9 | 6.8 |
|  | CAR | 26.6 | 60.4 | 37.2 | 55.2 | 125.6 | 102.2 | 41.0 |
| *Rhodnius prolixus* | # genes | 3 | 3 | 1 | 1 | 2 | 1 | 0 |
|  | A | 1.8 | 224.6 | 11.0 | 1.6 | 14.7 | 58.7 | NF |
|  | M | 2.2 | 998.3 | 1.7 | 3.8 | 32.1 | 61.6 | NF |
|  | P | 1.6 | 591.3 | 0.9 | 5.5 | 19.1 | 58.1 | NF |
|  | CAR | 19.4 | 1145.0 | 92.4 | 49.5 | 48.4 | 274.1 | NF |
| *Tenebrio molitor* | # genes | 1 | 2 | 1 | 1 | 1 | 1 | 1 |
|  | A | 0.5 | 55.9 | 152.4 | 9.3 | 154.9 | 92.8 | 0.6 |
|  | M | 0.5 | 38.1 | 119.4 | 8.5 | 169.0 | 79.1 | 0.2 |
|  | P | 1.0 | 35.3 | 90.9 | 9.1 | 77.0 | 74.5 | 0.4 |
|  | CAR | 52.3 | 128.8 | 157.6 | 110.3 | 230.9 | 207.2 | 33.9 |
| *Musca domestica* | # genes | 1 | 2 | 3 | 2 | 1 | 3 | 1 |
|  | A1 | 0.7 | 16.0 | 33.5 | 1.8 | 6.4 | 0.1 | 0.2 |
|  | A2 | 0.2 | 18.4 | 36.0 | 0.5 | 7.2 | 0.1 | 0.1 |
|  | M1 | 0.7 | 36.5 | 265.5 | 1.2 | 13.1 | 0.4 | 1.2 |
|  | M2 | 1.0 | 60.0 | 264.6 | 1.7 | 17.3 | 0.1 | 0.7 |
|  | P1 | 0.9 | 19.9 | 8.4 | 0.8 | 28.4 | 0.0 | 0.4 |
|  | P2 | 0.8 | 30.5 | 3.3 | 1.2 | 62.5 | 0.1 | 0.3 |
|  | P3 | 2.3 | 51.0 | 18.2 | 2.4 | 52.9 | 56.7 | 0.4 |
|  | CAR | 9.8 | 49.9 | 203.2 | 21.3 | 182.7 | 40.8 | 69.3 |
| *Spodoptera frugiperda* | # genes | 2 | 2 | 1 | 2 | 1 | 1 | 1 |
|  | A | 2.7 | 76.9 | 70.9 | 12.7 | 10.1 | 51.1 | 1.6 |
|  | M | 1.6 | 387.1 | 29.6 | 6.2 | 354.6 | 89.4 | 0.2 |
|  | P | 3.0 | 302.8 | 297.2 | 7.5 | 244.1 | 90.5 | 0.1 |
|  | CAR | 15.1 | 266.5 | 104.2 | 74.2 | 59.2 | 56.6 | 26.7 |
| *Abracris flavolineata* | # genes | 1 | 2 | 1 | 1 | 1 | 2 | 1 |
|  | Ceccum | 0.1 | 1.5 | 8.5 | 0.4 | 43.1 | 8.8 | 0.0 |
|  | Salivar Gland | 0.9 | 1.9 | 2.4 | 0.3 | 11.0 | 4.5 | 0.0 |
|  | Malpighi | 1.0 | 16.5 | 0.1 | 54.9 | 0.5 | 87.6 | 0.0 |
|  | Ventriculo | 0.0 | 7.1 | 1.4 | 0.1 | 8.7 | 5.4 | 0.0 |
|  | All | 0.6 | 5.6 | 4.1 | 5.4 | 20.0 | 20.8 | 0.2 |
|  | Car | 0.9 | 5.9 | 4.3 | 5.7 | 19.9 | 27.1 | 0.3 |
| *Dermestes maculatus* | #genes | 1 | 3 | 2 | 1 | 3 | 1 | 1 |
|  | A | 1.4 | 60.2 | 22.5 | 2.3 | 45.9 | 158.1 | 0.2 |
|  | IMP | 0.8 | 60.2 | 25.0 | 1.0 | 51.6 | 183.3 | 0.1 |
|  | Car | 99.9 | 105.2 | 368.3 | 0.7 | 321.8 | 1,143.9 | 45.1 |
| *Dysdercus peruvianus* | #genes | 3 | 1 | 1 | 2 | 2 | 2 | 1 |
|  | A | 11 | 29.4 | 355.8 | 2.1 | 3.1 | 828.1 | 0.8 |
|  | M | 9.2 | 34.0 | 372.1 | 1.6 | 1.9 | 1,180.6 | 2.1 |
|  | P | 29.3 | 35.5 | 786.5 | 6.0 | 1.7 | 1,579.5 | 3.3 |
|  | CAR | 75.6 | 32.1 | 306.3 | 84.3 | 226.2 | 808.1 | 201.6 |
| *Mahanarva fimbriolata* | #genes | 2 | 5 | 2 | 1 | 3 | 2 | 1 |
|  | CF | 3.7 | 70.1 | 73.3 | 0.7 | 5.5 | 228.9 | 0.7 |
|  | DC | 6.9 | 54.8 | 78.6 | 0.9 | 7.9 | 140.7 | 0.5 |
|  | DTA | 4.1 | 73.2 | 119.6 | 0.8 | 8.0 | 112.6 | 0.6 |
|  | DTP | 11.0 | 33.3 | 22.0 | 2.8 | 17.9 | 188.4 | 10.2 |
|  | CAR | 30.0 | 11.6 | 12.3 | 4.4 | 49.9 | 68.8 | 22.2 |

**Supplementary Material 20 –** Sum of the TPM values of all genes coding for enzymes involved in spermine biosynthesis and metabolism.

| Species | Sample | ODC | SRM | SMS | AMD |
| --- | --- | --- | --- | --- | --- |
| *Periplaneta americana* | # genes | 3 | 2 | 1 | 1 |
|  | GC | 30.4 | 5.0 | 1.4 | 8.3 |
|  | SG | 39.7 | 1.5 | 1.2 | 10.8 |
|  | HG | 69.4 | 2.2 | 0.0 | 8.0 |
|  | AM | 43.6 | 4.6 | 6.2 | 13.1 |
|  | PM | 78.8 | 9.3 | 5.3 | 21.2 |
|  | FG | 104.1 | 5.3 | 4.3 | 20.5 |
|  | MT | 98.9 | 3.0 | 10.3 | 35.1 |
|  | CAR | 102.2 | 10.6 | 2.3 | 9.1 |
| *Rhodnius prolixus* | # genes | 1 | 1 | 1 | 1 |
|  | AM | 58.7 | 11.5 | 11.8 | 0.7 |
|  | MM | 61.6 | 2.2 | 5.6 | 2.60 |
|  | PM | 58.1 | 3.1 | 3.6 | 1.7 |
|  | CAR | 274.1 | 45.9 | 17.9 | 10.6 |
| *Tenebrio molitor* | # genes | 1 | 1 | 1 | 1 |
|  | AM | 92.8 | 7.1 | 33.8 | 15.8 |
|  | MM | 79.1 | 6.7 | 28.1 | 12.2 |
|  | PM | 74.5 | 6.8 | 29.5 | 12.1 |
|  | CAR | 207.2 | 53.8 | 98.5 | 20.1 |
| *Musca domestica* | # genes | 3 | 1 | 1 | 1 |
|  | AM1 | 0.1 | 0.0 | 0.1 | 1.2 |
|  | AM2 | 0.1 | 0.0 | 0.0 | 1.1 |
|  | MM1 | 0.4 | 0.0 | 0.3 | 2.5 |
|  | MM2 | 0.1 | 0.0 | 0.3 | 3.3 |
|  | PM1 | 0.0 | 0.0 | 0.2 | 1.9 |
|  | PM2 | 0.1 | 0.0 | 0.3 | 2.5 |
|  | PM3 | 56.7 | 0.0 | 0.5 | 3.8 |
|  | CAR | 40.8 | 0.0 | 16.3 | 10.8 |
| *Spodoptera frugiperda* | # genes | 1 | 1 | 1 | 1 |
|  | AM | 51.1 | 7.3 | 5.1 | 17.6 |
|  | MM | 89.4 | 5.7 | 7.0 | 14.6 |
|  | PM | 90.5 | 5.7 | 6.0 | 11.6 |
|  | CAR | 56.6 | 13.1 | 12.2 | 110.1 |
| *Abracris flavolineata* | # genes | 2 | 2 | 1 | 1 |
|  | CECA | 8.8 | 1.7 | 0.2 | 0.4 |
|  | SG | 4.5 | 2.0 | 0.1 | 0.0 |
|  | MT | 87.6 | 24.8 | 4.4 | 6.9 |
|  | VENT | 5.4 | 1.1 | 0.1 | 0.3 |
|  | ALL | 20.8 | 3.9 | 0.6 | 1.1 |
|  | CAR | 27.1 | 4.2 | 0.7 | 1.3 |
| *Dermestes maculatus* | #genes | 1 | 2 | 1 | 1 |
|  | AM | 158.1 | 19.9 | 7.4 | 0.6 |
|  | PM | 183.3 | 20.1 | 10.2 | 1.8 |
|  | CAR | 1143.9 | 272.9 | 18.6 | 33.2 |
| *Dysdercus peruvianus* | #genes | 2 | 1 | 1 | 1 |
|  | AM | 828.1 | 0.5 | 3.0 | 0.2 |
|  | MM | 1,180.6 | 0.5 | 3.0 | 0.2 |
|  | PM | 1,579.5 | 0.8 | 6.4 | 0.5 |
|  | CAR | 808.1 | 312.9 | 15.8 | 59.5 |
| *Mahanarva fimbriolata* | #genes | 2 | 2 | 1 | 3 |
|  | FC | 228.9 | 17.6 | 1.3 | 254.9 |
|  | DC | 140.7 | 20.4 | 0.8 | 133.8 |
|  | DTA | 112.6 | 17.0 | 2.0 | 125.6 |
|  | DTP | 188.4 | 18.7 | 1.0 | 102.2 |
|  | CAR | 68.8 | 20.9 | 3.5 | 52.2 |

**Supplementary Material 21** - Summary of species analyzed, including NCBI TaxID, source database, accession number, and BUSCO performance. BUSCO statistics are given as percentages, detailing the proportions of Complete, Single-copy, Duplicated, Fragmented, and Missing genes BUSCO marker genes for each species.

| **Species** | **NCBI’s TaxID** | **Database** | **Accession number** | **BUSCO statistics (%)** | | | | |
| --- | --- | --- | --- | --- | --- | --- | --- | --- |
|  |  |  |  | **Completed** | **Single-copy** | **Duplicated** | **Fragmented** | **Missing** |
| *Abscondita terminalis* | 2069292 | NCBI GenBank | GCA_013368085.1 | 96.1 | 92.9 | 3.2 | 1.2 | 2.7 |
| *Acromyrmex echinatior* | 103372 | NCBI RefSeq | GCF_000204515.1 | 99.6 | 99.1 | 0.5 | 0.1 | 0.3 |
| *Acyrthosiphon pisum* | 7029 | NCBI RefSeq | GCF_005508785.1 | 97.8 | 91.2 | 6.6 | 0.4 | 1.8 |
| *Aedes aegypti* | 7159 | NCBI RefSeq | GCF_002204515.2 | 99.4 | 93.5 | 5.9 | 0.1 | 0.5 |
| *Aethina tumida* | 116153 | NCBI RefSeq | GCF_001937115.1 | 99.4 | 88.4 | 11 | 0.3 | 0.3 |
| *Agrilus planipennis* | 224129 | NCBI RefSeq | GCF_000699045.2 | 97.8 | 90.4 | 7.4 | 1 | 1.2 |
| *Ampulex compressa* | 860918 | NCBI GenBank | GCA_019049445.1 | 97.1 | 97 | 0.1 | 1.6 | 1.3 |
| *Amyelois transitella* | 680683 | NCBI RefSeq | GCF_001186105.1 | 99.5 | 96.9 | 2.6 | 0.2 | 0.3 |
| *Anopheles albimanus* | 7167 | NCBI RefSeq | GCF_013758885.1 | 99.3 | 98.2 | 1.1 | 0.2 | 0.5 |
| *Anoplophora glabripennis* | 217634 | NCBI RefSeq | GCF_000390285.2 | 99.5 | 97.5 | 2 | 0.3 | 0.2 |
| *Aphidius gifuensis* | 684658 | NCBI RefSeq | GCF_014905175.1 | 98.6 | 94.5 | 4.1 | 0.2 | 1.2 |
| *Aphis craccivora* | 307492 | NCBI GenBank | GCA_009835225.1 | 95.3 | 92.8 | 2.5 | 2.3 | 2.4 |
| *Apis laboriosa* | 183418 | NCBI RefSeq | GCF_014066325.1 | 99.8 | 99.7 | 0.1 | 0.1 | 0.1 |
| *Apolygus lucorum* | 248454 | NCBI GenBank | GCA_009739505.2 | 94.1 | 89.6 | 4.5 | 1.7 | 4.2 |
| *Arctia plantaginis* | 874455 | NCBI GenBank | GCA_902825455.1 | 96.5 | 95.6 | 0.9 | 0.5 | 3 |
| *Aricia agestis* | 91739 | NCBI RefSeq | GCF_905147365.1 | 99.9 | 99.6 | 0.3 | 0 | 0.1 |
| *Athalia rosae* | 37344 | NCBI RefSeq | GCF_000344095.2 | 99.5 | 99.4 | 0.1 | 0 | 0.5 |
| *Atta colombica* | 520822 | NCBI RefSeq | GCF_001594045.1 | 99.3 | 98.7 | 0.6 | 0.3 | 0.4 |
| *Bactrocera latifrons* | 174628 | NCBI RefSeq | GCF_001853355.1 | 99.5 | 98.6 | 0.9 | 0.1 | 0.4 |
| *Belonocnema treatae* | 1159321 | NCBI RefSeq | GCF_010883055.1 | 97.5 | 96.8 | 0.7 | 1.5 | 1 |
| *Bemisia tabaci* | 7038 | NCBI RefSeq | GCF_001854935.1 | 98.9 | 96.9 | 2 | 0.1 | 1 |
| *Bicyclus anynana* | 110368 | NCBI RefSeq | GCF_900239965.1 | 99.3 | 98.2 | 1.1 | 0.6 | 0.1 |
| *Blattella germanica* | 6973 | NCBI GenBank | GCA_003018175.1 | 84.6 | 82.8 | 1.8 | 9.2 | 6.2 |
| *Bombus terrestris* | 30195 | NCBI RefSeq | GCF_000214255.1 | 99.7 | 99.6 | 0.1 | 0.1 | 0.2 |
| *Bombyx mori* | 7091 | NCBI RefSeq | GCF_014905235.1 | 99.5 | 98.1 | 1.4 | 0.2 | 0.3 |
| *Bradysia coprophila* | 38358 | NCBI RefSeq | GCF_014529535.1 | 99.9 | 96.3 | 3.6 | 0 | 0.1 |
| *Brassicogethes aeneus* | 1431903 | NCBI GenBank | GCA_921294245.1 | 96.1 | 94.2 | 1.9 | 0.9 | 3 |
| *Brenthis ino* | 405034 | NCBI GenBank | GCA_921882275.1 | 98.4 | 98.1 | 0.3 | 1 | 0.6 |
| *Camponotus floridanus* | 104421 | NCBI RefSeq | GCF_003227725.1 | 99.2 | 98.3 | 0.9 | 0.2 | 0.6 |
| *Catajapyx aquilonaris* | 438503 | i5K | unavailable | 89.2 | 88.7 | 0.5 | 5.8 | 5 |
| *Centruroides sculpturatus* | 218467 | NCBI RefSeq | GCF_000671375.1 | 93.5 | 88.2 | 5.3 | 4.1 | 2.4 |
| *Cephus cinctus* | 211228 | NCBI RefSeq | GCF_000341935.1 | 99.6 | 99.2 | 0.4 | 0.1 | 0.3 |
| *Ceratina calcarata* | 156304 | NCBI RefSeq | GCF_001652005.1 | 98.2 | 95.5 | 2.7 | 1 | 0.8 |
| *Ceratitis capitata* | 7213 | NCBI RefSeq | GCF_000347755.3 | 99.8 | 99.4 | 0.4 | 0 | 0.2 |
| *Ceratosolen solmsi marchali* | 326594 | NCBI RefSeq | GCF_000503995.1 | 97.5 | 96.8 | 0.7 | 1 | 1.5 |
| *Chelonus insularis* | 460826 | NCBI RefSeq | GCF_013357705.1 | 99.4 | 98.6 | 0.8 | 0 | 0.6 |
| *Chilo suppressalis* | 168631 | NCBI GenBank | GCA_004000445.1 | 93 | 90.5 | 2.5 | 2.6 | 4.4 |
| *Chrysoperla carnea* | 189513 | NCBI RefSeq | GCF_905475395.1 | 98 | 97.2 | 0.8 | 0.1 | 1.9 |
| *Cimex lectularius* | 79782 | NCBI RefSeq | GCF_000648675.2 | 99.5 | 98.4 | 1.1 | 0.2 | 0.3 |
| *Clunio marinus* | 568069 | NCBI GenBank | GCA_900005825.1 | 94.6 | 93.6 | 1 | 0.7 | 4.7 |
| *Coccinella septempunctata* | 41139 | NCBI RefSeq | GCF_907165205.1 | 99.7 | 98.4 | 1.3 | 0 | 0.3 |
| *Colias croceus* | 72248 | NCBI RefSeq | GCF_905220415.1 | 99.9 | 99.5 | 0.4 | 0 | 0.1 |
| *Colletes gigas* | 935657 | NCBI RefSeq | GCF_013123115.1 | 99.5 | 98.2 | 1.3 | 0.1 | 0.4 |
| *Contarinia nasturtii* | 265458 | NCBI RefSeq | GCF_009176525.2 | 99.5 | 97.8 | 1.7 | 0.1 | 0.4 |
| *Copidosoma floridanum* | 29053 | NCBI RefSeq | GCF_000648655.2 | 98.3 | 95.3 | 3 | 0.6 | 1.1 |
| *Coptotermes formosanus* | 36987 | NCBI GenBank | GCA_013340265.1 | 90 | 88.6 | 1.4 | 5.4 | 4.6 |
| *Cotesia glomerata* | 32391 | NCBI RefSeq | GCF_020080835.1 | 99.4 | 97.8 | 1.6 | 0 | 0.6 |
| *Cryptotermes secundus* | 105785 | NCBI RefSeq | GCF_002891405.2 | 98.3 | 96.9 | 1.4 | 0.4 | 1.3 |
| *Culex pipiens pallens* | 42434 | NCBI RefSeq | GCF_016801865.1 | 95.1 | 90.1 | 5 | 0.7 | 4.2 |
| *Culex quinquefasciatus* | 7176 | NCBI RefSeq | GCF_015732765.1 | 96.3 | 95.5 | 0.8 | 0.3 | 3.4 |
| *Cyphomyrmex costatus* | 456900 | NCBI RefSeq | GCF_001594065.1 | 99.3 | 98.5 | 0.8 | 0.2 | 0.5 |
| *Daktulosphaira vitifoliae* | 58002 | AphidBase (BIPAA) | unavailable | 96.1 | 93.3 | 2.8 | 1.2 | 2.7 |
| *Danaus plexippus plexippus* | 278856 | NCBI RefSeq | GCF_009731565.1 | 99.1 | 96.9 | 2.2 | 0.6 | 0.3 |
| *Daphnia magna* | 35525 | NCBI RefSeq | GCF_003990815.1 | 97.6 | 95.5 | 2.1 | 0.4 | 2 |
| *Darwinula stevensoni* | 69355 | NCBI GenBank | GCA_905338385.1 | 84.2 | 82.2 | 2 | 4.2 | 11.6 |
| *Dendroctonus ponderosae* | 77166 | NCBI RefSeq | GCF_000355655.1 | 98.5 | 92.8 | 5.7 | 0.6 | 0.9 |
| *Dermatophagoides pteronyssinus* | 6956 | NCBI RefSeq | GCF_001901225.1 | 91.9 | 90.5 | 1.4 | 1.2 | 6.9 |
| *Diabrotica virgifera virgifera* | 50390 | NCBI RefSeq | GCF_003013835.1 | 96.6 | 94.6 | 2 | 1.9 | 1.5 |
| *Diachasma alloeum* | 454923 | NCBI RefSeq | GCF_001412515.2 | 99.1 | 98.7 | 0.4 | 0 | 0.9 |
| *Dinoponera quadriceps* | 609295 | NCBI RefSeq | GCF_001313825.1 | 99.1 | 98.3 | 0.8 | 0.5 | 0.4 |
| *Diprion similis* | 362088 | NCBI RefSeq | GCF_021155765.1 | 96.1 | 95.6 | 0.5 | 0.3 | 3.6 |
| *Diuraphis noxia* | 143948 | NCBI RefSeq | GCF_001186385.1 | 92.8 | 90.8 | 2 | 3.1 | 4.1 |
| *Drosophila guanche* | 7266 | NCBI RefSeq | GCF_900245975.1 | 99.7 | 98.6 | 1.1 | 0.1 | 0.2 |
| *Drosophila melanogaster* | 7227 | NCBI RefSeq | GCF_000001215.4 | 99.9 | 99.1 | 0.8 | 0 | 0.1 |
| *Drosophila pseudoobscura* | 7237 | NCBI RefSeq | GCF_009870125.1 | 99.7 | 98.5 | 1.2 | 0.1 | 0.2 |
| *Drosophila takahashii* | 29030 | NCBI RefSeq | GCF_018152695.1 | 98.7 | 97.4 | 1.3 | 0.1 | 1.2 |
| *Drosophila virilis* | 7244 | NCBI RefSeq | GCF_003285735.1 | 99.8 | 96.7 | 3.1 | 0 | 0.2 |
| *Dufourea novaeangliae* | 178035 | NCBI RefSeq | GCF_001272555.1 | 99.7 | 99.6 | 0.1 | 0.1 | 0.2 |
| *Eciton burchellii* | 213866 | NCBI GenBank | GCA_020341155.1 | 99.2 | 98.3 | 0.9 | 0.3 | 0.5 |
| *Eriosoma lanigerum* | 133082 | AphidBase (BIPAA) | unavailable | 96.6 | 93.7 | 2.9 | 1.8 | 1.6 |
| *Eufriesea mexicana* | 516756 | NCBI RefSeq | GCF_001483705.1 | 98.4 | 96.8 | 1.6 | 0.7 | 0.9 |
| *Eurytemora affinis* | 88015 | NCBI RefSeq | GCF_000591075.1 | 93.6 | 91.3 | 2.3 | 2.8 | 3.6 |
| *Folsomia candida* | 158441 | NCBI RefSeq | GCF_002217175.1 | 97.9 | 96.1 | 1.8 | 0.4 | 1.7 |
| *Fopius arisanus* | 64838 | NCBI RefSeq | GCF_000806365.1 | 98.4 | 98.2 | 0.2 | 0.6 | 1 |
| *Formica exsecta* | 72781 | NCBI RefSeq | GCF_003651465.1 | 99.4 | 95.4 | 4 | 0.3 | 0.3 |
| *Frankliniella occidentalis* | 133901 | NCBI RefSeq | GCF_000697945.2 | 98.8 | 96.9 | 1.9 | 0.5 | 0.7 |
| *Frieseomelitta varia* | 561572 | NCBI RefSeq | GCF_011392965.1 | 98.8 | 98.5 | 0.3 | 0.4 | 0.8 |
| *Galleria mellonella* | 7137 | NCBI RefSeq | GCF_003640425.2 | 92.4 | 91.9 | 0.5 | 4.6 | 3 |
| *Glossina fuscipes* | 7396 | NCBI RefSeq | GCF_014805625.1 | 99.7 | 96.6 | 3.1 | 0.2 | 0.1 |
| *Gonioctena quinquepunctata* | 63699 | NCBI GenBank | GCA_018342105.1 | 92.6 | 91 | 1.6 | 4.1 | 3.3 |
| *Habropoda laboriosa* | 597456 | NCBI RefSeq | GCF_001263275.1 | 99.1 | 98.9 | 0.2 | 0.4 | 0.5 |
| *Halyomorpha halys* | 286706 | NCBI RefSeq | GCF_000696795.2 | 99 | 97.1 | 1.9 | 0.4 | 0.6 |
| *Harpegnathos saltator* | 610380 | NCBI RefSeq | GCF_003227715.1 | 99.8 | 98.4 | 1.4 | 0.2 | 0 |
| *Helicoverpa zea* | 7113 | NCBI RefSeq | GCF_022581195.2 | 99.9 | 99.3 | 0.6 | 0 | 0.1 |
| *Hermetia illucens* | 343691 | NCBI RefSeq | GCF_905115235.1 | 99.4 | 99 | 0.4 | 0.1 | 0.5 |
| *Homarus americanus* | 6706 | NCBI RefSeq | GCF_018991925.1 | 97.5 | 96.1 | 1.4 | 1.4 | 1.1 |
| *Hyalella azteca* | 294128 | NCBI RefSeq | GCF_000764305.1 | 95.4 | 94.4 | 1 | 2 | 2.6 |
| *Hyposmocoma kahamanoa* | 1477025 | NCBI RefSeq | GCF_003589595.1 | 93 | 91.9 | 1.1 | 2.2 | 4.8 |
| *Ignelater luminosus* | 2038154 | NCBI GenBank | GCA_011009095.1 | 94.5 | 92.3 | 2.2 | 2.5 | 3 |
| *Ischnura elegans* | 197161 | NCBI RefSeq | GCF_921293095.1 | 99.1 | 98.5 | 0.6 | 0 | 0.9 |
| *Ixodes scapularis* | 6945 | NCBI RefSeq | GCF_016920785.2 | 98.5 | 93.8 | 4.7 | 0.6 | 0.9 |
| *Lamprigera yunnana* | 370605 | NCBI GenBank | GCA_013368075.1 | 94.7 | 92.8 | 1.9 | 1.9 | 3.4 |
| *Laodelphax striatellus* | 195883 | i5K | unavailable | 94.8 | 90.7 | 4.1 | 1.9 | 3.3 |
| *Lepeophtheirus salmonis* | 72036 | NCBI RefSeq | GCF_016086655.2 | 96 | 92.5 | 3.5 | 0.8 | 3.2 |
| *Leptinotarsa decemlineata* | 7539 | NCBI RefSeq | GCF_000500325.1 | 96 | 94.8 | 1.2 | 1.9 | 2.1 |
| *Leptopilina heterotoma* | 63436 | NCBI RefSeq | GCF_015476425.1 | 99.5 | 99 | 0.5 | 0 | 0.5 |
| *Limulus polyphemus* | 6850 | NCBI RefSeq | GCF_000517525.1 | 93.7 | 79.9 | 13.8 | 4.3 | 2 |
| *Linepithema humile* | 83485 | NCBI RefSeq | GCF_000217595.1 | 99.3 | 98.2 | 1.1 | 0.1 | 0.6 |
| *Lucilia cuprina* | 7375 | NCBI RefSeq | GCF_000699065.1 | 99.2 | 98.7 | 0.5 | 0.5 | 0.3 |
| *Manduca sexta* | 7130 | NCBI RefSeq | GCF_014839805.1 | 99.3 | 89.2 | 10.1 | 0.2 | 0.5 |
| *Maniola jurtina* | 191418 | NCBI RefSeq | GCF_905333055.1 | 99.5 | 98.3 | 1.2 | 0.1 | 0.4 |
| *Megachile rotundata* | 143995 | NCBI RefSeq | GCF_000220905.1 | 99.3 | 99.2 | 0.1 | 0.1 | 0.6 |
| *Megalopta genalis* | 115081 | NCBI RefSeq | GCF_011865705.1 | 98.4 | 95.9 | 2.5 | 0 | 1.6 |
| *Melanaphis sacchari* | 742174 | NCBI RefSeq | GCF_002803265.2 | 98.2 | 96 | 2.2 | 0.5 | 1.3 |
| *Melipona quadrifasciata* | 166423 | NCBI GenBank | GCA_001276565.1 | 91.7 | 91.2 | 0.5 | 1.1 | 7.2 |
| *Microplitis demolitor* | 69319 | NCBI RefSeq | GCF_000572035.2 | 99.5 | 98 | 1.5 | 0.1 | 0.4 |
| *Monomorium pharaonis* | 307658 | NCBI RefSeq | GCF_013373865.1 | 99.6 | 95.8 | 3.8 | 0.2 | 0.2 |
| *Musca domestica* | 7370 | NCBI RefSeq | GCF_000371365.1 | 99.4 | 96.4 | 3 | 0.2 | 0.4 |
| *Myzus persicae* | 13164 | NCBI RefSeq | GCF_001856785.1 | 98.3 | 94.8 | 3.5 | 0.8 | 0.9 |
| *Nasonia vitripennis* | 7425 | NCBI RefSeq | GCF_009193385.2 | 98.4 | 96.6 | 1.8 | 0 | 1.6 |
| *Neodiprion lecontei* | 441921 | NCBI RefSeq | GCF_001263575.1 | 99.7 | 99.3 | 0.4 | 0.1 | 0.2 |
| *Nicrophorus vespilloides* | 110193 | NCBI RefSeq | GCF_001412225.1 | 100 | 98.7 | 1.3 | 0 | 0 |
| *Nilaparvata lugens* | 108931 | NCBI RefSeq | GCF_014356525.1 | 96.3 | 90.7 | 5.6 | 0.7 | 3 |
| *Nomia melanderi* | 2448451 | NCBI RefSeq | GCF_003710045.1 | 98.8 | 98.5 | 0.3 | 0.5 | 0.7 |
| *Notodromas monacha* | 399045 | NCBI GenBank | GCA_905338405.1 | 80.7 | 76.6 | 4.1 | 4.4 | 14.9 |
| *Nylanderia fulva* | 613905 | NCBI RefSeq | GCF_005281655.1 | 98.6 | 95.8 | 2.8 | 0.1 | 1.3 |
| *Odontomachus brunneus* | 486640 | NCBI RefSeq | GCF_010583005.1 | 99.4 | 97.5 | 1.9 | 0 | 0.6 |
| *Onthophagus taurus* | 166361 | NCBI RefSeq | GCF_000648695.1 | 99.6 | 96.2 | 3.4 | 0.1 | 0.3 |
| *Ooceraea biroi* | 2015173 | NCBI RefSeq | GCF_003672135.1 | 99 | 97 | 2 | 0.1 | 0.9 |
| *Orussus abietinus* | 222816 | NCBI RefSeq | GCF_000612105.2 | 98.8 | 97.9 | 0.9 | 0.4 | 0.8 |
| *Osmia lignaria* | 473952 | NCBI RefSeq | GCF_012274295.1 | 99 | 98.7 | 0.3 | 0.2 | 0.8 |
| *Ostrinia furnacalis* | 93504 | NCBI RefSeq | GCF_004193835.1 | 98.2 | 97.1 | 1.1 | 0.9 | 0.9 |
| *Papilio xuthus* | 66420 | NCBI RefSeq | GCF_000836235.1 | 99.3 | 99 | 0.3 | 0.1 | 0.6 |
| *Pararge aegeria* | 116150 | NCBI RefSeq | GCF_905163445.1 | 99.8 | 99.3 | 0.5 | 0 | 0.2 |
| *Parasteatoda tepidariorum* | 114398 | NCBI RefSeq | GCF_000365465.3 | 98.3 | 93.4 | 4.9 | 0.8 | 0.9 |
| *Parnassius apollo* | 110799 | NCBI GenBank | GCA_907164705.1 | 96.8 | 95.6 | 1.2 | 1.3 | 1.9 |
| *Pediculus humanus corporis* | 121224 | NCBI RefSeq | GCF_000006295.1 | 96.7 | 96.4 | 0.3 | 2.5 | 0.8 |
| *Penaeus japonicus* | 27405 | NCBI RefSeq | GCF_017312705.1 | 98.1 | 94.8 | 3.3 | 0.8 | 1.1 |
| *Periplaneta americana* | 6978 | Li et al. (2018) | unavailable | 92.2 | 89.3 | 2.9 | 4.9 | 2.9 |
| *Photinus pyralis* | 7054 | NCBI RefSeq | GCF_008802855.1 | 98.8 | 85.9 | 12.9 | 0.3 | 0.9 |
| *Pieris brassicae* | 7116 | NCBI RefSeq | GCF_905147105.1 | 99.6 | 99.3 | 0.3 | 0 | 0.4 |
| *Plutella xylostella* | 51655 | NCBI RefSeq | GCF_905116875.1 | 98.4 | 97.4 | 1 | 0.2 | 1.4 |
| *Pogonomyrmex barbatus* | 144034 | NCBI RefSeq | GCF_000187915.1 | 97.2 | 96.8 | 0.4 | 2 | 0.8 |
| *Polistes canadensis* | 91411 | NCBI RefSeq | GCF_001313835.1 | 99.5 | 98.9 | 0.6 | 0 | 0.5 |
| *Polypedilum vanderplanki* | 319348 | NCBI GenBank | GCA_018290095.1 | 98.7 | 96.7 | 2 | 0.4 | 0.9 |
| *Portunus trituberculatus* | 210409 | NCBI RefSeq | GCF_017591435.1 | 97 | 96.4 | 0.6 | 1.3 | 1.7 |
| *Procambarus clarkii* | 6728 | NCBI RefSeq | GCF_020424385.1 | 98 | 96.2 | 1.8 | 1 | 1 |
| *Pseudoatta argentina* | 621737 | NCBI GenBank | GCA_017607525.1 | 91 | 90.5 | 0.5 | 1.6 | 7.4 |
| *Pseudomyrmex gracilis* | 219809 | NCBI RefSeq | GCF_002006095.1 | 99.3 | 98.1 | 1.2 | 0.1 | 0.6 |
| *Rhodnius prolixus* | 13249 | Vector Base | unavailable | 88.3 | 87.2 | 1.1 | 6 | 5.7 |
| *Rhopalosiphum maidis* | 43146 | NCBI RefSeq | GCF_003676215.2 | 98.6 | 93.1 | 5.5 | 0.3 | 1.1 |
| *Rhynchophorus ferrugineus* | 354439 | NCBI GenBank | GCA_014462685.1 | 96.1 | 93.8 | 2.3 | 2.6 | 1.3 |
| *Scaptodrosophila lebanonensis* | 7225 | NCBI RefSeq | GCF_003285725.1 | 99.6 | 97.7 | 1.9 | 0 | 0.4 |
| *Schistocerca americana* | 7009 | NCBI RefSeq | GCF_021461395.2 | 99.1 | 97 | 2.1 | 0 | 0.9 |
| *Sipha flava* | 143950 | NCBI RefSeq | GCF_003268045.1 | 96 | 94.5 | 1.5 | 0.6 | 3.4 |
| *Sitophilus oryzae* | 7048 | NCBI RefSeq | GCF_002938485.1 | 99.4 | 97.1 | 2.3 | 0.3 | 0.3 |
| *Solenopsis invicta* | 13686 | NCBI RefSeq | GCF_016802725.1 | 99.6 | 98.4 | 1.2 | 0 | 0.4 |
| *Spodoptera frugiperda* | 7108 | NCBI RefSeq | GCF_011064685.1 | 96.7 | 74.8 | 21.9 | 0.1 | 3.2 |
| *Spodoptera litura* | 69820 | NCBI RefSeq | GCF_002706865.1 | 99 | 97.6 | 1.4 | 0.5 | 0.5 |
| *Stomoxys calcitrans* | 35570 | NCBI RefSeq | GCF_001015335.1 | 99.2 | 98.4 | 0.8 | 0.3 | 0.5 |
| *Temnothorax longispinosus* | 300112 | NCBI GenBank | GCA_004794745.1 | 91.6 | 91 | 0.6 | 2.1 | 6.3 |
| *Tenebrio molitor* | 7067 | NCBI GenBank | GCA_014282415.2 | 96.3 | 95.8 | 0.5 | 0.8 | 2.9 |
| *Tetranychus urticae* | 32264 | NCBI RefSeq | GCF_000239435.1 | 91.5 | 85.2 | 6.3 | 1.7 | 6.8 |
| *Thrips palmi* | 161013 | NCBI RefSeq | GCF_012932325.1 | 97.5 | 96.3 | 1.2 | 0.1 | 2.4 |
| *Tigriopus californicus* | 6832 | NCBI GenBank | GCA_007210705.1 | 91.8 | 90.5 | 1.3 | 2.6 | 5.6 |
| *Trachymyrmex septentrionalis* | 34720 | NCBI RefSeq | GCF_001594115.1 | 99.4 | 99.2 | 0.2 | 0.1 | 0.5 |
| *Tribolium madens* | 41895 | NCBI RefSeq | GCF_015345945.1 | 99.2 | 98.7 | 0.5 | 0 | 0.8 |
| *Trichogramma pretiosum* | 7493 | NCBI RefSeq | GCF_000599845.2 | 99.2 | 96.1 | 3.1 | 0 | 0.8 |
| *Trichoplusia ni* | 7111 | NCBI RefSeq | GCF_003590095.1 | 98.6 | 90.4 | 8.2 | 0.1 | 1.3 |
| *Vanessa atalanta* | 42275 | NCBI RefSeq | GCF_905147765.1 | 99.7 | 99.5 | 0.2 | 0 | 0.3 |
| *Varroa jacobsoni* | 62625 | NCBI RefSeq | GCF_002532875.1 | 95.7 | 94.2 | 1.5 | 0.7 | 3.6 |
| *Venturia canescens* | 32260 | NCBI RefSeq | GCF_019457755.1 | 99.5 | 98.3 | 1.2 | 0 | 0.5 |
| *Vespa crabro* | 7445 | NCBI RefSeq | GCF_910589235.1 | 99.5 | 98.9 | 0.6 | 0.1 | 0.4 |
| *Vespula pensylvanica* | 30213 | NCBI RefSeq | GCF_014466175.1 | 99.4 | 99.1 | 0.3 | 0.1 | 0.5 |
| *Vollenhovia emeryi* | 411798 | NCBI RefSeq | GCF_000949405.1 | 99.8 | 99.4 | 0.4 | 0.1 | 0.1 |
| *Wasmannia auropunctata* | 64793 | NCBI RefSeq | GCF_000956235.1 | 98.9 | 98.2 | 0.7 | 0.6 | 0.5 |
| *Zaprionus bogoriensis* | 309927 | NCBI GenBank | GCA_021223985.1 | 94.3 | 93.4 | 0.9 | 0.2 | 5.5 |
| *Zerene cesonia* | 33412 | NCBI RefSeq | GCF_012273895.1 | 97 | 93.7 | 3.3 | 0.5 | 2.5 |
| *Zeugodacus cucurbitae* | 28588 | NCBI RefSeq | GCF_000806345.1 | 99.4 | 99 | 0.4 | 0.1 | 0.5 |
| *Zootermopsis nevadensis* | 136037 | NCBI RefSeq | GCF_000696155.1 | 98.4 | 98.1 | 0.3 | 0.6 | 1 |

**Supplementary Material 22** -List of articles referenced to evaluate the phylogenetic relationships among species included in the predicted phylogenomic tree.

| **Arthropoda order** | **Author** | **Publication year** | **Title** | **DOI** |
| --- | --- | --- | --- | --- |
| Blattodea | Wang et al. | 2017 | Reconstructing the phylogeny of Blattodea: Robust support for interfamilial relationships and major clades | 10.1038/s41598-017-04243-1 |
|  | Cameron et al. | 2012 | A mitochondrial genome phylogeny of termites (Blattodea: Termitoidae): Robust support for interfamilial relationships and molecular synapomorphies define major clades | 10.1016/j.ympev.2012.05.034 |
| Chelicerata | Ballesteros et al. | 2022 | Comprehensive Species Sampling and Sophisticated Algorithmic Approaches Refute the Monophyly of Arachnida | 10.1093/molbev/msac021 |
| Coleoptera | Kusy et al. | 2018 | Genome sequences identify three families of Coleoptera as morphologically derived click beetles (Elateridae) | 10.1038/s41598-018-35328-0 |
|  | Yuan et al. | 2023 | Transcriptomic data recover a new superfamily-level phylogeny of Cucujiformia (Coleoptera, Polyphaga) | 10.1016/j.ympev.2022.107679 |
|  | Cai et al. | 2022 | Integrated phylogenomics and fossil data illuminate the evolution of beetles | 10.1098/rsos.211771 |
|  | Wang et al. | 2020 | Characterization of the complete mitochondrial genome of Euwallacea fornicatus (Eichhoff, 1868) (Coleoptera: Curculionidae: Scolytinae) and its phylogenetic implications | 10.1080/23802359.2020.1827070 |
|  | Wang LJ,Wu YW,Wang TY | 2021 | Characterization of the complete mitochondrial genome of Abscondita cerata (Olivier, 1911) (Coleoptera: Lampyridae) and its phylogenetic implications | 10.1080/23802359.2021.1959456 |
|  | Liu et al. | 2017 | Complete mitochondrial genome of white-striped long-horned beetle, Batocera lineolata (Coleoptera: Cerambycidae) by next-generation sequencing and its phylogenetic relationship within superfamily Chrysomeloidea | 10.1080/23802359.2017.1361797 |
| Crustacea | Regier et al. | 2010 | Arthropod relationships revealed by phylogenomic analysis of nuclear protein-coding sequences | 10.1038/nature08742 |
| Diptera | Remsen J,O'Grady P | 2002 | Phylogeny of Drosophilinae (Diptera: Drosophilidae), with comments on combined analysis and character support | 10.1016/S1055-7903(02)00226-9 |
|  | Zhang et al. | 2019 | Mitochondrial genomes provide insights into the phylogeny of culicomorpha (Insecta: Diptera) | 10.3390/ijms20030747 |
|  | Wiegmann et al. | 2011 | Episodic radiations in the fly tree of life | 10.1073/pnas.1012675108 |
|  | Li et al. | 2022 | Phylogenomic analyses of the genus Drosophila reveals genomic signals of climate adaptation | 10.1111/1755-0998.13561 |
|  | Lambkin et al. | 2013 | The phylogenetic relationships among infraorders and superfamilies of Diptera based on morphological evidence | 10.1111/j.1365-3113.2012.00652.x |
|  | Narayanan et al. | 2019 | Phylogenomic analysis of Calyptratae: resolving the phylogenetic relationships within a major radiation of Diptera | 10.1111/cla.12375 |
|  | Feuda et al. | 2021 | Phylogenomics of Opsin Genes in Diptera Reveals Lineage-Specific Events and Contrasting Evolutionary Dynamics in Anopheles and Drosophila | 10.1093/gbe/evab170 |
| Hemiptera | Nováková et al. | 2013 | Reconstructing the phylogeny of aphids (Hemiptera: Aphididae) using DNA of the obligate symbiont Buchnera aphidicola | 10.1016/j.ympev.2013.03.016 |
|  | Song N,Zhang H,Zhao T | 2019 | Insights into the phylogeny of Hemiptera from increased mitogenomic taxon sampling | 10.1016/j.ympev.2019.05.009 |
|  | Rebijith et al. | 2017 | Reconstructing the macroevolutionary patterns of aphids (Hemiptera: Aphididae) using nuclear and mitochondrial DNA sequences | 10.1093/biolinnean/blx020 |
| Hymenoptera | Pitz et al. | 2007 | Phylogenetic relationships among the Braconidae (Hymenoptera: Ichneumonoidea): A reassessment of Shi et al. (2005) | 10.1016/j.ympev.2006.11.010 |
|  | Peters et al. | 2018 | Transcriptome sequence-based phylogeny of chalcidoid wasps (Hymenoptera: Chalcidoidea) reveals a history of rapid radiations, convergence, and evolutionary success | 10.1016/j.ympev.2017.12.005 |
|  | Zheng et al. | 2021 | Comparative mitogenomics and phylogenetics of the stinging wasps (Hymenoptera: Aculeata) | 10.1016/j.ympev.2021.107119 |
|  | Boudinot BE,Borowiec ML,Prebus MM | 2022 | Phylogeny, evolution, and classification of the ant genus Lasius, the tribe Lasiini and the subfamily Formicinae (Hymenoptera: Formicidae) | 10.1111/syen.12522 |
|  | Wharton et al. | 1992 | PHYLOGENY OF THE SUBFAMILIES OF THE FAMILY BRACONIDAE (HYMENOPTERA: ICHNEUMONOIDEA): A REASSESSMENT | 10.1111/j.1096-0031.1992.tb00068.x |
|  | Ward PS,Blaimer BB,Fisher BL | 2016 | A revised phylogenetic classification of the ant subfamily Formicinae (Hymenoptera: Formicidae), with resurrection of the genera Colobopsis and Dinomyrmex | 10.11646/zootaxa.4072.3.4 |
|  | Solomon et al. | 2019 | The molecular phylogenetics of Trachymyrmex Forel ants and their fungal cultivars provide insights into the origin and coevolutionary history of ‘higher-attine’ ant agriculture | 10.1111/syen.12370 |
|  | Schmidt C | 2013 | Molecular phylogenetics of ponerine ants (Hymenoptera: Formicidae: Ponerinae) | 10.11646/zootaxa.3647.2.1 |
|  | Munro et al. | 2011 | A molecular phylogeny of the chalcidoidea (hymenoptera) | 10.1371/journal.pone.0027023 |
|  | Ward et al. | 2015 | The evolution of myrmicine ants: Phylogeny and biogeography of a hyperdiverse ant clade (Hymenoptera: Formicidae) | 10.1111/syen.12090 |
|  | Mueller et al. | 2018 | Phylogenetic patterns of ant–fungus associations indicate that farming strategies, not only a superior fungal cultivar, explain the ecological success of leafcutter ants | 10.1111/mec.14588 |
|  | Danforth et al. | 2013 | The impact of molecular data on our understanding of bee phylogeny and evolution | 10.1146/annurev-ento-120811-153633 |
|  | Sharkey et al. | 2012 | Phylogenetic relationships among superfamilies of Hymenoptera | 10.1111/j.1096-0031.2011.00366.x |
|  | Moreau CS,Bell CD | 2013 | Testing The Museum Versus Cradle Tropical Biological Diversity Hypothesis: Phylogeny, Diversification, And Ancestral Biogeographic Range Evolution Of The Ants | 10.1111/evo.12105 |
|  | Ward PS | 2014 | The phylogeny and evolution of ants | 10.1146/annurev-ecolsys-120213-091824 |
|  | Debevec AH,Cardinal S,Danforth BN | 2012 | Identifying the sister group to the bees: A molecular phylogeny of Aculeata with an emphasis on the superfamily Apoidea | 10.1111/j.1463-6409.2012.00549.x |
| Lepidoptera | Kawahara et al. | 2019 | Phylogenomics reveals the evolutionary timing and pattern of butterflies and moths | 10.1073/pnas.1907847116 |
|  | Freitas AV,Brown KS | 2004 | Phylogeny of the Nymphalidae (Lepidoptera) | 10.1080/10635150490445670 |
|  | Riyaz et al. | 2021 | Comparative mitochondrial genome analysis of Eudocima salaminia (Cramer, 1777) (Lepidoptera: Noctuoidea), novel gene rearrangement and phylogenetic relationship within the superfamily Noctuoidea | 10.1007/s11033-021-06465-z |
|  | Rota et al. | 2022 | The unresolved phylogenomic tree of butterflies and moths (Lepidoptera): Assessing the potential causes and consequences | 10.1111/syen.12545 |
|  | Shi et al. | 2015 | Morphological characters are compatible with mitogenomic data in resolving the phylogeny of nymphalid butterflies (Lepidoptera: Papilionoidea: Nymphalidae) | 10.1371/journal.pone.0124349 |
|  | Léger et al. | 2021 | Refining the phylogeny of Crambidae with complete sampling of subfamilies (Lepidoptera, Pyraloidea) | 10.1111/zsc.12452 |
|  | Zhu et al. | 2018 | The first mitochondrial genomes for Pyralinae (Pyralidae) and Glaphyriinae (Crambidae), with phylogenetic implications of Pyraloidea | 10.1371/journal.pone.0194672 |
|  | Breeschoten et al. | 2022 | Expanding the Menu: Are Polyphagy and Gene Family Expansions Linked across Lepidoptera? | 10.1093/gbe/evab283 |

**Supplementary Material 23** – Proposed phylogenomic tree of Hexapoda, inferred using a constraint-based analysis of single-copy genes. Colored boxes represent the presence of specific genes in the genomes of the corresponding species. The nodes including the Chelicerata and Crustacea species used as outgroups were collapsed to facilitate the visualization.

**
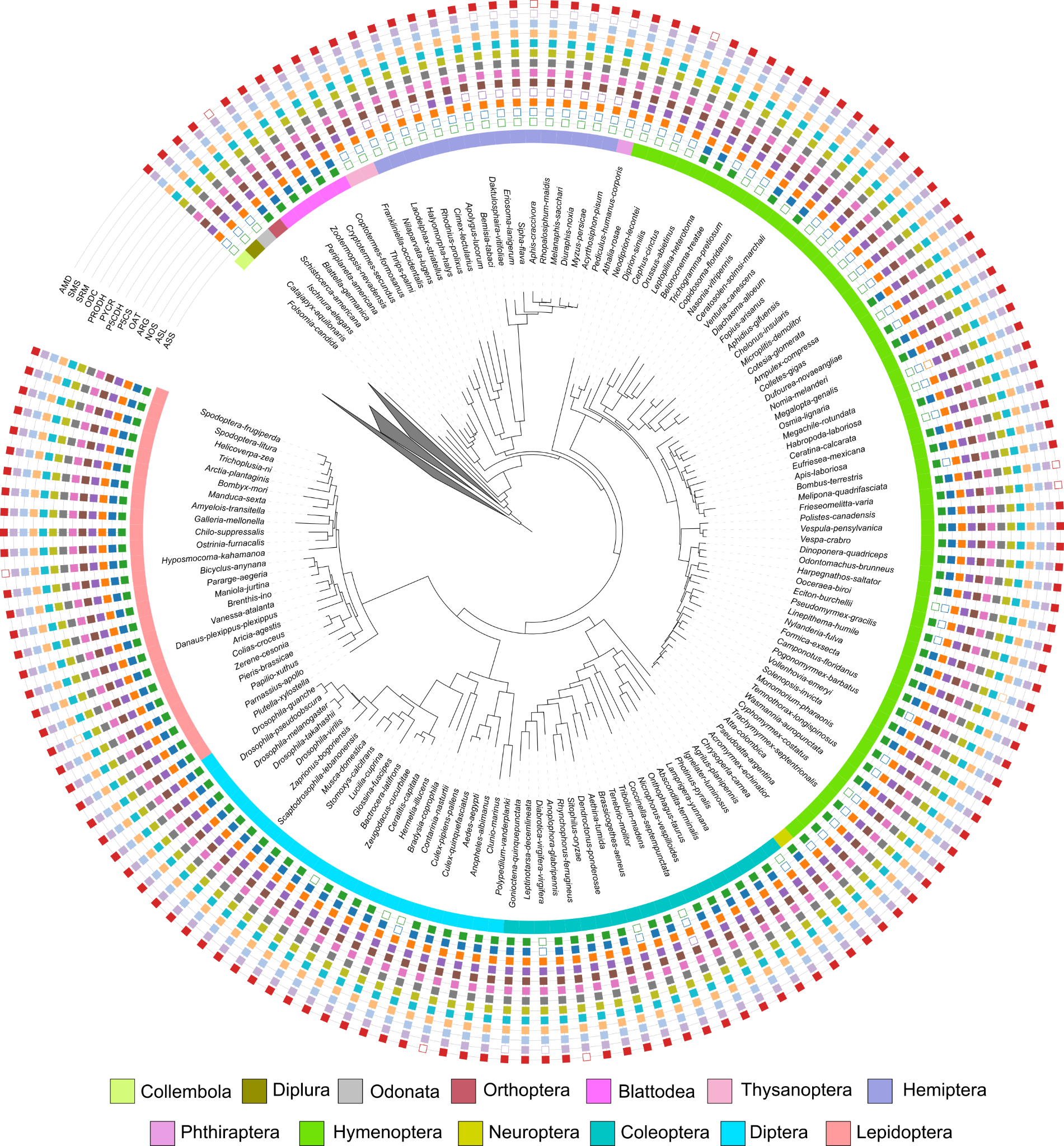
**

**Supplementary Material 24** - List of sequences and active site residues used to filter the enzymes described in the manuscript.

| **Abbre-viation** | **Name** | **EC Number** | **KO ID** | **UNIPROT ID** | **Active Site residues** |
| --- | --- | --- | --- | --- | --- |
| CPS | carbamoyl-phosphate synthase (ammonia) | 6.3.4.16 | K01948 | absent | absent |
| OTC | ornithine carbamoyltransferase | 2.1.3.3 | K00611 | Q9N1U7 | 303-C/ 263-D |
| ASS | argininosuccinate synthase | 6.3.4.5 | K01940 | absent | absent |
| ASL | argininosuccinate lyase | 4.3.2.1 | K01755 | P04424 | 160-H/ 281-S |
| NOS | nitric-oxide synthase (NADPH) | 1.14.13.39 | K13240 (brain) | absent | absent |
| ARG | arginase | 3.5.3.1 | K01476 | absent | absent |
| OAT | ornithine aminotransferase | 2.6.1.13 | K00819 | absent | absent |
| P5CS | delta-1-pyrroline-5-carboxylate synthetase | 2.7.2.11/ 1.2.1.41 | K12657 | absent | absent |
| P5CDH | delta-1-pyrroline-5-carboxylate dehydrogenase | 1.2.1.88 | K00294 | P30038 | 314E/ 348C |
| PYCR | pyrroline-5-carboxylate reductase | 1.5.1.2 | K00286 | absent | absent |
| PRODH | proline dehydrogenase | 1.5.5.2 | K00318 | absent | absent |
| ODC | ornithine decarboxylase | 4.1.1.17 | K01581 | P11926 | 360C |
| SRM | spermidine synthase | 2.5.1.16 | K00797 | P19623 | 173D |
| SMS | spermine synthase | 2.5.1.22 | K00802 | P52788 | 276D |
| AMD | adenosylmethionine decarboxylase | 4.1.1.50 | K01611 | P17707 | 8E/11E/68S/ 82C/229S/243H |

**Supplementary Material 25** – Insects rearing strategies for RNA-seq analysis.

| **Species** | **Stage** | **Condition** | **Diet** |
| --- | --- | --- | --- |
| *Periplaneta americana* | Adult | Laboratory | Oat and chayote |
| *Rhodnius prolixus* | Adult | Laboratory | Rabbit blood |
| *Tenebrio molitor* | Larvae | Laboratory | Wheat bran |
| *Musca domestica* | Larvae | Laboratory | Mixture of pig food and rice hull |
| *Spodoptera frugiperda* | Larvae | Laboratory | Artificial diet according Parra (1986) |
| *Abracris flavolineata* | Adult | Field | Collard leaves |
| *Dermestes maculatus* | Larvae | Laboratory | Small vertebrate carcasses |
| *Dysdercus peruvianus* | Adult | Laboratory | Cotton seeds |
| *Mahanarva fimbriolata* | Adult | Field | No described |
